# Evolutionary origins of Brassicaceae specific genes in *Arabidopsis thaliana*

**DOI:** 10.1186/1471-2148-11-47

**Published:** 2011-02-18

**Authors:** Mark TA Donoghue, Channa Keshavaiah, Sandesh H Swamidatta, Charles Spillane

**Affiliations:** 1 Genetics & Biotechnology Lab, Department of Biochemistry, Lee Maltings 2.10, University College Cork (UCC), Cork, Ireland; 2 Genetics & Biotechnology Lab, Botany & Plant Science, Aras de Brun C306, National University of Ireland Galway (NUIG), Galway, Ireland

## Abstract

**Background:**

All sequenced genomes contain a proportion of lineage-specific genes, which exhibit no sequence similarity to any genes outside the lineage. Despite their prevalence, the origins and functions of most lineage-specific genes remain largely unknown. As more genomes are sequenced opportunities for understanding evolutionary origins and functions of lineage-specific genes are increasing.

**Results:**

This study provides a comprehensive analysis of the origins of lineage-specific genes (LSGs) in *Arabidopsis thaliana *that are restricted to the Brassicaceae family. In this study, lineage-specific genes within the nuclear (1761 genes) and mitochondrial (28 genes) genomes are identified. The evolutionary origins of two thirds of the lineage-specific genes within the *Arabidopsis thaliana *genome are also identified. Almost a quarter of lineage-specific genes originate from non-lineage-specific paralogs, while the origins of ~10% of lineage-specific genes are partly derived from DNA exapted from transposable elements (twice the proportion observed for non-lineage-specific genes). Lineage-specific genes are also enriched in genes that have overlapping CDS, which is consistent with such novel genes arising from overprinting. Over half of the subset of the 958 lineage-specific genes found only in *Arabidopsis thaliana *have alignments to intergenic regions in *Arabidopsis lyrata*, consistent with either *de novo *origination or differential gene loss and retention, with both evolutionary scenarios explaining the lineage-specific status of these genes. A smaller number of lineage-specific genes with an incomplete open reading frame across different *Arabidopsis thaliana *accessions are further identified as accession-specific genes, most likely of recent origin in *Arabidopsis thaliana*. Putative *de novo *origination for two of the *Arabidopsis thaliana*-only genes is identified via additional sequencing across accessions of *Arabidopsis thaliana *and closely related sister species lineages. We demonstrate that lineage-specific genes have high tissue specificity and low expression levels across multiple tissues and developmental stages. Finally, stress responsiveness is identified as a distinct feature of Brassicaceae-specific genes; where these LSGs are enriched for genes responsive to a wide range of abiotic stresses.

**Conclusion:**

Improving our understanding of the origins of lineage-specific genes is key to gaining insights regarding how novel genes can arise and acquire functionality in different lineages. This study comprehensively identifies all of the Brassicaceae-specific genes in *Arabidopsis thaliana *and identifies how the majority of such lineage-specific genes have arisen. The analysis allows the relative importance (and prevalence) of different evolutionary routes to the genesis of novel ORFs within lineages to be assessed. Insights regarding the functional roles of lineage-specific genes are further advanced through identification of enrichment for stress responsiveness in lineage-specific genes, highlighting their likely importance for environmental adaptation strategies.

## Background

Lineage-specific genes (LSGs) are defined as protein encoding genes that have no significant sequence similarity to any other proteins/peptides in the databases [1-3]. LSGs are also called orphan genes or ORFans [1-3]. LSGs can include paralogous families of genes found within a species or orthologous taxonomically restricted genes (TRGs) [2] that are only found within a specific clade of closely related species. Such LSGs are a significant component of all genomes sequenced to-date [4], and have been identified in all domains of biological life, including in viruses [2,3,5-10]. LSGs were initially thought to be simply an artefact of limited extent of genome sequencing across many biological lineages [11]. However, the number of LSGs has continued to increase as more genome sequence data for multiple taxa has become available. Indeed, there is a linear or nearly linear relationship between the number of sequences added to the sequence databases and novel protein family discovery [2,12].

Lineage-specific genes pose a particular challenge for both bioinformtic and wet-lab based approaches to their functional characterization. The lack of homology to other genes means that homology-based functional classifications are not possible, which renders the majority of homology or evolutionary conservation based bioinformatic approaches redundant. However, the genomic features and context of LSGs can provide some preliminary clues regarding the possible modes of evolution of LSGs. To date, some general genomic characteristics that have been identified for LSGs include; short length, fewer introns, atypical GC content, and increased evolutionary rates [5-7,13,14].

Genomic novelty can arise via duplication (including retrotransposition) and subsequent sequence divergence of (one copy of) the gene leading to neo- or sub-functionalization [15,16]. Although origins due to increased evolutionary rates of duplicated LSGs is one possible model, there are other mechanisms of gene origin that could result in novel open reading frames that encode proteins with no sequence similarity to other proteins. These mechanisms include transposon exaptation and *de novo *origination (i.e. the origin of a new gene from non-coding sequence via mutations) [17]. Such mechanisms have been demonstrated to generate novel genes in several species, for example; (a) via retrotransposons in rice, maize [18], poplar and *Arabidopsis thaliana *[19] and primates [20]; and (b) via transposon exaptation in *Arabidopsis thaliana *[21] and primates [7]*. De novo *origination of novel genes from non-coding sequences has been observed for a small number of genes in primates [7,22], *Drosophila *[23], rice [24] and yeast [25]. However, to date only a small number of studies have systematically classified the evolutionary modes responsible for the "birth" of LSGs for a given lineage, for example Zhou *et al *in *Drosophila *[26], and Toll-Riera *et al *in primates [7]. In this regard, there have been no studies to systematically assess the range of evolutionary modes responsible for the origin of the majority of LSGs within any plant species.

Recent studies have identified a cohort of genes specific to Brassicaceae and *Arabidopsis thaliana *and highlighted some important features of such LSGs (see results) [27,28]. In this study, an independent analysis of the genome-wide complement of LSGs combined with a comprehensive elucidation of the modes of evolutionary origin of the majority of LSGs in *Arabidopsis thaliana *was performed. This is achieved by identifying the genomic context of LSGs in the genome of *Arabidopsis thaliana *and other species. Our study highlights possible mechanisms of origination that are responsible for generating LSGs in *Arabidopsis thaliana*, and the relative extent by which each origination mechanism is used to produce such LSGs.

The approach used in this study to define LSGs has an emphasis on comparing all of the annotated protein-coding genes in the *Arabidopsis thaliana *genome to as many existing sequences as possible thereby further reducing false positives and eliminating genes (not listed in the UniProt database) that might have been horizontally transferred into *Arabidopsis thaliana*, as these would not meet the definition of lineage-specificity. Additionally, to further screen for sequence similarity between sequences, we have employed position-specific methods that can detect weaker homologous relationships that would otherwise be missed by the standard BLAST algorithms.

In contrast to previous LSG studies, which focussed only on the nuclear genome, in this study the mitochondrial and chloroplast genomes were also screened for lineage-specific genes. Finally, we have identified striking responses of lineage-specific genes to environmental stimuli in *Arabidopsis thaliana *and consider these in relation to proposed evolutionary models for the birth and functionality of novel genes.

## Results

To identify lineage-specific gene models which are restricted to the Brassicaceae family a step-wise BLAST filtering [29] approach was used against several databases (including NCBI databases nr, nt and est) using the BLASTP, TBLASTN and TBLASTN programs respectively. Next, PSIBLAST was used to identify matches missed by the other BLAST programs (using the nr database). A final filtering step using InterProScan [30] was also performed. The results of the number of Brassicaceae family specific LSGs returned at each filtering stage are presented in Additional file 1. To determine the sensitivity of the of the dataset to the choice of E-value cut-off, each BLAST search step was tested using E-values ranging from 10e-20 to 1 (Additional file 2).

Using an E-value cut-off of 10e-3, 1789 (including 28 mitochondrial) Brassicaceae specific genes were identified, which contain a subset of 958 (including 18 mitochondrial) genes that are *Arabidopsis thaliana *specific. The proportions of gene models tested that are LSGs on each chromosome of *Arabidopsis thaliana *are as follows; 5.84%, 7.86%, 6.87%, 6.07% and 6.36% for chromosomes one, two, three, four and five respectively, suggesting no major bias of LSGs to any particular chromosomes. The proportion of LSGs identified in the mitochondrial genome is 22.95%. No Brassicaceae specific genes are found in the chloroplast genome. These numbers differ slightly to the previous studies of *Arabidopsis thaliana *LSGs mainly due to the databases searched and the additional use (in this study) of position specific methods (that can detect homologous relationships missed by standard BLAST methods) [29,30], rather than being due to differences in the E-value cut-off. For example, in this study an E-value cut-off of 10e-5 only reported an additional 34 LSGs indicating that E-value cut-off is not a major determinant of the overall proportion of LSGs found in the *Arabidopsis thaliana *genome (Additional file 3).

Despite differences in the exact number of lineage-specific genes reported, many of the genomic features of LSGs previously reported by Lin *et al *[28] were also identifiable in our dataset. These include short peptide length, fewer introns, lower GC content, many genes of unknown function, fewer paralogs, fast evolving genes, enrichment for secretory peptides, and a gene set enriched for defensin-like-genes and other cysteine rich peptides. The results of these genomic feature associations are presented in Additional file 3. A full list of the LSGs within the *Arabidopsis thaliana *genome identified in this study is also provided in Additional file 4.

LSGs have been shown to be fast evolving in an earlier study [28] and our analysis also indicates that LSGs are fast evolving (Additional file 3). While rapid evolution of a subset of LSGs may account for their lineage specific status, it does not necessarily explain the origins of all such LSG genes in the *Arabidopsis thaliana *genome. Therefore with the comprehensive set of LSGs we defined in *Arabidopsis thaliana*, we set out to elucidate the possible origins of LSGs in the *Arabidopsis thaliana *genome. To do this we systematically tested a number of different evolutionary scenarios (each of which we hypothesised could be responsible for the origin of an LSG) by identifying the genomic contexts of the LSGs in the genome of *Arabidopsis thaliana *and other plant species, and inferred from this approach the likely mechanism of origin of an LSG and/or why the gene in question is identified as an LSG. The evolutionary scenarios we tested as potential mechanisms for generation of a novel LSG included; (1) overprinting at a conserved gene locus; (2) duplication followed by divergence; (3) transposon exaptation, and; (4) *de novo *origination (which is also consistent with differential loss across species). The first three involve identifying sequence matches within the genome of *Arabidopsis thaliana*; in contrast the fourth involves identifying nucleotide sequence matches (either non-coding or in a different reading frame) to non-Brassicaceae plant genomes.

### LSGs are over-represented in overlapping ORFs compared to non-LSGs

The first mechanism of origin for LSGs tested for was overprinting. Overprinting describes a mechanism that creates new genes via mutations occurring within a coding sequence that lead to the expression of a novel protein in another reading frame that overlaps the existing parental gene [31,32]. Hence, if an LSG overlaps a non-LSG ORF, the evolutionary origin of the LSG can be pinpointed to an overprinting event at the non-LSG parental gene (note, however not all overlapping genes in the *Arabidopsis thaliana *genome contain a LSG in the pair). To conduct this analysis, the total number of all gene models with overlapping CDS was defined. Importantly, as LSG status is defined based on the peptide sequence only overlapping CDSs (not introns or UTRs) were considered.

**Figure 1:**
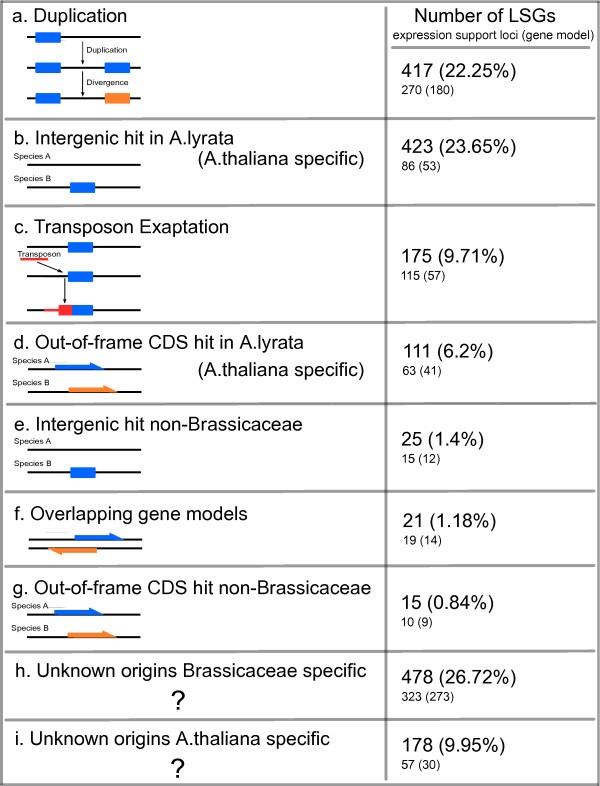
**Summary of evidence for evolutionary origins of *Arabidopsis thaliana *lineage-specific genes**. The number of LSGs that fit each evolutionary scenario tested, plus the number of LSGs without elucidated origins. Support for gene model expression provided by an EST or cDNA consistent with the of gene model (as listed by TAIR). Support of expression at the locus provided by EST, cDNA or microarray probeset (TAIR and Ath1 affymetrix microarray).

In total, 105 gene models in the *Arabidopsis thaliana *genome have overlapping CDS with another gene model (31 of which are LSGs, while 74 are non-LSGs). Twenty-one LSGs overlap with 21 non-LSGs, which are presented in Additional file 5), while ten LSGs overlap with other LSGs. Twenty-six (out of 1761) nuclear genome LSGs overlap with the CDS of other gene models. In comparison, 68 (out of 25234) non-LSG models overlap with the CDS of other gene models in the nuclear genome, indicating that LSGs are enriched for overlapping CDS in the nuclear genome (hypergeometric test, p < 0.01). In contrast, we found that LSGs are not enriched for overlapping CDS in the mitochondrial genome. Whilst overlapping CDS are enriched in LSGs, this model of gene evolution only accounts for 21 of the LSGs within the *Arabidopsis thaliana *genome i.e. only 1.18% of all LSGs (Figure 1) indicating that overprinting of existing CDS sequence is a relatively rare mechanism for generation of novel LSGs in *Arabidopsis thaliana*.

### Duplication of non-LSGs accounts for the evolutionary origins of almost one quarter of all LSGs in the genome

Gene duplication is one of the best-understood mechanisms for generation of novel genes that can acquire novel functions. Indeed, several types of duplication events have been identified and can be broadly defined into three groups. (1) Segmental duplications including local tandem duplications, (2) whole genome duplications (WGD) and (3) transposition events [33]. To test whether any of the LSGs in the *Arabidopsis thaliana *genome originated by any of the above duplication mechanisms we first identified any LSG:non-LSG paralog pairs. By identifying a significant hit to an evolutionarily conserved gene the possible origin of an LSG can be traced to that conserved "parental" gene. The duplication of a parental gene followed by the further mechanisms of point mutations and/or indels leading to frameshifts have the potential to produce ORFs that encode for novel proteins. In this study, both peptide level searches and CDS searches were performed to investigate such possibilities. Peptide level searches are more sensitive but will miss the out-of-frame matches, however these may be identified by nucleotide level CDS searches. Hits to the query sequence, other LSGs and overlapping gene models were filtered out and in-frame paralogs were identified for the CDS searches.

Using a liberal E-value cut off of 10e-3, two hundred and twenty-five LSGs have significant BLASTP hits to a non-LSG in the same reading frame, indicating a process of gene duplication followed by point mutations for these particular LSGs. Of these 225 LSGs, 32 have additional out-of-frame hits to non-LSG CDS, indicating additional mechanisms such indel mutations leading to frame-shifts. A further 173 LSGs that do not have a significant hit to a non-LSG gene at the peptide level have out-of-frame CDS hits (BLASTN, e < 0.01) to non-LSGs in *Arabidopsis thaliana*.

The median percentage of LSG sequence coverage of the alignments for the peptide level searches was 74.40 ± 24.7609 compared to the CDS alignment searches with a median coverage of 26.45 ± 15.28 (Note: the measure of spread reported using median values here and elsewhere in the study refers to the semi-interquartile range). The percentage coverage of the alignments suggests that (a) either sequence similarity at the nucleotide level has diverged to such an extent that only partial matches are identifiable, or (b) flanking genomic DNA has possibly been recruited to produce new ORFs. By using a stricter E-value fewer significant hits are returned, however the majority of the alignments reported in this study have an E-value cut-off much lower than 10e-3 (see E-value distribution of LSG BLASTP and BLASTN hits to non-LSGs in Additional file 6). The details and coordinates of each alignment are also provided in Additional file 7.

In total, eighty of the out-of-frame CDS BLAST hits are in an inverted orientation leading in these cases to the generation of a novel LSG coding in a different reading frame from its progenitor parental gene. The remaining out-of-frame CDS hits were in the same orientation indicating a role for indels leading to frame-shifts and subsequent origination of novel LSGs.

To distinguish those LSG:non-LSG pairs which have originated from large scale segmentally duplicated blocks, paralogous syntelogs in *Arabidopsis thaliana *were identified using SynMap (powered by DAGchainer [34]) part of the CoGe package [35]. Syntelogs represent a special case of gene homology where sets of genes are derived from the same ancestral genomic region. In total, seven LSG:non-LSG paralog pairs were identified in the sets of syntelogs in the *Arabidopsis thaliana *genome. Three of these were identified as in-frame paralogs within the segementally duplicated blocks, and a further four identified as out-of-frame BLASTN hits.

Using the Bowers *et al *dataset [36] one of the LSG:non-LSG paralog pairs (AT4G23870.1, AT4G11020.1) could be identified as part of the *At-*α WGD event that occurred (according to the latest estimates) 23.3 million years ago, therefore making the *At-*α duplication itself Brassicaceae specific [37]. The remaining LSG:non-LSG paralog pairs, found in syntelog groupings, are not linked to any of the three WGD events identified in the Bower *et al *dataset therefore indicating duplication events which are independent of WGD. In addition, using SynMap we identified 71 LSG:non-LSG pairs that were the result of local tandem duplications, while the remaining 320 LSG:non-LSG pairs represent distal duplications.

Retrotransposition and unequal crossing over are different duplication mechanisms that can be distinguished between as possible sources of novel LSGs. By identifying alignments that cross intron-exon boundaries we were able to distinguish between retrotransposition and unequal crossing over as (duplication) mechanisms of origin of LSGs in the *Arabidopsis thaliana *genome. Our results indicate that eight LSGs are consistent with duplication via retrotransposition, while seventy-two LSGs display evidence of duplication due to unequal crossing over. For the remaining LSGs with BLAST hits to non-LSGs no intron-exon boundaries are present in the alignments therefore it was not possible in the case of these LSGs to distinguish between each mechanism (i.e. retrotransposition vs. unequal crossing over).

As previously shown LSGs can have a higher evolutionary rate than non-LSGs [28] and this is confirmed in this study via the alternative method of *d*_*N*_/*d*_*S *_analysis between orthologous pairs of genes between *Arabidopsis thaliana *and *Arabidopsis lyrata *(Additional file 3). Therefore, it could be expected that the LSGs situated within LSG:non-LSG paralog pairs would have higher evolutionary rates compared to the non-LSGs in the paralog pairs. Using pairwise alignments between reciprocal BLAST hits of *Arabidopsis thaliana *and *Arabidopsis lyrata *(see Additional file 3 for details) *d*_*N*_/*d*_*S *_ratios for LSGs (median 0.5598 ± 0.2373) were indeed found to be higher than non-LSGs (median 0.1772 ± 0.0968). Taking the subset of LSG:non-LSG in-frame paralog pairs with calculated *d*_*N*_/*d*_*S *_ratios (n = 83), the LSGs within the pairs are observed to have a higher *d*_*N*_/*d*_*S *_ratio (median 0.5878 ± 0.2256) when compared to their non-LSG paralogs (median 0.3634 ± 0.2131). Interestingly, whilst the *d*_*N*_/*d*_*S *_ratio of the LSGs within the paralog pairs is equivalent to that of the overall *d*_*N*_/*d*_*S *_ratio of LSGs, the non-LSG paralogs within the pairs have higher *d*_*N*_/*d*_*S *_ratios when compared to the overall *d*_*N*_/*d*_*S *_ratio of non-LSGs. This suggests that a group of non-LSGs with an increased evolutionary rate have a higher potential to generate fast-evolving LSGs. Hence, it is possible that the generation of a novel LSG via rapid evolution is more likely when the parental duplicate gene (which can be a non-LSG) is already experiencing elevated rates of evolution. Overall, this study indicates that duplication of non-LSGs can account for the evolutionary origins of 22.25% of LSGs in the genome (Figure 1).

### Exaptation of transposons occurs at a higher incidence in LSGs compared to non-LSGs

Transposons can rearrange DNA sequence tracts and generate novel variation in genomes [38,39]. Indeed, much of the so called disposable genome (i.e those portions of an organism's genome composed of partially shared and strain-specific DNA sequence elements) is made up of transposable elements in plant genomes [40]. In particular, it is estimated that approximately 10% of the *Arabidopsis thaliana *genome is derived from transposable elements [41]. Exaptation describes a process in which a feature acquires a function for which it was not originally adapted or selected. In this context, transposon exaptation represents an evolutionary mechanism by which novel genes can be generated within genomes and has the potential to generate LSGs [21].

LSGs are defined on the basis of displaying no significant sequence similarity to any other peptide sequence outside of the lineage. However, LSGs may contain DNA derived from other DNA sources such as transposable element (TE) DNA or intergenic DNA. For instance, exonization of TEs can potentially occur at either the 5' or 3' ends of a TE. In addition, exonization can occur within a TE so that the novel ORF is located within the TE. Finally, a TE can be inserted into a CDS as a TE cassette, leading to novel coding sequence or intronic sequence. In most cases, TE exaptation does not account for the origin of a complete ORF indicating that either intergenic DNA is also recruited with the TE, and/or TEs are incorporated into existing ORFs. If an LSG contains co-opted non-coding DNA then no amino acid sequence similarity to existing proteins could be expected (at least for that portion of the gene). Therefore TE exaptation has the potential to make a significant contribution to LSG origination. In support of this, exaptation of TEs in LSGs in primates has been shown to be significantly higher in LSGs compared to non-LSGs [7].

**Figure 2:**
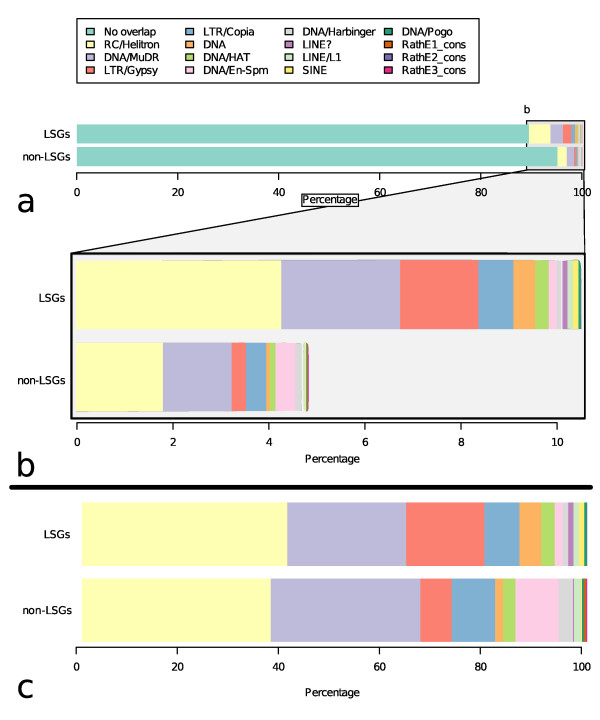
**Summary of transposon exaptation frequency in LSG and non-LSG CDS**. a) The frequency of exaptation of each TE super-family for all genes; split into LSG and non-LSGs. b) Close up view of a) where there is evidence of TE exaptation in LSGs and non-LSGs. c) The frequency of each TE super-family exapted in those genes containing exapted TE DNA, split into LSG and non-LSGs. Note, some genes have exapted DNA from several super-families, each case is reported therefore the total percentage is marginally over 100% to reflect this.

To identify all genes in the *Arabidopsis thaliana *genome with exapted TE DNA the genomic coordinates of all exon sequences of protein coding genes and TE sequences were compared, and all instances where TEs and exons overlap were identified. We determined that 175 LSGs overlap with two hundred and nineteen separate TEs (Figure 2a &2b) across 248 individual LSG exons. One hundred and seventy-one (9.78%) of all LSGs overlap TEs in the nuclear genome of *Arabidopsis thaliana*. In comparison, 1166 non-LSGs (4.62%) overlap TEs indicating that LSGs are enriched for overlaps with TEs (hypergeometric test, p < 0.01). No such enrichment of LSGs for TEs was found for the mitochondrial genome.

There are many different classes of transposons in the *Arabidopsis thaliana *genome [42]. The identification of which transposons were most associated with genesis of novel LSGs can provide insights into transposon-based mechanisms for generation of novel ORFs. Amongst all of the TE classes in the *Arabidopsis thaliana *genome, helitrons contribute most to LSG content with 90 helitron TE fragments exapted, representing 40.64% of all of the cases of TE exaptation identified in this study (Figure 2c). The next most frequent TE super-family, which is exapted to generate novel LSGs, are DNA/MuDR transposons with 48 exapted TE fragments (23.53% of total TE exaptations). This was followed by LTR/Gypsy and LTR/Copia TEs, which contribute 38 (15.51%) and 19 (6.95%) TE fragments to LSG content. The remaining TE super-families each contribute less than 5% each to the total number of exapted TE fragments (Figure 2c). A similar distribution of TE exaptation is found in non-LSGs, with helitron, DNA/MuDR, LTR/Gypsy and LTR/Copia super-families contributing mostly to exapted TE DNA. However unlike LSGs, in the case of non-LSGs the DNA/En-Spm super-family also contributes to more than 5% of cases of exaptation of non-LSGs. Overall, our study indicates that TE exaptation to generate novel ORFs can account for the evolutionary origins of 9.71% (Figure 1) of all LSGs in the *Arabidopsis thaliana *genome, with helitrons and DNA/MuDR TEs representing approximately two-thirds of all such cases.

Although the rolling-circle (RC) helitrons contribute the most DNA in terms of actual numbers of LSGs containing helitron DNA, the actual relative proportion of helitrons that contribute DNA to the CDS of any ORF is in fact very small (Additional file 8) [21]. When compared to other TE super-families, helitrons are the most prevalent of the TE super-families in the *Arabidopsis thaliana *genome in terms of the total number of TE fragments found (Additional file 8). Their relative abundance (compared to other TE super-families) may account for the finding that helitrons are the most significant contributors to both non-LSG and LSG CDS content.

### A small number of LSGs display possible chimeric origins

If a gene contains DNA compiled from several existing genomic structures/features it can be considered chimeric. Using this definition, our study finds that 54 LSGs display possible chimeric origins. 14 LSGs have non-overlapping CDS and/or peptide hits to two or three genes that don't display any homology to each other (BLASTP, e < 0.01). Two obvious possibilities in terms of the mechanisms of origin of these LSGs are available; either the ancestral genes do not share a common ancestor (i.e. non-homologous) or all the genes (i.e. the ancestral and LSGs) are all highly divergent members of the same gene family. We have also detected chimeric LSGs that contain a combination of non-LSG duplicate gene sequence and exapted TE DNA. Thirty-eight LSGs have hits to non-LSGs and also overlap with a transposable element, in each case the TE overlap and BLAST hits are for different parts of the LSG. Finally, two LSGs overlap with non-LSGs, one of which has a BLAST hit to another non-LSG in another region of its sequence and the other has exapted an LTR/Gypsy TE fragment as its second exon. Overall, our results demonstrate that some LSGs in the *Arabidopsis thaliana *genome originate from chimeric fusions of DNA sequence from different regions of the genome, possibly mediated by TEs.

### Over 2% of LSGs display homology to intergenic or out-of-frame CDS in non-Brassicaceae species

Having identified the possible origins of LSGs by analysing the genomic context of the LSGs within the *Arabidopsis thaliana *genome (i.e. overlapping genes, duplicates and TE exaptation), we next tested for any evidence of origins of LSGs derived from non-coding DNA sequence from non-Brassicaceae species. This was done by screening for BLASTN matches to either (out-of-frame) CDS or intergenic sequences of non-Brassicaceae species (see Additional file 3 for results and further details). In total, only 15 LSGs (0.84%) of all LSGs tested displayed a significant match to non-Brassicaceae CDS sequence (Figure 1). Furthermore, only 25 (1.4%) of all of the LSGs tested displayed any significant match to non-Brassicaceae intergenic DNA (Figure 1). It is worth noting that these hits were not well conserved across the species tested and the alignments cover less than 50% of the LSGs with most alignments covering 10-20%. The low percentage coverage of the alignments between the LSGs and the non-Brassicaceae genomes is indicative of the evolutionary distance between *Arabidopsis thaliana *and the other non-Brassicaceae species tested. For instance, the genome of the closest plant relative screened was papaya which last shared a common ancestor with *Arabidopsis thaliana *~72 million years ago [43]. Overall, these results suggest that only a small proportion (i.e. ~ 2%) of LSGs in *Arabidopsis thaliana *have origins that can be identified via non-coding DNA or out-of-frame CDS hits in non-Brassicaceae species (Figure 1).

### 534 Brassicaceae-specific LSGs can be traced to intergenic or out-of-frame CDS hits in the *Arabidopsis lyrata *genome

Having determined that the evidence for *de novo *origins of LSGs in non-Brassicaceae species was low (~2%), we next investigated possible origins of those LSGs that are specific to the species *Arabidopsis thaliana*. By identifying the genomic context of *Arabidopsis thaliana*-only LSGs in the sister species *Arabidopsis lyrata *we considered that it should be possible to gain insights into more recent origins of some LSGs.

To identify putative *Arabidopsis thaliana*-only LSGs all Brassicaceae restricted LSGs (n = 1789) were BLASTP searched against all *Arabidopsis lyrata *gene models. Nine hundred and fifty-eight (53.55%) LSGs have no hit to *Arabidopsis lyrata *at the peptide level. Of these 958 LSGs, the origins of 246 have already been identified in this study. Of the remaining 712, 111 (6.2% of the total, including 31 syntelogs) have out-of-frame hits to *Arabidopsis lyrata *CDS and 423 (23.65%, including eight syntelogs) have hits to scaffold sequences in *Arabidopsis lyrata *(Figure 1). Of the remaining *Arabidopsis thaliana-*only LSGs, nine have CDS or scaffold hits on the *Arabidopsis lyrata *genome covering less than 10% of the LSGs, while 169 have no hits at all in the *Arabidopsis lyrata *genome. Therefore, using this approach to define LSGs that are specific to the *Arabidopsis thaliana *lineage, we can report 178 LSGs (9.95% of the total) as having no significant match in the *Arabidopsis lyrata *genomic sequence (Figure 1).

**Figure 3:**
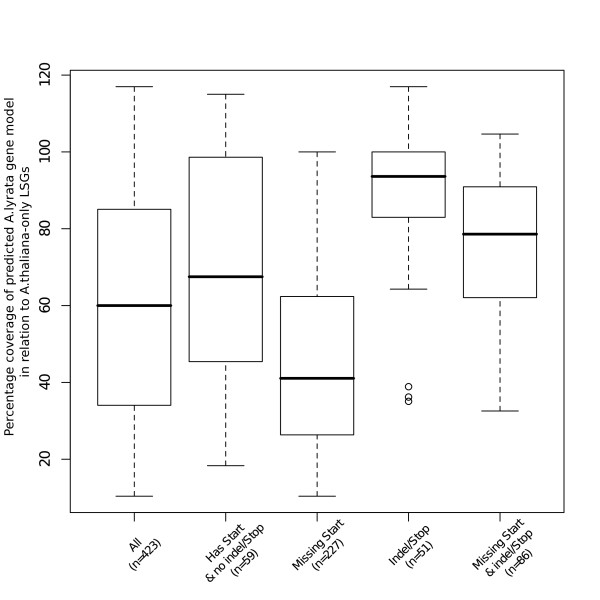
**Distribution of percentage coverage for LSG CDS vs. *Arabidopsis lyrata *intergenic region alignments**. Where percentage coverage is greater than 100% indicates a gap in the alignment in the LSG, indicating an indel in the LSG in *Arabidopsis thaliana*.

To determine the genomic context of *Arabidopsis thaliana*-only LSGs in the genome of its sister species *Arabidopsis lyrata*, LSGs with alignments to *Arabidopsis lyrata *scaffold sequences were split into four categories based on the aligned *Arabidopsis lyrata *sequence. Four categories can be identified for the LSG sequence in the *Arabidopsis lyrata *genome i.e. (1) an intact ORF in *Arabidopsis lyrata*; (2) a missing start codon in *Arabidopsis lyrata*, (3) indel(s) or internal stop codon(s) in *Arabidopsis lyrata *or (4) both a missing start codon and indel(s) or internal stop codons in *Arabidopsis lyrata*. Each of the four categories of these LSGs in *Arabidopsis lyrata *can be utilized to infer a model of evolution for the generation of a novel ORF at that locus (e.g. point mutations leading to either start codons or losing an internal stop codon). Using these four models for possible origins of *Arabidopsis thaliana*-only LSGs, the distribution of percentage coverage of the *Arabidopsis thaliana Arabidopsis lyrata *alignments for each category were then plotted (Figure 3). We note that these genomic contexts are also consistent with the loss of the ORF in *Arabidopsis lyrata*.

Using this approach we could determine that alignments where the *Arabidopsis lyrata *sequences lack a start codon and contain no indels or internal stop codons are the most frequent alignment category representing 53.66% of cases (Figure 3). These cases also represented the least coverage in terms of the alignment to the *Arabidopsis thaliana *LSG with a median percentage coverage of 41.07% ± 0.18. In contrast, the least frequent alignment category (i.e. evolutionary model) is where there are alignments with a start codon in the *Arabidopsis lyrata *sequences but containing either indels or internal stop codons, representing 12.06% of all cases. These also had the most sequence coverage recorded with a median percentage coverage of 93.62% ± 0.085 (Figure 3).

Sixty-three of the 423 LSGs with hits to *Arabidopsis lyrata *scaffold sequence have 95% or greater alignment coverage. Of these, 17 alignments have an intact ORF with no indel, no internal stop codon and have a start codon present in both species and possibly represent as-of-yet un-annotated ORFs in *Arabidopsis lyrata*, that are shared with *Arabidopsis thaliana*. The remaining alignments (with 95% or greater alignment coverage) display missing start codons and/or indels and/or internal stop codons and hence are not plausible/supported as protein coding genes in *Arabidopsis lyrata*.

Those *Arabidopsis thaliana*-only LSGs that display hits to intergenic regions in *Arabidopsis lyrata *(i.e. that do not form an intact ORFs in *Arabidopsis lyrata*) are consistent with evolutionary scenarios involving *de novo *origination and/or differential gene loss and retention. In the case of differential gene loss and retention scenarios (i.e. the gene has been lost in the *Arabidopsis lyrata *lineage rather than gained in *Arabiodopsis thaliana *lineage), whilst this would not explain the origin of these genes, the loss of the genes in all other species would at certainly explain why these genes are observed to be *Arabidopsis thaliana*-only. At this point, it is unclear whether these particular *Arabidopsis thaliana*-only LSGs have "died" in *Arabidopsis lyrata *or have been "born" in *Arabidopsis thaliana*. However, with whole genome sequencing of other closely related genomes which will become available shortly [44], this will be more easily elucidated for these particular genes.

To investigate further the possible origins of a subset of the *Arabidopsis thaliana*-only LSGs in relation to the *Arabidopsis lyrata *genome (and the *Arabidopsis cebennensis *genome) we generated novel sequence data for eight of the *Arabidopsis thaliana*-only LSGs (which display intergenic hits to *Arabidopsis lyrata*) across different accessions of *Arabidopsis thaliana *and accessions of the outgroup lineages *Arabidopsis lyrata *and *Arabidopsis cebennensis *[45]. Based on the GeneWise [46] modelling of the *Arabidopsis lyrata *draft sequence seven of the loci we focussed on for additional sequencing (AT1G62181.1, AT2G29654.1, AT2G36854.1, AT4G02465.1, AT4G38781.1, AT5G08220.1 and AT5G50361.1) were predicted to have interrupted ORFs in *Arabidopsis lyrata *and one that was predicted to have an intact ORF (AT2G46567.1), see Additional file 9 for summary of the accessions for which we could obtain amplicons and generate novel sequence data for each gene locus.

For each *de novo *sequence we generated, a gene model for that sequence was predicted using GeneWise and in each case the same indels, internal stop codons and/or missing start codons were validated as found in the *Arabidopsis lyrata *draft sequence. Furthermore, in some cases additional indels were found to be present in the genes from the *Arabidopsis lyrata *accessions we sequenced. In the case of AT2G46567.1, the intact ORF of AT2G46567.1 was found in all *Arabidopsis lyrata *accessions sequenced, see Additional file 10 for details of each alignment and predicted gene model. In particular, we identified that for LSGs AT4G38781.1 and AT5G53144.1 interrupted ORFs were present in the two distinct outgroup species i.e. *Arabidopsis lyrata *ssp. and *Arabidopsis cebennensis*, supporting a *de novo *origination of these two LSGs in the *Arabidopsis thaliana *lineage.

### Some lineage-specific genes are accession-specific within the *Arabidopsis thaliana *lineage

Lineage-specific genes must originate within specific populations for any given species. To identify accession-specific LSGs within *Arabidopsis thaliana *existing polymorphism data was mined to identify LSGs with interrupted ORFs (i.e. with either missing start codons or internal stop codons). Using the predicted SNPs from the Perlegen re-sequencing high-density oligonucleotide arrays data set [47]) and the Col-0 accession genetic background as a reference genome, from the overall set of 1761 nuclear genome LSGs identified in this study, sixty-one LSGs were identified having SNPs that result in an internal stop codon (ISC) while 13 LSGs had SNPs that result in a missing start codon (MSC). In each of these cases, this was observed for at least one of the nineteen different accessions of *Arabidopsis thaliana *tested using the Perlegen data (with an overlap of one between the ISC and MSC data sets). It should be noted that we only used SNPs predicted via both model-based and machine learning methods supported at a 2% false discovery rate (i.e. the MBML2 dataset) see Clark *et al *[47] for details. For our defined subset of *Arabidopsis thaliana*-only LSGs, thirty-seven of these LSGs are polymorphic for ISCs and/or MSCs (twenty-nine and eight respectively) across different accessions (Table 1). For the *Arabidopsis thaliana*-only LSGs containing an ISC or MSC in the *Arabidopsis thaliana *accessions analysed, the *Arabidopsis lyrata *intergenic alignments were also searched for SNPs causing either ISC or MSC at the sites identified in the *Arabidopsis thaliana *accessions. This allowed some initial insight into whether the ISC or MSC were restricted to the *Arabidopsis thaliana *lineage or whether they could also be found in the *Arabidopsis lyrata *lineage.

**Table 1:** Polymorphic LSGs resulting in interruption of the open reading frame.

LSG type	SNP type	Total	Gene model support	Locus support
Brassicaceae	ISC	32	12	22

*Arabidopsis thaliana*	ISC	29	8	18

Brassicaceae	MSC	5	3	3

*Arabidopsis thaliana*	MSC	8	1	5

Brassicaceae = Brassicaceae specific LSGs. *Arabidopsis thaliana *= *Arabidopsis thaliana*-only LSGs. SNPs resulting in internal stop codons = ISC. SNPs resulting in missing start codons = MSC. Support for expression at the locus provided by overlapping EST, cDNA or microarray probeset. Support for gene model expression provided by an EST or cDNA consistent with the of gene model (as listed by TAIR).

**Figure 4:**
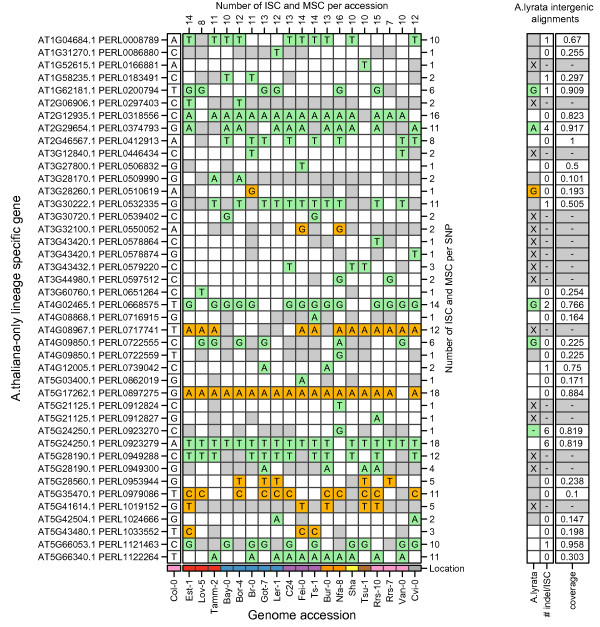
**Distribution of SNPs causing interruptions to the ORFs of LSGs in various *Arabidopsis thaliana *accessions**. Predicted SNPs predicted by Perlegen re-sequencing data sets that cause the interruption of an LSG ORF. Left hand axis: lists the gene model and the SNP name. Left hand column: Lists the reference nucleotide found in the Columbia accession (Col-0). Main body of table: SNPs causing a missing of a start codon (MSC) are coloured orange. SNPs causing an internal stop codon (ISC) are coloured green. Missing data (i.e. when the nucleotide is undetermined at that position) coloured gray. Only SNPs causing ISC or MSC are annotated. Bottom axis: lists the accessions tested, they are divided by broad geographical distinctions; i.e. red = Northern Europe, blue = Central Europe, purple = Mediterranean, orange = British Isles, yellow = Central Asia, brown = Japan, pink = North America and gray = Cape Verde Islands. Right hand columns: First column represents the SNP data at the position for the intergenic alignment between the LSGs and intergenic regions in *Arabidopsis lyrata*. SNP types marked the same as the main table with the addition of an "X" representing those instances were no alignment was identified in *Arabidopsis lyrata*. The second column represent the total number of ISC and indels found in the aligned *Arabidopsis lyrata *sequence. The final column represents the proportion of the LSG that is covered by the *Arabidopsis lyrata *alignment.

A total of 42 SNPs resulting in an ISC or MSC were identified in the 37 *Arabidopsis thaliana*-only LSGs displaying polymorphism across accessions (Figure 4). For those 37 genes, 24 had identifiable alignments in *Arabidopsis lyrata *intergenic regions, (Figure 4). The number of accessions containing a particular SNP resulting in an ISC or MSC ranges from 18 to one. In five cases in *Arabidopsis lyrata *the SNP causing the ISC or MSC is also present, while in a single case there is a deletion in *Arabidopsis lyrata *at the polymorphic site (Figure 4). In nine cases the *Arabidopsis lyrata *alignment does not cover the region of the *Arabidopsis thaliana *ISC/MSC SNP, while for the remaining 12 cases the *Arabidopsis lyrata *sequence does not have a SNP that causes an ISC or MSC at that site. However other ISC or indels were present in the *Arabidopsis lyrata *sequence for several of the alignments (Figure 4).

The use of sequence data from outgroup species allows inference of gene-loss vs. gene-birth scenarios for LSGs. If a SNP causing an ISC or MSC is conserved in *Arabidopsis lyrata *(across different accessions) it could indicate a gene birth event within the *Arabidopsis thaliana *lineage. Alternatively, if the SNP causing an ISC or MSC is not conserved in *Arabidopsis lyrata *and is only present in one or two *Arabidopsis thaliana *accessions then a gene loss event in the *Arabidopsis thaliana *lineage is the most parsimonious explanation. However, when using only one *Arabidopsis lyrata *sequence (i.e. the reference genome) it is unclear whether *Arabidopsis lyrata *is as polymorphic at that site as is *Arabidopsis thaliana*.

To validate predicted SNPs (from the Perlegen data and the *Arabidopsis lyrata *reference genome) and to check for the extent of conservation of polymorphisms in *Arabidopsis lyrata *we generated *de novo *DNA sequence data across a range of accessions of both *Arabidopsis thaliana *and *Arabidopsis lyrata *for four of the 37 *Arabidopsis thaliana*-only LSGs which displayed polymorphism across accessions based on the Perlegen data (namely AT1G62181.1, AT2G29654.1, AT2G46567.1 and AT4G02465.1) (Additional file 9). Note that the *Arabidopsis lyrata *sequences were also used to confirm and compare to the draft genome sequence data available for *Arabidopsis lyrata*. For each of these four genes, our sequencing data indicated that there were no polymorphisms in the *Arabidopsis lyrata *sequences (from each accession of *Arabidopsis lyrata *we sequenced) at the sites highlighted on Figure 4. However, as we did not obtain PCR amplicons for every gene in every *Arabidopsis lyrata *accession, these were only tested across one to seven additional accessions (see Additional file 10 for predicted gene models and alignments of reference genome ORFs with ORFs from our *de novo *sequencing) and therefore SNPs in other accessions not sequenced may possibly exist.

For all the *Arabidopsis thaliana *accessions in which we resequenced selected LSG loci, all Perlegen predicted SNPs (or lack-thereof) were fully validated with the following exception; AT2G46567.1 was not predicted by the Perlegen data to have a SNP causing an ISC in the accession Bur-0, however this SNP was identfied in our sequencing of that locus in Bur-0. Interestingly, this LSG locus has an intact ORF across seven different *Arabidopsis lyrata petraea *accessions yet is polymorphic in *Arabidopsis thaliana *(i.e. has an internal stop codon in Bay-0, Bur-0, Cot-7 and Cvi-0) therefore our data supports a gene loss rather than a gene gain scenario occurring for this particular locus within the *Arabidopsis thaliana *lineage, see Additional file 10 for the alignments of DNA and protein sequences for this locus.

There is no obvious geographical distribution or clustering for the ISC or MSC SNPs and the number of ISC/MSC causing SNPs ranged from 7 to 15 for any individual accession (Figure 4). The *Arabidopsis thaliana*-only LSGs, which are polymorphic for ISCs and MSCs, are the best candidates for identifying accession-specific LSGs within the *Arabidopsis thaliana *lineage, particularly those with expression support. A total of nine *Arabidopsis thaliana*-only LSGs, polymorphic for ISC or MSC, have expression support (EST, cDNA) consistent with the gene model (AT1G58235.1, AT2G46567.1, AT3G30720.1, AT3G43420.1, AT3G43432.1, AT4G12005.1, AT5G24250.1, AT5G43480.1, AT5G66053.1) with a further 14 with additional expression support (overlapping EST) for the locus (Table 1). Twenty-eight of the *Arabidopsis thaliana*-only LSGs that are polymorphic for ISCs/MSCs and have expression support are annotated as proteins of unknown function. In contrast, AT3G30720.1 (QUA-QUINE STARCH/QQS) is annotated as a protein involved in formation of starch (GO term: starch biosynthetic process) [48].

To further investigate LSGs not identified as containing a SNP in the Perlegen (MBML2) data set we sequenced five (AT1G61165.1, AT3G30160.1, AT4G31960.1, AT5G53144.1 and AT557567.1) *Arabidopsis thaliana*-only LSGs across several accessions (Additional file 9) to identify novel SNPs resulting in MSC or ISC. AT1G61165.1 was identified as accession specific with a MSC in the BAY-0 and CVI-0 accessions in addition to a MSC in *Arabidopsis lyrata*. The LSGs AT3G30160.1, AT4G31960.1 and AT557567.1 were found to have fully conserved ORFs across all *Arabidopsis thaliana *accessions for which we could obtain sequence data. The LSG AT5G53144.1 ORF was also conserved across all *Arabidopsis thaliana *accessions tested, but had a missing start codon in the *Arabidopsis lyrata *genome (Additional file 10).

### Lineage-specific genes have higher extent of tissue specificity and lower expression levels

To gain insights into to possible functions of LSGs and how they evolve, we next examined the expression patterns of LSGs in *Arabidopsis thaliana *as signature expression patterns may give possible indications of tissue, stage or stimulus specific-functions. For example, high tissue-specificity (of expression) has been highlighted as a characteristic associated with LSGs in rice, *Drosophila *and primates [7,9,23,49], as has stress response in rice and and *Hydra *[4,9,50]. Furthermore, there are LSGs in our dataset that are functionally annotated as having roles in biotic and abiotic stress response. For instance, biotic stress related LSG genes identified included three novel *NIM1*-interacting genes *NIMIN-1-related*, *NIMIN-2 *and *NIMIN-3 *[51]. Also, genetic data indicates that the LSG *ECS1 *(while not a defence gene itself) is linked to a locus influencing resistance to *Xanthomonas campestris pv. campestri *(Xcc750) [52]. The *PROPEP *precursor genes (LSGs) encode for peptides that induce the over-expression of *PDF1.2 *(encoding defensin) [53,54]. Other LSGs with potential stress roles include genes encoding for metallothionein proteins (MTA1, MT1B, MT1C) [55], Early Responsive to Dehydration 11 (ERD11) [56] and Induced by Phosphate Starvation1 (IPS1) [56]. Finally, Late Embryogenesis Activated (LEA) proteins M7 and M10 are also identified as LSGs expressed during embryogenesis and involved in the acquisition of desiccation tolerance [57].

In addition to stress response a role in reproduction has been highlighted for LSGs, in particular in *Drosophila *where LSGs have been shown to display a bias towards expression patterns in the testes [23,49]. A number of the functionally annotated LSGs in *Arabidopsis thaliana *also have roles in reproduction or seed development either demonstrated by experimental evidence, or inferred by the presence of conserved domains of motifs. For instance, the LSG *EMB2743 *is essential for embryonic development, while *ECA1 *is a gametogenesis related family LSG expressed in seeds [58]. In addition, 11 (ten genes plus one pseudogene) of a 12-member family of maternally expressed genes (MEG) are identified as LSGs. MPSS data indicates that four (of the 11 LSG MEGs) display tissue specific expression AT1G10717.1 and AT1G10745.1 are seed specific and AT2G29790.1 and AT1G10747.1 specific to "reproductive tissues" [58]. Finally, AT4G20420.1 and AT5G44540.1 are tapetum-specific protein-related, evidence provided by InterProScan (IPR00989).

**Figure 5:**
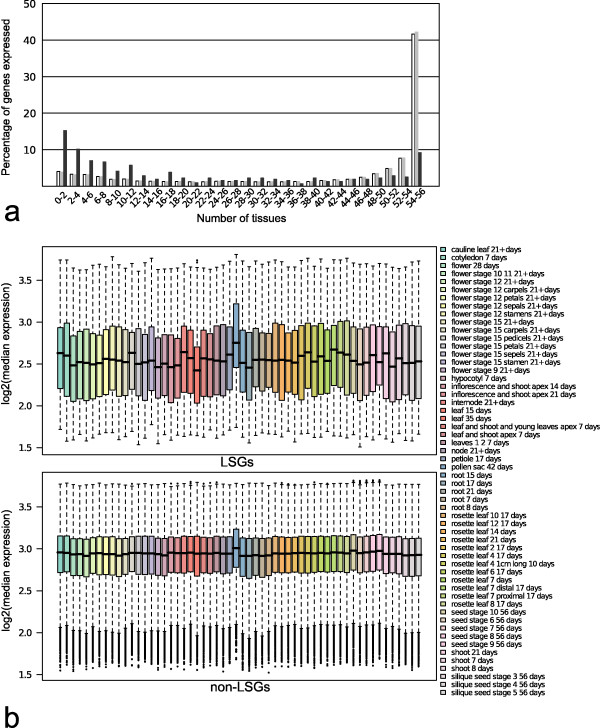
**Tissue expression patterns in LSGs and non-LSGs**. a) Distribution of the number of tissues/developmental-stage each gene called as present (expressed) in the AtGenExpress developmental series microarray experiment (see methods). White bars represent all representative gene models tested. Light gray bars represent non-LSGs. Dark gray bars represent LSGs. b) Distribution of log2 median expression of genes called as present for each tissue/developmental stage in the AtGenExpress developmental series microarray experiment.

To test whether LSGs in *Arabidopsis thaliana *display any distinct signature expression patterns, the expression patterns of 497 LSGs (with unique probesets on the affymetrix Ath1 microarray) were identified using the AtGenExpress developmental and stress series array datasets [59]. The expression patterns investigated were; (a) tissue specificity, (b) overall expression level, and (c) stress response. Tissue and developmental specificity was defined as the number of tissues and/or developmental stages that a gene was expressed using a gene expression present or absent call (i.e. present = expressed, absent = not expressed). LSGs and non-LSGs were compared by the percentage of each group that was expressed in *n *number of tissues. LSGs display greater tissue/developmental stage specificity compared to non-LSGs. The median number of tissues/developmental stages non-LSGs are expressed is 51 ± 18.5 compared to 4 ± 11.0 for LSGs. 52.72% of LSGs are expressed in four or fewer tissues/developmental stages, in comparison only 1.27% of non-LSGs are expressed in four or less tissues/developmental stages (Figure 5.a).

The expression value distributions for LSGs and non-LSGs were calculated using the median normalized expression for each gene and expression distributions for LSGs and non-LSGs were then compared for those genes called as present (i.e. expressed) for each tissue/developmental stage. For each tissue type LSGs have a lower median expression when compared to non-LSGs expressed in the same tissue type. The median non-LSG expression across tissues ranges from 2.9158 ± 0.2386 (root 17 days) to 3.0084 ± 0.228 (pollen 42 days). In contrast, the median LSG expression ranges from 2.4251 ± 0.3353 (leaf 35 days) to 2.7519 ± 0.3764 (pollen 42 days) (Figure 5.b).

### LSGs are enriched for gene expression responsiveness to abiotic stress conditions

Finally, to identify LSGs responsive to different stimuli, differentially expressed LSGs were identified using the AtGenExpress expression data series for abiotic stress, pathogen infection, growth condition treatments, chemical treatments and hormone treatments [59-62]. The abiotic stress conditions tested included: cold, drought, genotoxic, heat, osmotic, oxidative, salt UV-B and wounding. Biotic stresses included; bacterial (LPS, HrpZ, Flg22) and oomycete-(NPP1) derived elicitors, *Botrytis cinerea *infection, *Erysiphe orontii *infection, *Phytophthora infestans *treated, response to virulent, avirulent, type III-secretion system deficient and non-host bacteria and half leaf *Pseudomonas *treatment. The full list of treatment contrasts is provided (Additional file 11).

From the 497 LSG with unique probesets on the Ath1 affymetrix microarray a total of 130 LSGs are up-regulated and 103 down-regulated with an intersection of 96 (across all treatments), Additional file 12 provides a summary of all the differentially expressed LSGs for each condition tested, for both up- and down-regulated LSGs. Detailed results (including tissue types and time points) of the stress responsive LSGs are also provided in Additional files 13 &14. To test whether LSGs were enriched for stress response in any of the treatments the total number of differentially expressed LSGs and non-LSGs for each individual treatment, tissue and time point was found and hypergeometric tests performed.

For abiotic stresses LSGs are enriched in up-regulated genes for the following treatments: cold stress root tissue (time points: 3 and 12 hours), drought stress root tissue (time points: 0.25, 0.5 and one hour), genotoxic stress root tissue (time points: 0.5, 1, 2, 3, 6, 12, 24 hours), heat stress root tissue (time points: 1, 3, 4 6 and 12 hours), osmotic stress root tissue (time points: 0.5, 6, 12, 24 hours), oxidative stress root tissue one hour), UV-B stress root tissue (time points: 0.25, 0.5, 1, 6, 24) and shoot tissue (time points: 0.5, 1 and 24 hours). Finally, LSGs were enriched for up-regulated genes in roots exposed to wounding stress (time points: 0.5, 1, 6 and 24 hours). LSGs are also enriched for down-regulated genes in the following: cold stress root tissue (time points: one hour), genotoxic stress shoot tissue (time points: 6 hours) and heat stress root and shoot tissue (time point: 0.25 hours).

For biotic stresses, LSGs are enriched in up-regulated genes in the following pathogen treatments and time points; Flg22 enriched at one hour, *Erysiphe orontii *(mildew) infection enriched at 72 hrs. From the type III-secretion treatments avirulent *Pseudomonas *one at 24 hrs and the mock treatment at 24 hrs LSGs were also enriched in the up-regulated genes. LSGs are enriched for down-regulated genes in the mock treatment of the type III secretion treatment also at 24 hrs. No enrichment for LSGs was found in any of the growth condition, hormone or chemical treatments.

**Figure 6:**
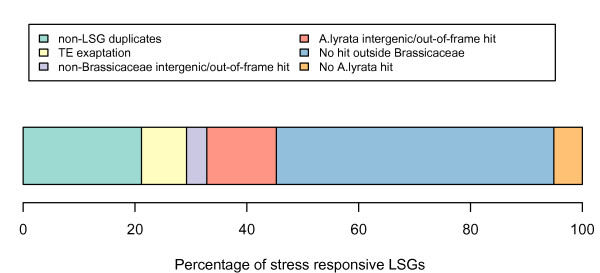
**Summary of the origins of stress responsive LSGs**.

In comparison to the evolutionary origins of LSGs in general, the stress responsive genes have a larger proportion of LSGs with unknown origins, with 30.48% and 49.64% for all LSGs and stress responsive LSGs respectively. In contrast, the stress responsive LSGs have a smaller proportion of LSGs with *Arabidopsis lyrata *out-of-frame or intergenic hits, 29.85% for all LSGs and 12.41% for stress responsive LSGs (Figure 6). The increased proportion of Brassicaceae-specific LSGs without identifiable origins could be suggestive of an increased rate of evolution at some time during the evolution of these genes, and indeed the stress responsive LSGs do display increased *d*_*N*_/*d*_*S *_values as observed in LSGs generally (Additional file 3). Furthermore, in contrast to the broader set of Brassicaceae LSGs, the lower incidence of stress responsive *Arabidopsis thaliana*-only LSGs suggests that a greater proportion of Brassicaceae-only stress responsive LSGs have been established before the divergence between *Arabidopsis thaliana *and *Arabidopsis lyrata*.

## Discussion

### Organelle Genomes display contrasting LSG content

This study demonstrates that the *Arabidopsis thaliana *mitochondrial genome has a much higher relative proportion of LSGs than found on any nuclear chromosome. In contrast, the *Arabidopsis thaliana *chloroplast genome contains no Brassicaceae restricted genes. These results likely highlight different evolutionary pressures on the essential energy producing molecular machinery of plants, and reflect the different mechanisms for generation of genetic novelty that occurs in each of these two organelles. Both organellar genomes have slower sequence evolutionary rates than the nuclear genome (i.e. low rates of point mutations) within plants [63]. The mitochondrial genome (i.e. chondriome) is the slowest evolving genome in land plants at the DNA sequence level. Within-population sequence divergence at silent sites in the chondriome is ~0.05 times lower that of the nuclear genome whereas the mutational rate of chloroplast genome (i.e. plastome) is >10 times higher than that of the chondriome [63]. In contrast to DNA sequence evolution via mutations, plant mitochondrial genomes are structurally dynamic whereby they can generate recombinational novelty, whereas the chloroplast genome is structurally highly conserved even in basal algal species such as *Mesostigma viride *[64,65]. Novel chimeric genes therefore can arise in plant mitochondria through the combined forces of frequent duplication and inversion in addition to the uptake of foreign DNA [64] and this likely explains the high proportion of LSGs observed in the chondriome of *Arabidopsis thaliana*. In contrast, the plastome is structurally conserved with intact gene clusters, and gene order, indicating low recombination rates. The plastome also has a much lower proportion of non-coding DNA (approximately one third of that found in the chrondriome [63]) indicating no or little uptake of foreign DNA. Therefore, it would appear that structural dynamism rather than mutational rate is more important for constructing novel open reading frames in the mitochondrial organellar genome of *Arabidopsis thaliana*. Our results indicate that LSGs occur at a much higher rate in the mitochondrial genome of *Arabidopsis thaliana *relative to its chloroplast genome, and this is consistent with the structural dynamism of mitochondrial genome evolution in plants.

### Duplicated genes provide the raw genomic material for LSG evolution

In this study several evolutionary mechanisms that could generate LSGs were considered, including duplication of non-LSGs, transposon exaptation and potential *de novo *origination. Our results determined that these mechanisms could account for the generation of the majority of novel LSGs in the *Arabidopsis thaliana *genome. However, it should be noted that the evidence for *de novo *origination for some genes is also consistent with differential gene loss and retention between *Arabidopsis thaliana *and *Arabidopsis lyrata*.

Duplication of non-LSGs has also been identified as an origination mechanism for LSGs in rice, primates and *Drosophila *[6,7,9]. A widely considered model proposed for gene duplication followed by divergence states that the redundancy of function experienced by one copy of the gene after a duplication event can lead to relaxed constraints allowing an increased rate of evolution that can in turn facilitate neo-functionalization given the right environmental conditions [17,66]. Indeed, elevated rates of evolution are observed in LSGs across many species including rice [9], *Arabidopsis thaliana *[28] and our results presented in Additional file 3, primates [7] and *Drosophila *[7,14], the *E. coli-Salmonella enterica *clade in bacteria [13], and also fungal species from the Ascomycota phylum [6,13]. All of these studies support the duplication and divergence model for the origination of some LSGs. Interestingly, in our study we also demonstrate that the duplicated parental non-LSGs are also experiencing elvated rates of evolution compared to most genes in the genome, suggesting that LSGs arise from duplication of genes that are already fast evolving themselves.

Our study also highlights that LSGs generated from genic duplications are under-represented in segmentally duplicated blocks of paralogs, and we could only trace one LSG to any of three whole genome duplication (WGD) events of *Arabidopsis thaliana *genome history. Indeed, this single LSG traced back to a WGD event is linked to the *At*-α event. To put this finding in perspective, approximately 25% of all *Arabidopsis thaliana *genes are found to have a *At*-α duplicate [33]. Hence, our findings suggest that the generation of LSGs typically occurs mostly on the single gene (locus) scale, with evidence of both local tandem duplications and distal duplications (transposition-duplications) being observed with higher frequency. Single gene transposition-duplications are incompletely understood [33], although, it is known that certain mobile elements such as *mutator-like *elements [67] and helitrons [39] can transpose gene fragments around the genome. Interestingly, gene families (such as defensins and thionins) that have high tandem and transposition-duplication frequencies are also over-represented in the LSG dataset [33]. Notably, for the small number of LSGs we have identified that are consistent with a retrotransposition event, none of these LSGs have been identified as retrogenes in previous studies [18,19]. The most likely reason for these LSGs not being identified as retrogenes in previous studies is because the LSGs recognized here consist of partial matches of highly divergent genes that would not have been identified in the other studies.

We also demonstrate duplication of non-LSGs that generate LSGs that code for peptides in a different reading frame, in some cases with the LSG duplicate inverted or only a partial duplicate identifiable. In some cases, chimeric gene structures forming LSGs can contain partial duplicates of several non-LSGs or contain exapted TE DNA or DNA that overlaps with an existing non-LSG CDS (as identified in this study for 32 LSGs). In the context of evolutionary scenarios for the generation of novel LSGs, it is worth considering that stop codons are AT rich and coding DNA has a higher GC content than intergenic DNA. Consequently, the emergence of novel ORFs (i.e. LSGs) may be easier/more probable from a duplicate of an existing gene than from intergenic DNA (even in the cases where the reading frame of the novel gene differs from that of the parental gene).

The high number of *Arabidopsis thaliana*-only LSGs we have identified in this study provides further evidence of the plasticity of the *Arabidopsis thaliana *genome. The subset of *Arabidopsis thaliana*-only LSGs we have identified that display alignments to intergenic regions of the *Arabidopsis lyrata *genome, represent the best current candidates for identification of *de novo *origination of LSGs within the *Arabidopsis thaliana *lineage (using the four models of evolution we posited as possible leading to novel *Arabidopsis*-only LSGs (Figure 3). Our analysis indicates that lack of a start codon in the orthologous genomic locus in *Arabidopsis lyrata *is the predominant reason for the LSGs investigated being limited to the *Arabidopsis thaliana *lineage. To distinguish between *de novo *origination of an LSG in the *Arabidopsis thaliana *lineage vs differential gene loss of an LSG between the *Arabidopsis thaliana *lineage and close relative outgroups (*Arabidopsis lyrata, Arabidopsis cebennensis*) we sequenced a subset of *Arabidopsis*-only LSGs across different accessions of *Arabidopsis thaliana, Arabidopsis lyrata *and *Arabidopsis cebennensis*. For a number of these genes, frameshift indels in the *Arabidopsis lyrata *or *Arabidopsis cebennensis *ablated the LSG ORF and could hence account for these LSGs being specific to *Arabidopsis thaliana *and likely of *de novo *origin in the *Arabidopsis thaliana *lineage (e.g. At4g38781.1 and At5g53144.1). In addition to validating the sequence fidelity of the existing Perlegen and *Arabidopsis lyrata *genome sequence data, our DNA sequencing data also allowed us to identify a range of LSGs which are *Arabidopsis thalian*a accession-specific and hence excellent candidates for *de novo *origins of an LSG within the *Arabidopsis thaliana *species genepool. To our knowledge, LSGs AT2G29654.1, AT1G62181.1 and AT1G61165.1 represent the first LSGs, which have been found to be arising (i.e. gene gain) in some *Arabidopsis thaliana *intra-species lineages (i.e. accessions) but not in others.

### Evolutionary constraints may select against conserved overlapping CDS

LSGs could potentially arise within existing CDS of non-LSGs, either on the + or - DNA strands. While approximately ~1000 pairs of genes in the entire *Arabidopsis thaliana *overlap with antisense transcripts [68], the occurrence of two CDS sequences overlapping in the overall *Arabidopsis thaliana *genome is a relatively uncommon event. Our study identified only 105 gene models in total in the *Arabidopsis thaliana *genome that have an overlap that spanned the CDS of both genes. Approximately a third of these cases involve a LSG (including twenty-one cases where an LSG and non-LSG overlap) giving additional insight to the origins of novel LSGs. Two possible evolutionary scenarios are; (1) either the sequence that the LSG consists of was present in ancestral species and via neutral processes gained an ORF overlapping an existing gene, or (2) the LSG ORF was lost in all other species and only maintained in Brassicaceae, with scenario (1) being the most parsimonious explanation. Similar to what is observed in *Arabidopsis thaliana*, vertebrates have differing sets of overlapping genes (i.e. non-conserved) across different species, subsequently leading to high proportion of overlapping pairs of genes containing an LSG [69]. However, if such LSG overlaps are constantly occurring throughout evolution why have so few been conserved across different lineages, leading to the current situation where each lineage has different overlapping CDS? One possible explanation could be that, over long evolutionary periods, genes with overlapping CDS may be selected against due to the selective pressures of maintaining two ORFs in the same DNA sequence without a loss of fitness to the established non-LSG CDS.

### Tissue specificity of LSGs, low levels of LSG expression over developmental stages, and stress related LSGs all support a waiting model of evolution

The genesis of a new protein-coding gene depends on two fundamental features (regardless of the mechanism from which it was derived) i.e. the presence of an ORF and the presence of a transcription start site (TSS). Our study demonstrates that LSGs in *Arabidopsis thaliana *display a higher extent of tissue-specific expression, when compared to non-LSGs. This suggests a lower incidence of regulatory motifs in the upstream promoter regions of LSGs than for conserved genes, which is consistent with previous observations in primates [7].

Whilst the presence of an ORF and TSS are necessary requirements for the establishment of a new protein-coding gene, such criteria provide no guarantee that the new gene (or protein product) will have any functionality. A gene can be considered to be essential if a knockout results in lethality or infertility (under normal growth conditions). In contrast, a non-essential gene can be considered more functionally dispensable and/or redundant [70]. As essential genes provide key roles in the life cycle of an organism they require stable and highly regulated expression, while non-essential genes tend to exhibit a greater extent of tissue-specific functionality and responses to environmental changes [9]. Any new LSG generated should typically be initially non-essential, unless its genesis generates a gain-of-function essential phenotype in rare instances. However, non-essential novel genes can gain functionality over time by being selected for as environmental conditions change - this scenario describes the "waiting model" of evolution [17]. The waiting model describes the genesis of novel genes that can experience increased rates of evolution due to an initial lack of functionality. It could also be expected that such novel genes should initially be lowly expressed, thereby reducing any potential dosage toxicity to the cell resulting from the novel gene product. The subsequent acquisition of function could arise due to such genes providing selective advantages when environmental conditions change. The findings of this study of LSGs in *Arabidopsis thaliana *fit well with the waiting model of evolution of LSGs, as LSGs exhibit both low expression over developmental stages and there is significant enrichment for LSGs in response to abiotic stresses. Our findings are also consistent with the stress response of LSGs others have found in rice [9] and *Hydra *[4,9,50]. Overall, our findings lend support for the waiting model to explain the evolutionary development of LSGs in *Arabidopsis thaliana*.

A number of UV-B and heat stress LSGs have been highlighted previously in *Arabidopsis thaliana *[27]. Additionally, proteins of unknown function have been observed to be responsive to oxidative stress [71], which likely includes a number of the LSGs identified in our study. Our study also provides a comprehensive analysis of LSGs expression responses to a wide range of stress conditions. Looking at the stress response of *Arabidopsis thaliana *LSGs in detail, our study finds not only some stress responsive LSGs, but strikingly observes that LSGs are enriched for genes that are responsive to a range of abiotic stress stimuli, specifically in root tissues (although enrichment is also observed in shoot tissues under UV-B stress). In addition, LSGs are also enriched when leaves of *Arabidopsis thaliana *are infected with some important plant pathogens. The investigation of functions of abiotic and biotic responsive LSGs is currently the subject of follow up work to this study. Interestingly, some subsets of LSGs display similar expression responses to a range of different stresses (abiotic, biotic or both) that could suggest some core or fundamental stress response feature of these LSGs not previously identified. This would be consistent with the initial transcriptional stress reaction of *Arabidopsis thaliana *genome leading to expression of a set of core stress responsive genes [62].

## Conclusions

The inherent ability of species to generate novel gene content over evolutionary time is becoming ever more evident as each newly sequenced genome identifies an additional cohort of LSGs. It has been suggested that an ability to generate expressed non-essential transcripts provides organisms with a reservoir of genomic raw material that allows an organism to adapt quickly to any environment changes it may encounter [72]. Indeed, this is particularly important in plant species as plants (or their cells) cannot easily move away from stress challenges, and therefore must depend upon genetic, molecular and biochemical mechanisms to provide robustness and evolutionary endurance. In *Arabidopsis thaliana*, our study demonstrates that the genome has a number of evolutionary mechanisms that can be harnessed to generate novel gene content, with some mechanisms of origination more prevalent than others in the *Arabidopsis thaliana *lineage. Furthermore, we have identified a core stress response as a striking feature of a subset of LSGs in *Arabidopsis thaliana *that fits well with the waiting model of evolution, and also with the reliance of plant species on flexible molecular solutions to dealing with environmental stresses. The enrichment for stress responsiveness (across a multitude of stress conditions) observed for LSGs provides insights to their possible functions, and supports our working hypothesis that LSGs are important for environmental adaptation strategies.

## Methods

### Data retrieval

Peptide, genomic and CDS sequences of all representative gene models plus the gene and transposon GFF file from the *Arabidopsis thaliana *genome were downloaded from The Arabidopsis Information Resource (TAIR v8) [73]. Gene models with EST and/or cDNA evidence were identified using TAIR. The peptide, genomic and CDS sequences from the rice genome were downloaded from the Rice Annotation Project v6 [74]. The poplar peptide, genomic and transcript sequences were downloaded from Joint Genome Initiative (JGI) v1.1 [75]. Papaya genome peptide, genomic and transcript sequences were downloaded from The Hawaii Papaya Genome Project v1 [76]. Grape peptide, genomic and transcript sequences were downloaded from Grape Genome Browser v1 [77]. Sorghum genomic and transcript sequences were downloaded from the JGI [78,79]. *Arabidopsis lyrata *peptide, genomic and transcript sequences (v1) were download from the JGI [80]. Copies of NCBI databases nr (non redundant protein), nt (core nucleotide) and EST (expression sequence tag) at the Irish Centre for High-End Computing (ICHEC) [81], were used to perform NCBI BLAST searches and InterPro scans. Brassicaceae genus names were obtained from the NCBI Taxonomy website [82].

### Identifying Non-overlapping BLAST hits in *Arabidopsis thaliana*

Each LSG was BLASTP searched against a database of *Arabidopsis thaliana *peptides. For each significant hit (e < 0.01) all overlapping HSPs were concatenated and those concatenated HSPs covering less than 10% of the LSG were discarded. The percentage coverage of non-overlapping HSPs was then calculated. This was repeated using BLASTN and a database of *Arabidopsis thaliana *CDS. The percentage coverage of the BLASTP and BLASTN results for each LSG was compared. Those with greater CDS coverage than peptide coverage were identified as having additional out-of-frame alignment segments. LSGs with non-overlapping hits to different genes were identified, as having multiple-parent-gene origins (listed as chimeric, see results). LSGs with non-LSGs alignments with intron-exon boundaries within five base pairs of each other were listed as consistent with segmental duplication. Alignments where the LSGs crossed an intron-exon boundary in the non-LSG but (the LSG) had no intron in that region were listed as consistent with retrotransposition.

### Identifying overlapping features

Overlapping gene models, and gene models overlapping transposable elements, were identified using their genomic coordinates (Arabidopsis GFF file). Repeatmasker [83], was used to identify transposable elements in the mitochondrial genome.

### Reciprocal BLAST hits to non-Brassicaceae plant species CDS of intergenic regions

BLASTN searches were performed for all LSGs against the CDS or transcript sequences of rice, sorghum, grape, poplar and papaya. For each LSG with a significant hit (e < 0.01) covering at least 10% of the LSG, the top hit was BLASTN searched against the *Arabidopsis thaliana *CDS sequences. Reciprocal top hits were identified. For those LSGs with no significant CDS hit to a non-Brassicaceae CDS, BLASTN searches were performed using the genomic sequences (i.e. pseudo-chromosomes and/or scaffold sequences). For those LSGs with a significant hit (i.e. e < 0.01 and covering at least 10% of the LSG) the genomic location of the HSP in the non-Brassicaceae species was identified and the corresponding sequence (plus 100 bp of up- and down-stream sequence) was extracted from the pseudo-chromosome or scaffold sequence. This sequence was then BLAST searched against the *Arabidopsis thaliana *CDS and reciprocal hits identified.

### Identifying and classifying intergenic *Arabidopsis lyrata *hits

BLASTN searches for all *Arabidopsis thaliana*-only LSGs against *Arabidopsis lyrata *transcripts were performed. All those LSGs with a significant hit (e < 0.01 and percentage coverage at least 10%) were filtered. The remaining LSGs were BLASTN searched against the genomic sequence of *Arabidopsis lyrata *(i.e. scaffold sequences). For all LSGs with a significant hit, all intergenic *Arabidopsis lyrata *hits on the same scaffold sequence as the top hit and within 4000 base pairs of another hit were concatenated. This involved taking the two most extreme coordinates of all the hits (i.e. the most upstream and downstream coordinates) on the scaffold that met the criteria of being within 4000 bp of another significant hit. These extended sequences were then used with the *Arabidopsis thaliana *LSG peptides sequence to predict the peptide model of the *Arabidopsis lyrata *extended hit sequence, using GeneWise [46]. Predicted gene models covering less than 10% of the LSG were discarded. Internal stop codons, missing start codons and indels were identified for each predicted model.

### Identifying accession-specific LSGs

#### Data retrieval

TAIR8 polymorphism GFF file and Perlegen array re-sequencing SNP predictions [47], were downloaded from the TAIR website.

#### Identifying polymorphisms causing internal stop codons or missing start codon in LSGs

All polymorphisms found within the CDS of LSGs were identified using the TAIR8 polymorphism and TAIR8 genes and transposons GFF files. Only predicted SNPs in the MBML2 data set were considered (see Clark *et al *2007 [47]). For each accession the polymorphic sequence was translated and polymorphisms resulting in either a missing start codon of an internal stop codon were identified for each polymorphic LSG for each accession.

### Sequencing and alignments of LSGs in sister species

Different accessions from three *Arabidopsis sp. *were used; *Arabidopsis thaliana *accessions- N22614 (CVI-0), N22621 (CS22491), N22630 (Ag-0), N22633 (Bay-0), N22639 (Kas-1), N22643 (NOK-3), N22647 (TS-1), N22658 (OY-0), CS1642 (Ler-1), Got-7 (CS22283) and CS1028 (Bur-0) (obtained from Nottingham Arabidopsis Stock Centre); *Arabidopsis lyrata petreae *accessions- NT12b (8-3), Tannenberg T1a (15-1), random T14 (17-7), random T25 (13-4) and random spiterstulen (11-2) and *Arabidopsis cebennensis *(obtained from Prof. Karl Schmid, Institute of Plant Breeding, Seed Science and Population Genetics, University of Hohenheim, Germany). Genomic DNA was isolated from leaf tissue of each accession according to the CTAB method [86].

Genomic DNA from each accession was used for PCR amplification of eight ORF sequences using ORF specific primer sets (Additional file 12) designed from the flanking regions of each ORF. The lineage-specific ORFs that were targeted for PCR amplification were; AT1G61165.1, AT1G62181.1, AT2G29654.1, AT2G36854.1, AT2G46567.1, AT3G30160.1, AT4G02465.1, AT4G31960.1, AT4G38781.1, AT5G08220.1, AT5G50361.1, AT5G53144.1 and AT5G57567.1.

Each 50 ul PCR reaction contained 1× GoTaq buffer (Promega, USA), 2 mM MgCl_2_, 10 pmol of each primer, one unit of GoTaq DNA polymerase (Promega, USA), 2.5 μM of each dNTP, and ~25 ng of template genomic DNA. The PCR cycle was 2 min at 94°C, 35 cycles of [30 sec at 94°C, 45 sec at 56°C and 1 min 30 sec at 72°C], 5 min at 72°C, and a final holding temperature of 10°C. All PCR amplicons were confirmed as single bands on a 1% w/v agarose gel stained with ethidium bromide (0.5 ug/mL), prior to sequencing. Each PCR amplicon was sent for sequencing at GATC Biotech, London, UK. Forward and reverse sequences were trimmed and aligned to generate a consensus sequence for each ORF in each accession. Sequences were submitted to Genbank GSS library, see Additional file 15 for GSS IDs and Genbank accessions.

Gene models and peptide sequences of the sequenced regions were predicted using GeneWise. Multiple sequence alignments were performed on the peptide sequences using MUSCLE [84]. Those sequences that returned predicted models with indels were aligned separately to the Col-0 peptide sequence and the indels highlighted using GeneWise.

### Comparison of tissue expression LSGs vs. non-LSGs

#### Data retrieval

Microarray ATH1 CEL files from the AtGenExpress developmental series were downloaded from ArrayExpress and NASC databases [59-61].

#### Identifying absolute expression

The Wilcoxon signed rank-based gene expression presence/absence detection method implemented by MAS5calls method (part of the affy package in Bioconductor [85,86]) was used to give absolute calls for the AtGenExpress developmental series [87]. Probesets matching either none or several loci in the *Arabidopsis thaliana *genome were filtered. If a probeset was identified as present in any of the three biological replicates it was recorded as present for that tissue type/developmental stage. Number of tissues for each gene was expressed in was calculated and the results were split into two groups LSGs and non-LSGs compared and plotted.

#### Identifying tissue expression levels

The AtGenExpress developmental series was normalized as a single AffyBatch using RMA method in the affy package in Bioconductor. Probesets matching either none or several loci in the *Arabidopsis thaliana *genome were filtered out. Also, redundant probesets that represent the same locus several times were counted only once (using the probeset with the greatest interquartile range). The median expression value across the three replicates for each gene called as present, for each tissue type/developmental stage was then calculated and combined into a single expression matrix as previously described [88]. The results were split into LSGs and non-LSGs and the distribution of the expression values were plotted for each tissue/developmental stage.

### Identifying stress responsive LSGs by microarray differential expression analysis

#### Data Retrieval

Microarray ATH1 CEL files from the AtGenExpress abiotic, biotic, hormone, chemical stress, growth conditions series and controls were downloaded from ArrayExpress and NASC databases [60,61].

#### Differential expression analysis

For each comparison the data was normalized as a single AffyBatch using RMA method in the affy package in Bioconductor. Probesets matching either none or several loci in the *Arabidopsis thaliana *genome were filtered. Non-specific filtering of the remaining probesets was performed using the genefilter package. Differentially expressed genes were identified using the LIMMA package [89]. Adjusted p-values were calculated using FDR method [90]. Confidence thresholds of adjusted p-value < 0.01 and fold change > log2(1.5). Enrichment analysis for LSGs in up and down-regulated genes was performed using a hypergeometric test using a p-value < 0.05.

## Abbreviations

CDS: coding sequence; HSP: high-scoring segment pair; ISC: internal stop codon; MSC: missing stop codon; LSG: lineage-specific gene; ORF: open reading frame; SNP: single nucleotide polymorphism; TE: transposable element; TSS: transcription start site.

## Authors' contributions

MD designed and performed all the computational analysis. CK designed primers and CK and SHS isolated DNA and conducted the DNA sequencing. MD prepared the figures and data. MD and CS prepared the manuscript. CS oversaw the research programme and developed the investigative strategies with MD. All authors read and approved the final manuscript.

## Supplementary Material

Additional file 1 **Search schema to identify Brassicaceae LSGs**. Click here for file

Additional file 2 **The effect of using different E value cut-offs in BLAST**. "X" indicates the number of LSGs reported after the InterProScan, here the x axis (E-value cut off) is irrelevant. Click here for file

Additional file 3 **Supplementary information: Including genomic feature comparisons between LSGs and non-LSGs**. Click here for file

Additional file 4 **Table listing all LSGs their gene description and genomic coordinates**. Click here for file

Additional file 5 **LSGs with overlapping CDS with the CDS of a non-LSG**. Click here for file

Additional file 6 **Distributions of E-values for LSG non-LSG paralog BLASTP and BLASTN hits**. Click here for file

Additional file 7 **Details of all LSG non-LSG paralog BLASTP and BLASTN hits**. Click here for file

Additional file 8 **Percentage of each transposable element super-families contributing DNA to the CDS of LSGs and non-LSGs CDS**. Dark gray = percentage of TEs that contribute DNA to LSG CDS content, mid gray = percentage of TEs that contribute DNA to non-LSG CDS content, light gray = percentage of TEs that do not contribute any DNA to any gene model CDS. Click here for file

Additional file 9 **LSGs and accessions that were sequenced**. Click here for file

Additional file 10 **Alignments and gene models of sequenced LSGs in various accessions and sister species**. For the multiple sequence alignments: black = identical residues, blue = similar residues, red = other residues (i.e. non-matching). In addition, for the gene model alignments "!" Indicates indel and the number of nucleotides are displayed below. For ambiguous nucleotides; m = A or C, y = C or T and w = A or T and "x" = undetermined peptide. Click here for file

Additional file 11 **Stress conditions tested for differential expression of LSGs**. Click here for file

Additional file 12 **Summary of all stress responsive LSGs**. Red = up-regulated genes, blue = down-regulated genes. For the stress conditions listed across the bottom of each table; blue = abiotic, green = biotic, purple = growth conditions, yellow = hormone treatment and red = chemical treatment. Click here for file

Additional file 13 **Heatmaps indicating fold change of LSGs expressed under abiotic and biotic stress conditions**. Genes highlighted with a p-value indicate significant differential expression. Colour bars at the top of columns indicate an enrichment of LSGs differentially expressed: red = up-regulated, blue = down-regulated, yellow = LSGs are enriched for both up and down-regulated genes. Click here for file

Additional file 14 **Details of differentially expressed LSGs for growth condition, treatments, chemical treatments and hormone treatments**. Click here for file

Additional file 15 **Forward and reverse primers used fro sequencing and Genbank accessions of sequences**. Click here for file
