# Supplementary figures and images for: Evolutionary origins of Brassicaceae specific genes in Arabidopsis thaliana
Source: BMC Evol Biol. 2011 Feb 18;11:47. doi: 10.1186/1471-2148-11-47 (PMC3049755; doi:10.1186/1471-2148-11-47)

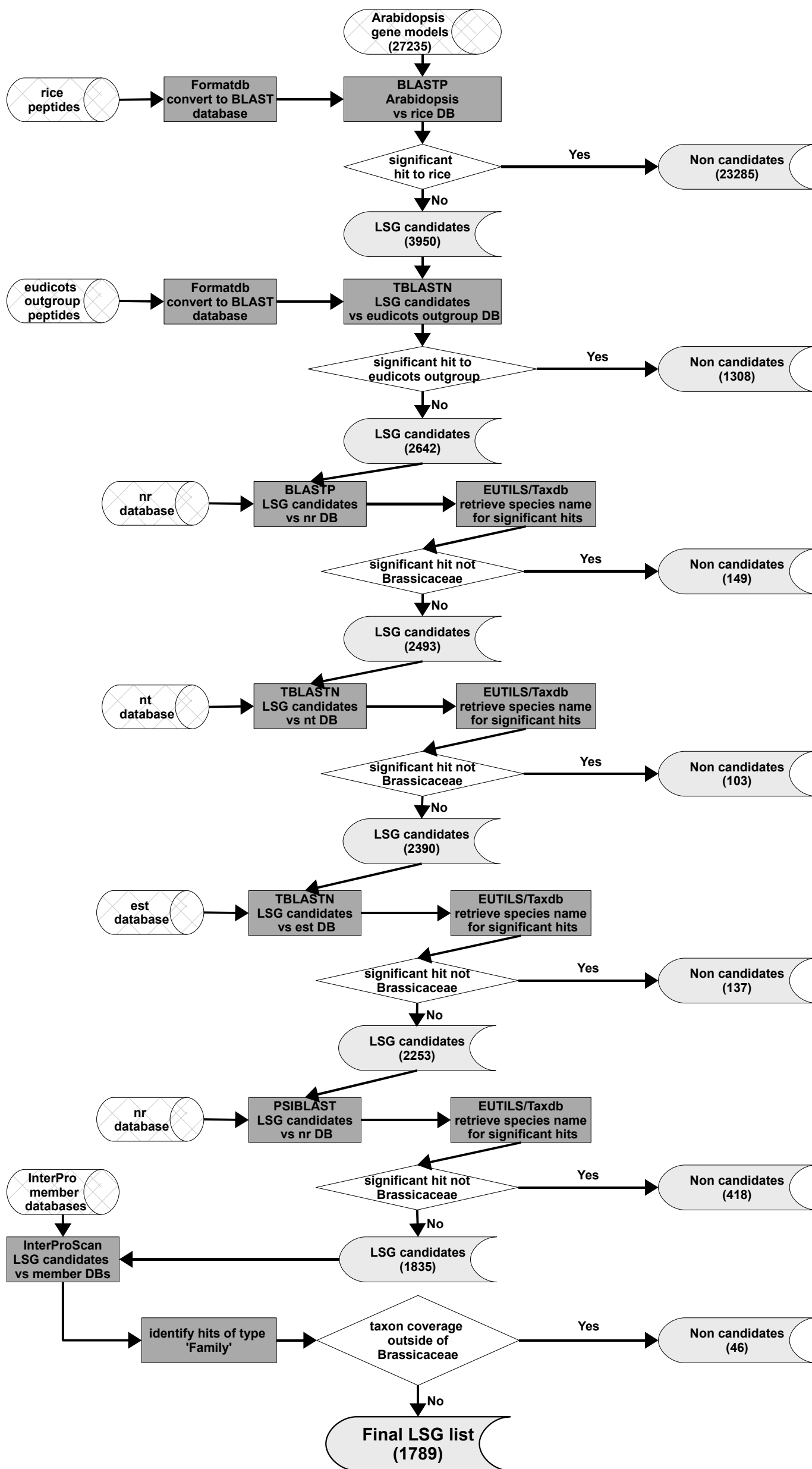

Supplement: Additional file 1 — Search schema to identify Brassicaceae LSGs. [file 1471-2148-11-47-S1.PDF]

### Number of LSGs reported using different E-value cut-offs

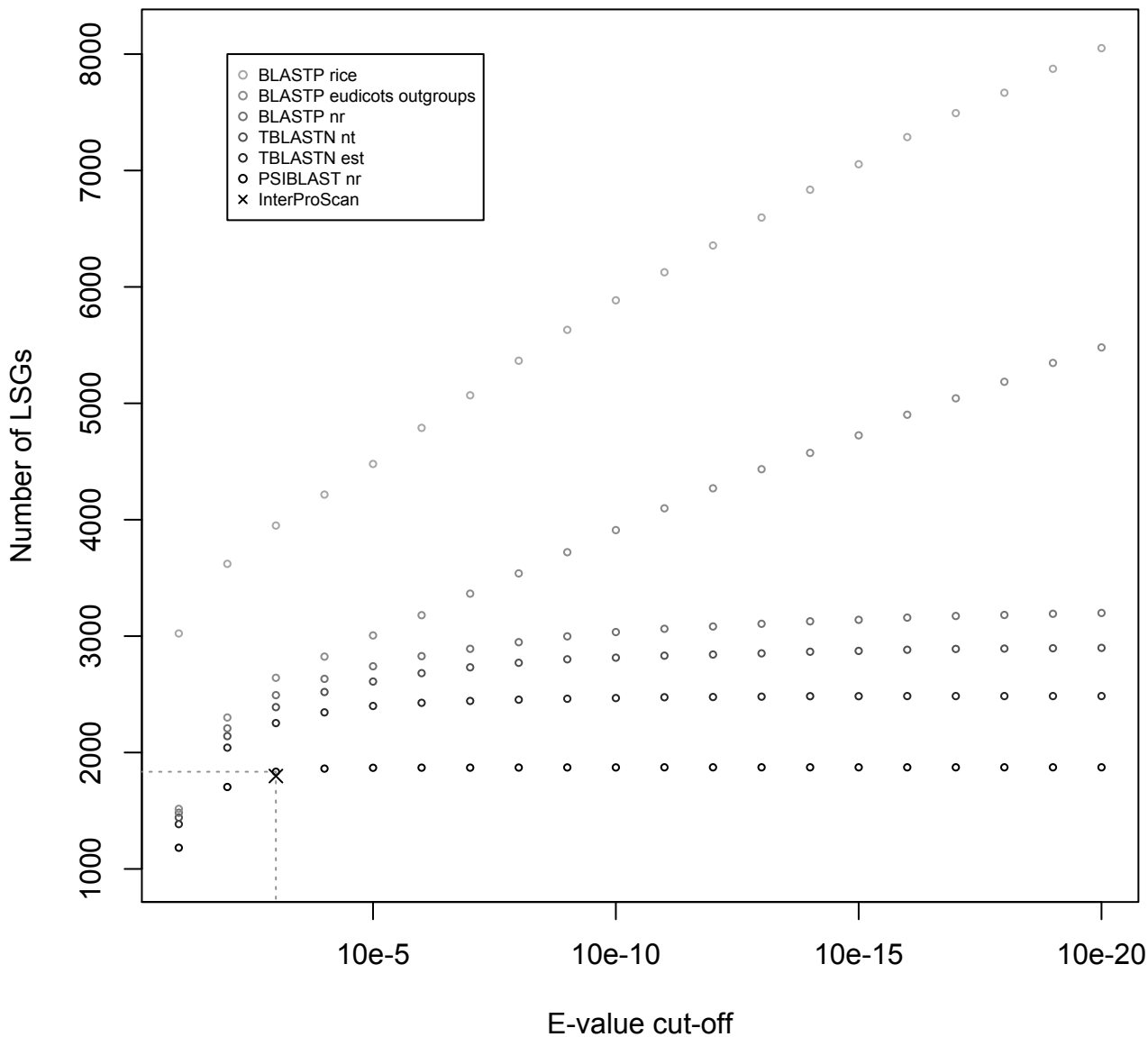

Supplement: Additional file 2 — The effect of using different E value cut-offs in BLAST. "X" indicates the number of LSGs reported after the InterProScan, here the x axis (E-value cut off) is irrelevant. [file 1471-2148-11-47-S2.PDF]

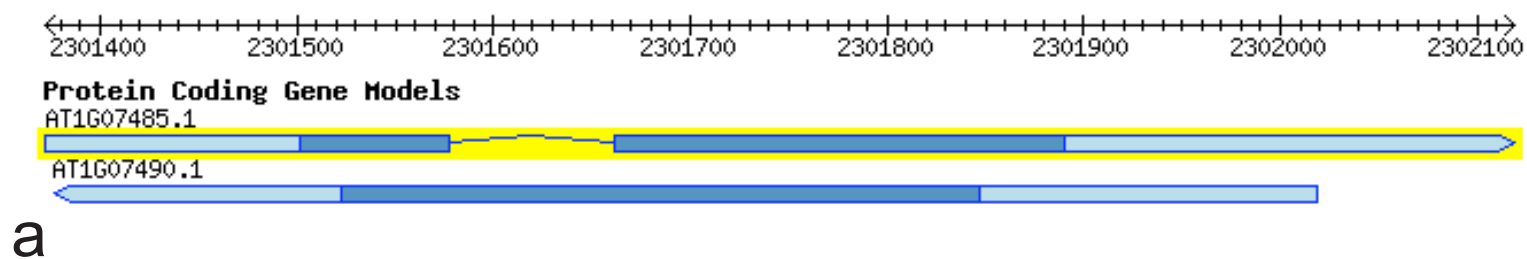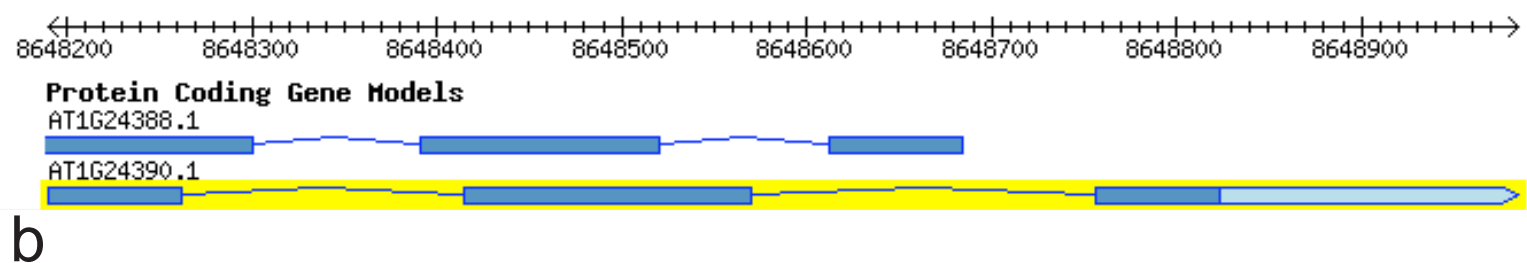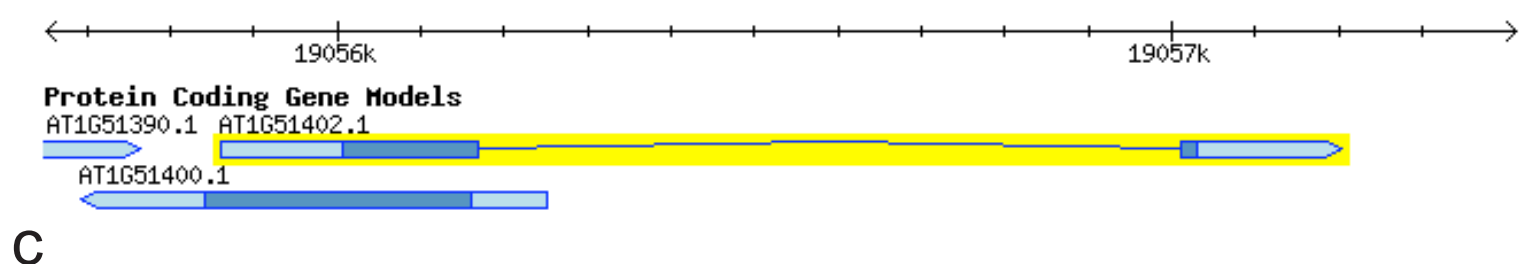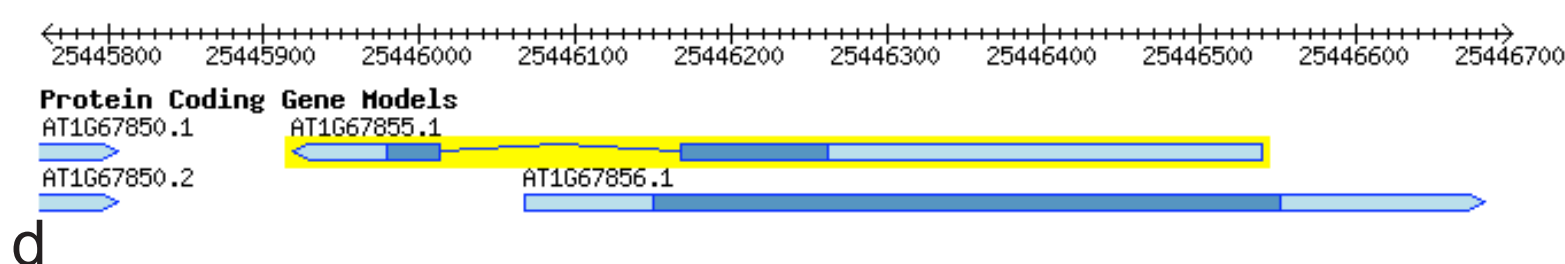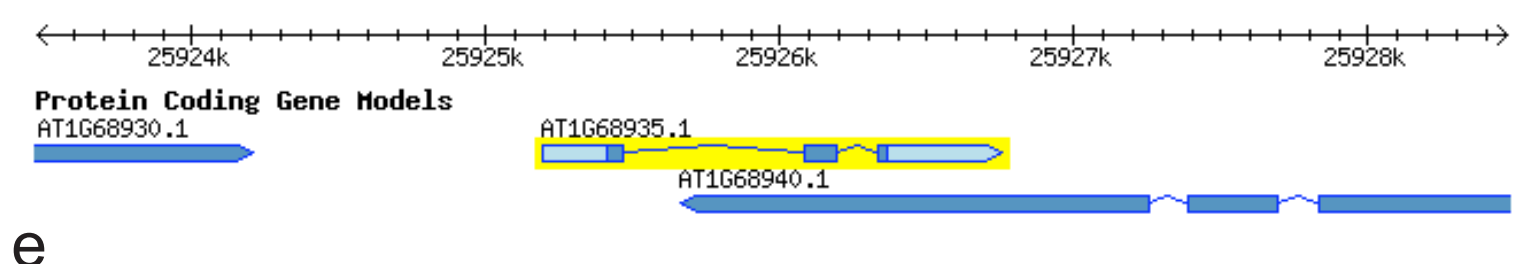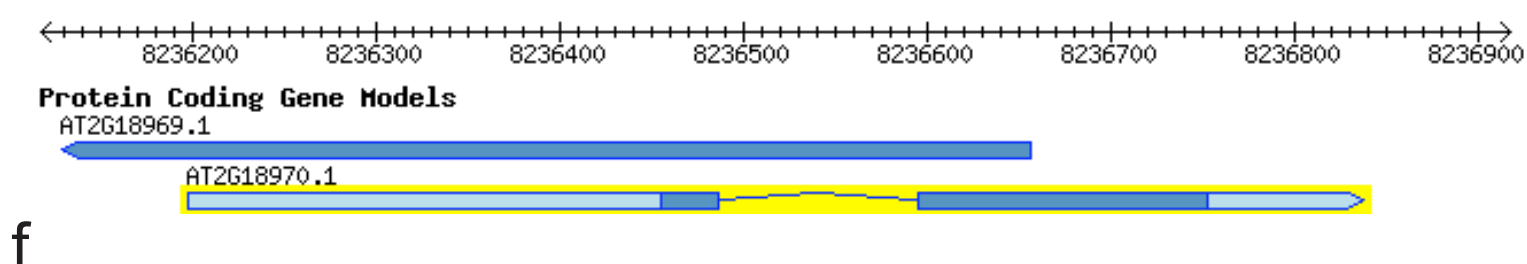

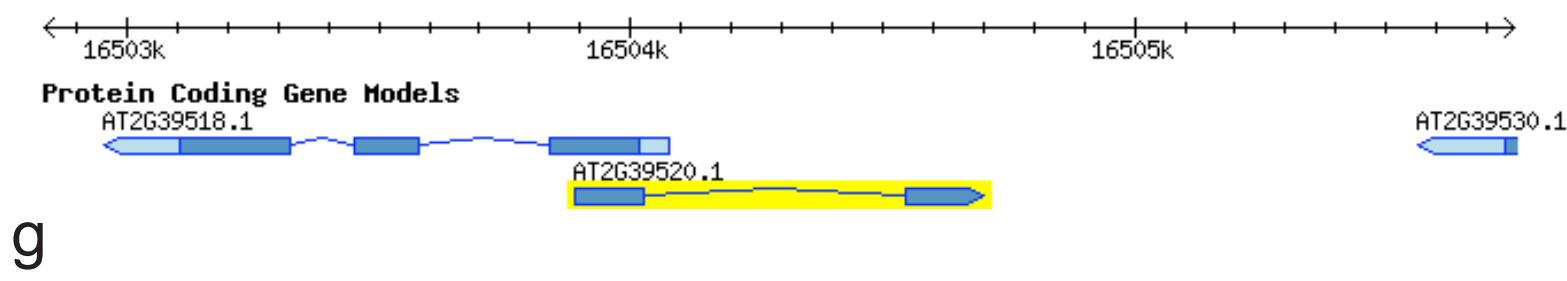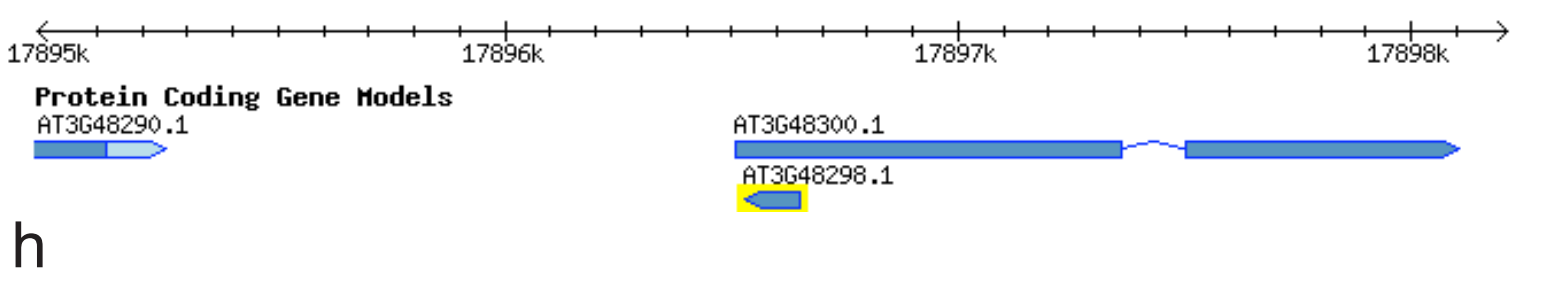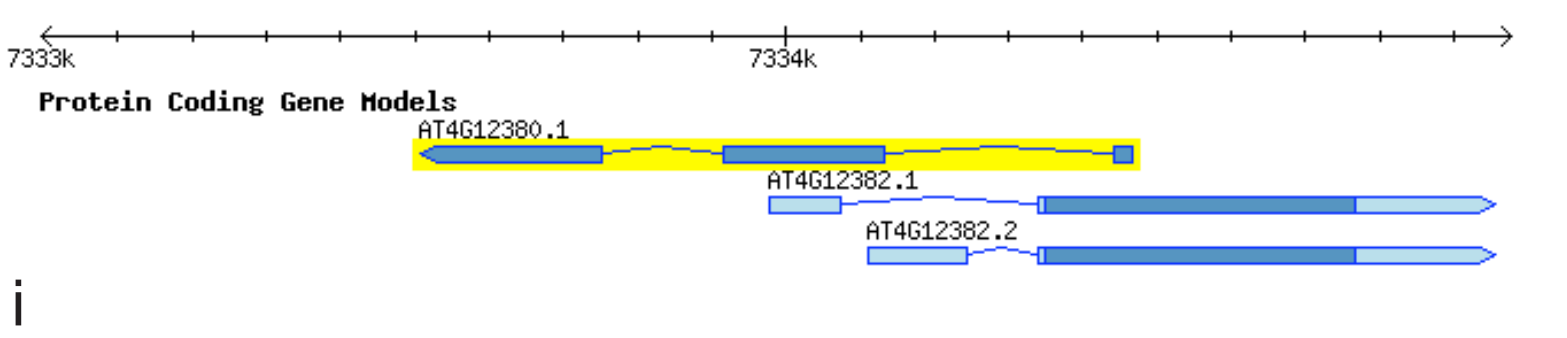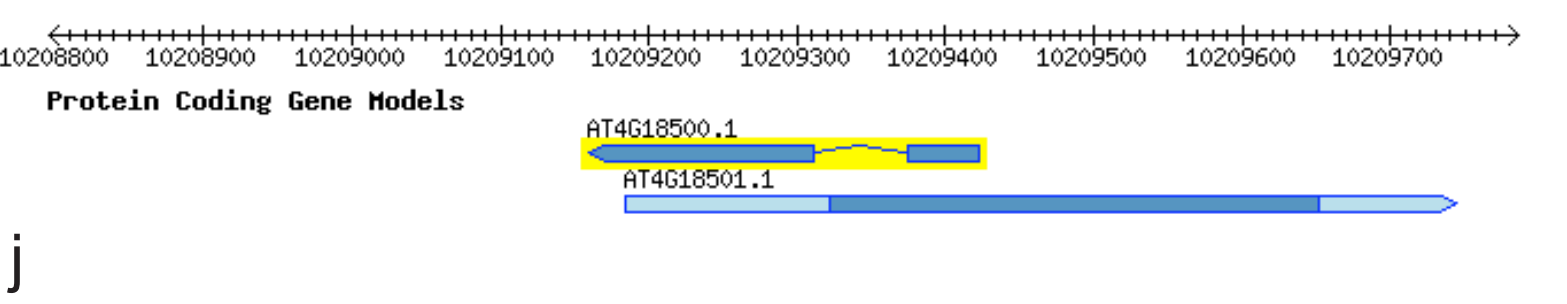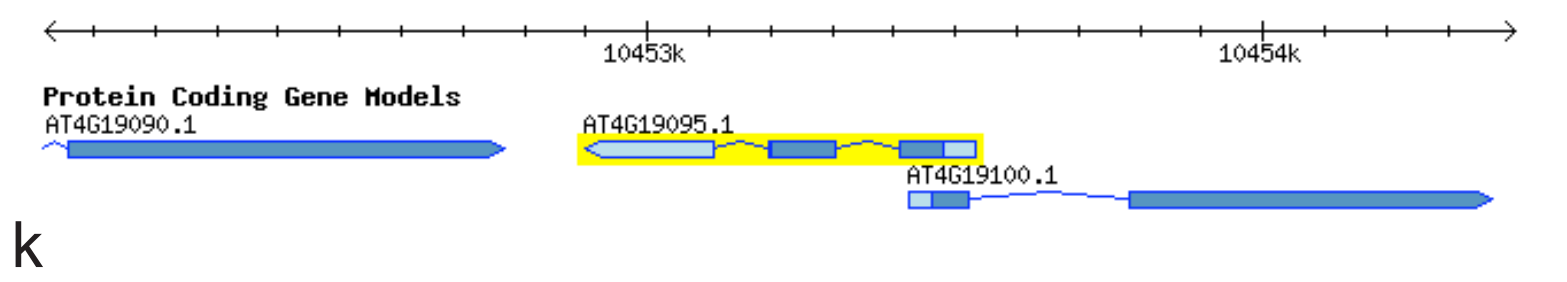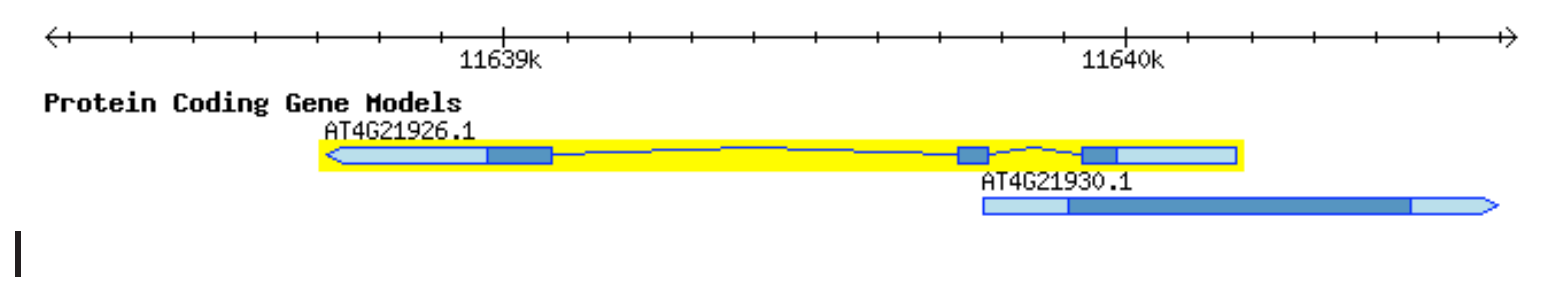

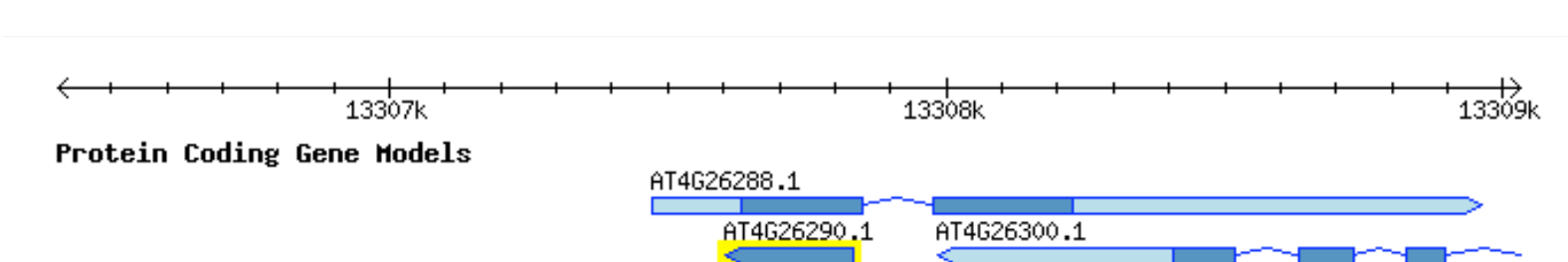

m

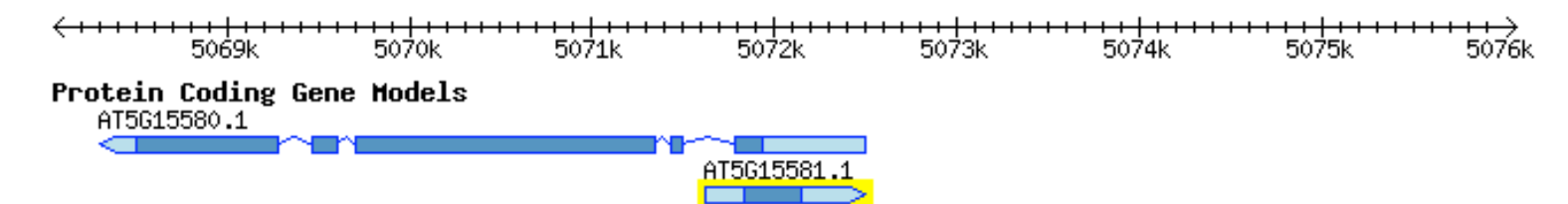

n

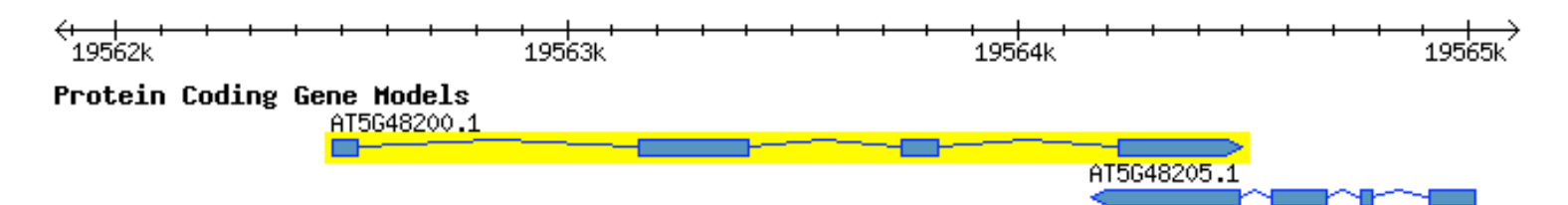

o

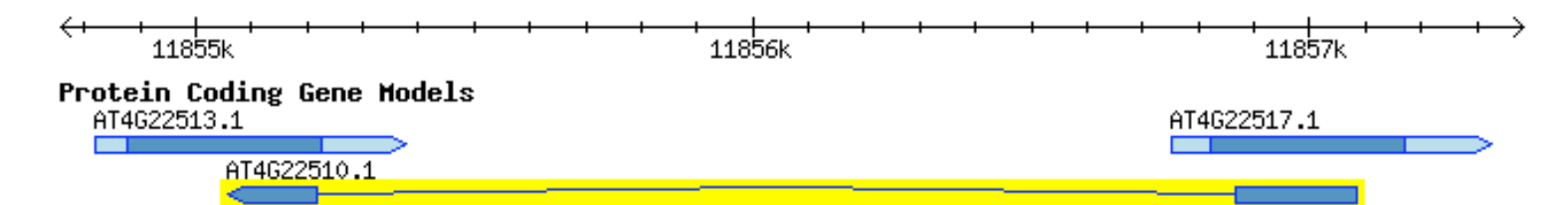

p

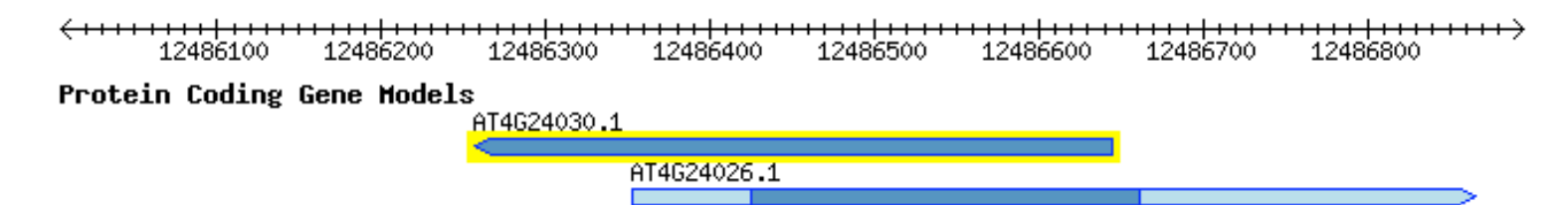

q

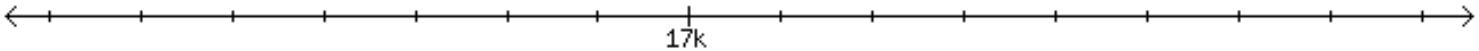

Protein Coding Gene Models

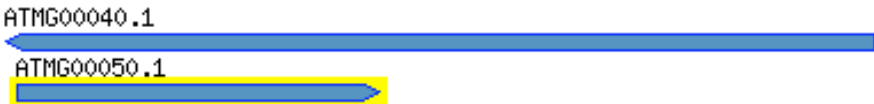

r

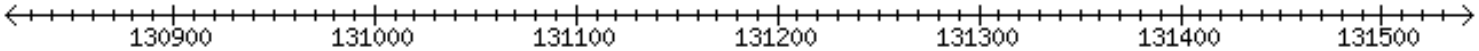

Protein Coding Gene Models

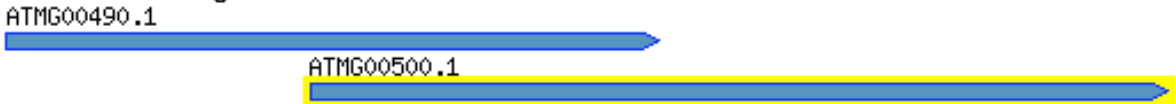

s

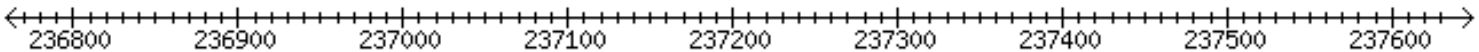

Protein Coding Gene Models

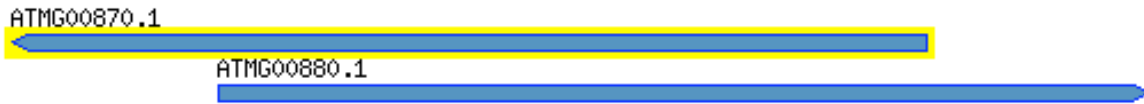

t

Supplement: Additional file 5 — LSGs with overlapping CDS with the CDS of a non-LSG. [file 1471-2148-11-47-S5.PDF]

# Percentage of TEs with exapted DNA contributing to CDS

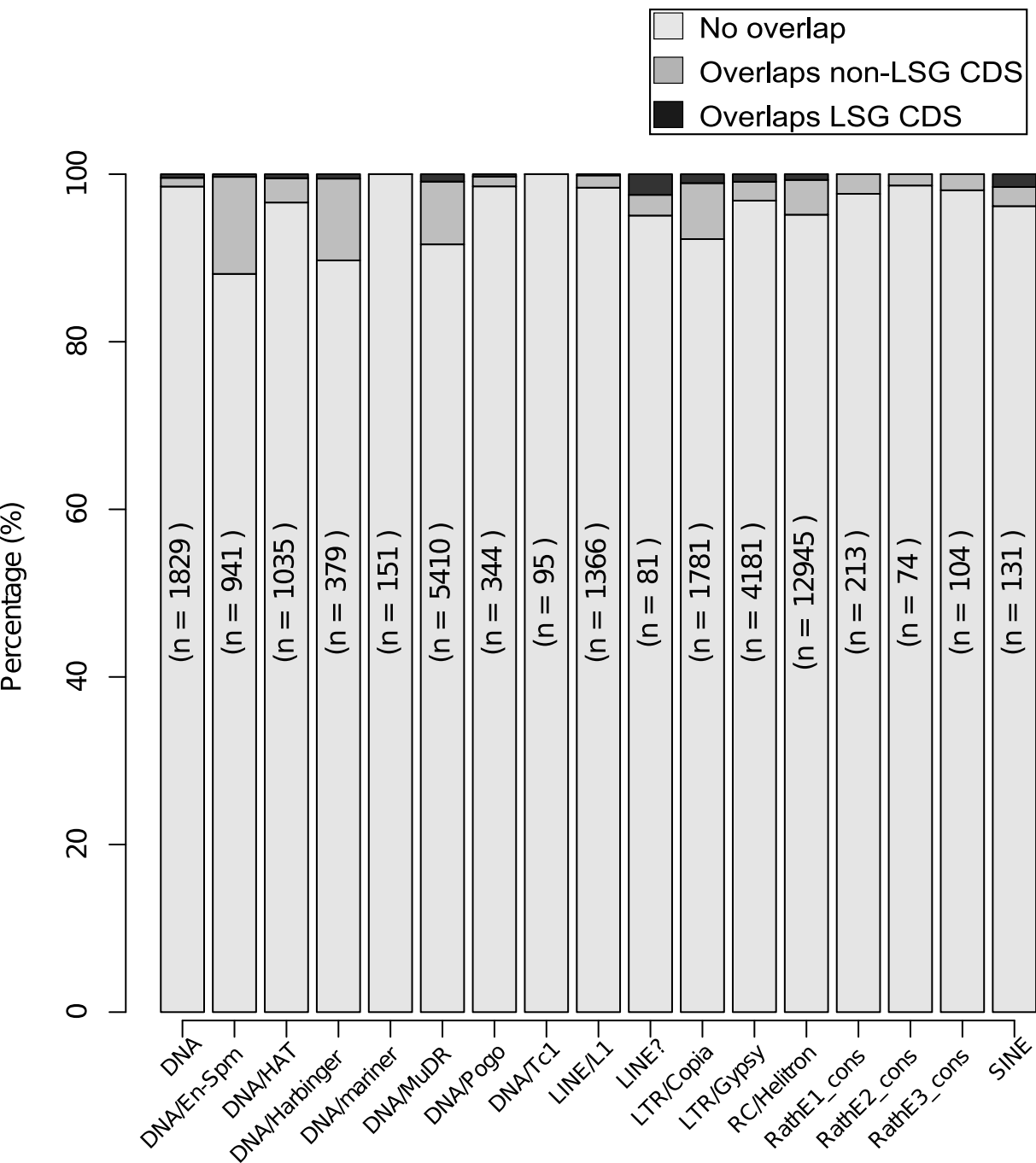

Supplement: Additional file 8 — Percentage of each transposable element super-families contributing DNA to the CDS of LSGs and non-LSGs CDS. Dark gray = percentage of TEs that contribute DNA to LSG CDS content, mid gray = percentage of TEs that contribute DNA to non-LSG CDS content, light gray = percentage of TEs that do not contribute any DNA to any gene model CDS. [file 1471-2148-11-47-S8.PDF]
