# Supplementary material for: Evolutionary origins of Brassicaceae specific genes in Arabidopsis thaliana
Source: BMC Evol Biol. 2011 Feb 18;11:47. doi: 10.1186/1471-2148-11-47 (PMC3049755; doi:10.1186/1471-2148-11-47)
Supplement: Additional file 3 — Supplementary information: Including genomic feature comparisons between LSGs and non-LSGs. [file 1471-2148-11-47-S3.DOC]

# Supplementary Information

## LSGs in *Arabidopsis thaliana* are poorly characterised

Many bioinformatics methods for assigning function to novel genes utilize sequence homology therefore assigning putative functions to LSGs is difficult when there is little or no identifiable sequence homology. This is reflected in the proportion of genes GO annotated [1] as unknown function or component. Approximately 47.79%, 88.41% and 97.60% of LSGs are GO annotated as unknown cellular component, unknown biological process or unknown molecular function respectively. In comparison the conserved genes tested, approximately 18.70%, 22.19% and 18.45% annotated as unknown cellular component, unknown biological process and unknown molecular function respectively. However, there are a number of LSGs that been researched and shown to have functions or otherwise categorized.

| **AGI annotation** | **Count** |
| --- | --- |
| Unknown protein | 1145 |
| Similar to unknown protein | 215 |
| Defensin-related/defensin-like (DEFL) | 117 |
| Encodes a Plant thionin family protein | 46 |
| Similar to another protein (any species) | 43 |
| Conserved peptide upstream open reading frame (CPuORF) | 30 |
| Hypothetical protein (28 mitochondrial) | 29 |
| SCR-Like | 18 |
| Glycine-rich | 17 |
| LCR Low-molecular-weight cysteine-rich | 15 |
| Maternally expressed gene MEG family protein (10 + 1 pseudo gene) | 11 |
| Expressed protein | 11 |
| Identical to Uncharacterized mitochondrial (LSG) protein | 9 |
| Contains domain PROKARLIPOPROTEIN PS51257 | 7 |
| Elicitor peptide precursor (PROPEP) | 6 |
| Encodes a Protease inhibitor seed storage LTP family protein | 3 |
| METALLOTHIONEIN (MT1A, MT1B, MT1C) | 3 |
| ARABINOGALACTAN (AGP) | 2 |
| CLAVATA3ESR-RELATED (CLE) | 2 |
| Cytochrome-c oxidase | 2 |
| Endopeptidase inhibitor | 2 |
| Hydroxyproline-rich glycoprotein family protein | 2 |
| NIM1-INTERACTING | 2 |
| Nucleic acid binding zinc ion binding | 2 |
| Proline-rich family protein | 2 |
| Tapetum-specific protein-related | 2 |
| Zinc knuckle CCHC-type family protein (related) | 2 |
| 3-5-exoribonuclease RNA binding | 1 |
| ATP binding aminoacyl-tRNA ligase nucleotide binding | 1 |
| Arabidopsis thaliana purine permease (ATPUP) | 1 |
| Calcium ion binding | 1 |
| cAMP-dependent protein kinase inhibitor-related | 1 |
| Contains domain Kazal-type serine protease inhibitors SSF100895 | 1 |
| Contains domain PRICHEXTENSN PR01217 | 1 |
| Contains domain PTHR10432SF61 PTHR10432SF61 contains domain PTHR10432 | 1 |
| Contains domain PTHR23172SF19 PTHR23172SF19 contains domain PTHR23172 | 1 |
| Contains domain Ubiquitin-like SSF54236 | 1 |
| Contains InterPro domain Carbohydratepuine kinase PfkB conserved site IPR002173 | 1 |
| Contains InterPro domain ENTHVHS IPR008942 | 1 |
| Contains InterPro domain Nucleic acid-binding OB-fold-like IPR016027 | 1 |
| Contains InterPro domain Protein of unknown function DUF321 IPR005529 | 1 |
| Contains InterPro domain Somatomedin B IPR001212 | 1 |
| Contains InterPro domain WD40 repeat-like IPR011046 | 1 |
| Early-responsive to dehydration protein-related ERD protein-related | 1 |
| ECA1 gametogenesis related family protein | 1 |
| ECS1 | 1 |
| EMB2743 EMBRYO DEFECTIVE 2743 | 1 |
| Embryo-specific protein-related | 1 |
| Emsy N terminus domain-containing protein ENT domain-containing protein | 1 |
| Encodes a cysteine-rich peptide CRP family protein | 1 |
| Glycineproline-rich protein | 1 |
| Glycine-rich cell wall protein-related | 1 |
| Hormone | 1 |
| Invertasepectin methylesterase inhibitor family protein | 1 |
| IPS1 INDUCED BY PHOSPHATE STARVATION1 | 1 |
| Lyase | 1 |
| M10 | 1 |
| M17 | 1 |
| Membrane protein | 1 |
| Methyltransferase | 1 |
| MIR162A | 1 |
| Nucleic acid binding nucleotide binding | 1 |
| PEP7 ELICITOR PEPTIDE 7 PRECURSOR | 1 |
| PLS POLARIS | 1 |
| Protein binding | 1 |
| Protein kinase C-related | 1 |
| RALF-LIKE (RALFL) | 1 |
| Steroid hormone receptor transcription factor | 1 |
| Systemic acquired resistance SAR regulator protein NIMIN-1-related | 1 |
| Ubiquitin-associated UBATS-N domain-containing protein | 1 |
| WRKY family transcription factor | 1 |

**Table 1: LSGs with AGI annotation**

## Short peptides is a feature of LSGs

If formed *de novo* from non-coding regions then short open reading frames would be an expected feature of LSGs. Similarly, if LSGs have arisen from partial gene duplication they would be shorter than their parent gene. To investigate the length of LSGs (n=1761) compared to non-LSGs (n=25265) the distribution of the length in amino acids was identified and compared for both sets. The distributions of peptide length significantly differ with LSGs much shorter with a median length 77 ± 30.5 compared to non-LSGs with a median length of 366 ±155. The distribution of peptide length is significantly different between LSGs and non-LSGs (Wilcoxon Rank Sum, p < 0.01). Furthermore, a protein sequence can be defined as a peptide if it has a molecular weight less than 10kDa [11]. 38.01% of LSGs fall into this category.

## Less introns is a feature of LSGs

LSGs can result from gene duplication or partial gene duplication. Gene duplications can occur due to unequal crossing over or via transposon activity. Duplication of genes via retro-transposition result in a copy of the gene without introns whereas unequal crossing over will result in a copy of the gene with the introns intact. LSGs may also result from de-novo exonization of intergenic regions and would likely to be simpler shorter more basic gene structures. To determine whether LSGs have less introns than non-LSGs the distribution of the number of introns was compared between nuclear LSGs (n=1761) and non-LSGs (n=25265). The median number of introns for LSGs is 0 ± 0.5 compared to median number introns for non-LSG genes of 3 ± 2.5. The distribution of the number of introns is significantly different between LSGs and non-LSGs (Wilcoxon Rank Sum, p < 0.01).

## Cysteine Rich LSGs

The largest family of cysteine rich peptides is the DEFL family, these DEFL peptides are characterised as having smaller size and high sequence diversity except for conserved cysteines often in the form of cysteine-stabilized alpha beta (CSαβ) motif and/or γ-core motif and the presence of a signal peptide [12]. Other cysteine rich proteins (CRP) identified within the LSG dataset include the thionin (n=46), defensin-like S-locus cysteine rich (SCR) (n=18), Low-molecular-weight cysteine-rich (LCR) (n=14), lipid transfer protein (LTP) (n=3), maternally expressed genes (MEG) (n= 11), RALF and RALF-like (n=2) and tapetum specific (n=2). [4].

## Lineage-specific genes display atypical GC content due to amino acid bias

Coding sequence tends to have a different GC content than non-coding regions. Mutation pressure can lead towards a higher AT content and can be considered as an indicator of relaxed constraints in the absence of purifying selection [31]. To examine the GC content of LSGs the distribution of overall GC and GC1, GC2 and GC3 content was determined for both LSGs and non-LSGs and then compared

In concordance with the findings of Lin *et al* (2010) [28] and analyses of LSin other species [5, 14], the median percentage GC content of the CDS is lower in LSGs (43.0% ± 0.0325) compared to non-LSGs (44.19% ± 0.0195)However, the analysis in this study of GC content across each codon reveals that the major difference observed in GC content occurs at GC1, **Figure 1.a**. The median difference of GC1content between LSGs and non-LSGs is 4.67% (i.e. LSG median GC1 content is 45.5% ± 5.0769 compared to non-LSGs 50.17% ± 2.9133). In contrast, the median difference in GC2 and GC3 content between LSGs and non-LSGs is 0.39% and 0.04% respectively. Differences in overall GC, GC1 and GC3 are statistically significant, whereas the difference in GC2 is not (Wilcoxon Rank Sum, p < 0.01).

The first base in a codon is non-synonymous in the majority of codons, only three amino acids are coded by codons that are synonymous at the first base, i.e. arginine (A or C), leucine (C or T) and Serine (A or T). Therefore the observed differences in GC1 likely indicate differences in amino acid usage between LSGs and non-LSGs. To compare the amino acid usage of LSGs and non-LSGs the position dependent start methionine was removed and amino acid frequencies were plotted against each other, **Figure 1.b**. The largest increase in frequency of an amino acid is found in cysteines that account for 1.8% of amino acids in non-LSGs and 3.% of amino acids in LSGs (a 72.97% increase), **Figure 1.b**.

Consistent with the amino acid bias observed, a significant proportion of LSGs are cysteine rich ORFs. For instance, this study identified 117 defensin-like and 46 thionin family proteins (both families cysteine rich). In addition, the 11 MEG genes and the two tapetum-specific genes are cysteine rich genes. Such cysteine rich genes were originally reported as hard to identify novel genes by Silverstein *et al* [32, 33] and more recently highlighted as LSGs by Lin *et al* [28].

The next highest increases in amino acid frequencies were observed for phenylalanine, which accounts for 4.29% and 4.9% of amino acids in non-LSGs and LSGs respectively an observed increase of 15.71%, and arginine which accounts for 5.3% and 6.06% of amino acids in non-LSGs and LSGs respectively, an increase of 12.51%, **Figure 1.b**. All other amino acids with increased frequency in LSG represent less than a ten percent increase for each amino acid. Alanines have the largest decrease in frequency (16.9%) in LSGs followed by glutamic acid, glutamine and aspartic acid (12.75%, 13.98% and 14.25% respectively), **Figure 1.b**. The remaining amino acids with decreased frequency in LSGs represent less than ten percent decrease in each.

**
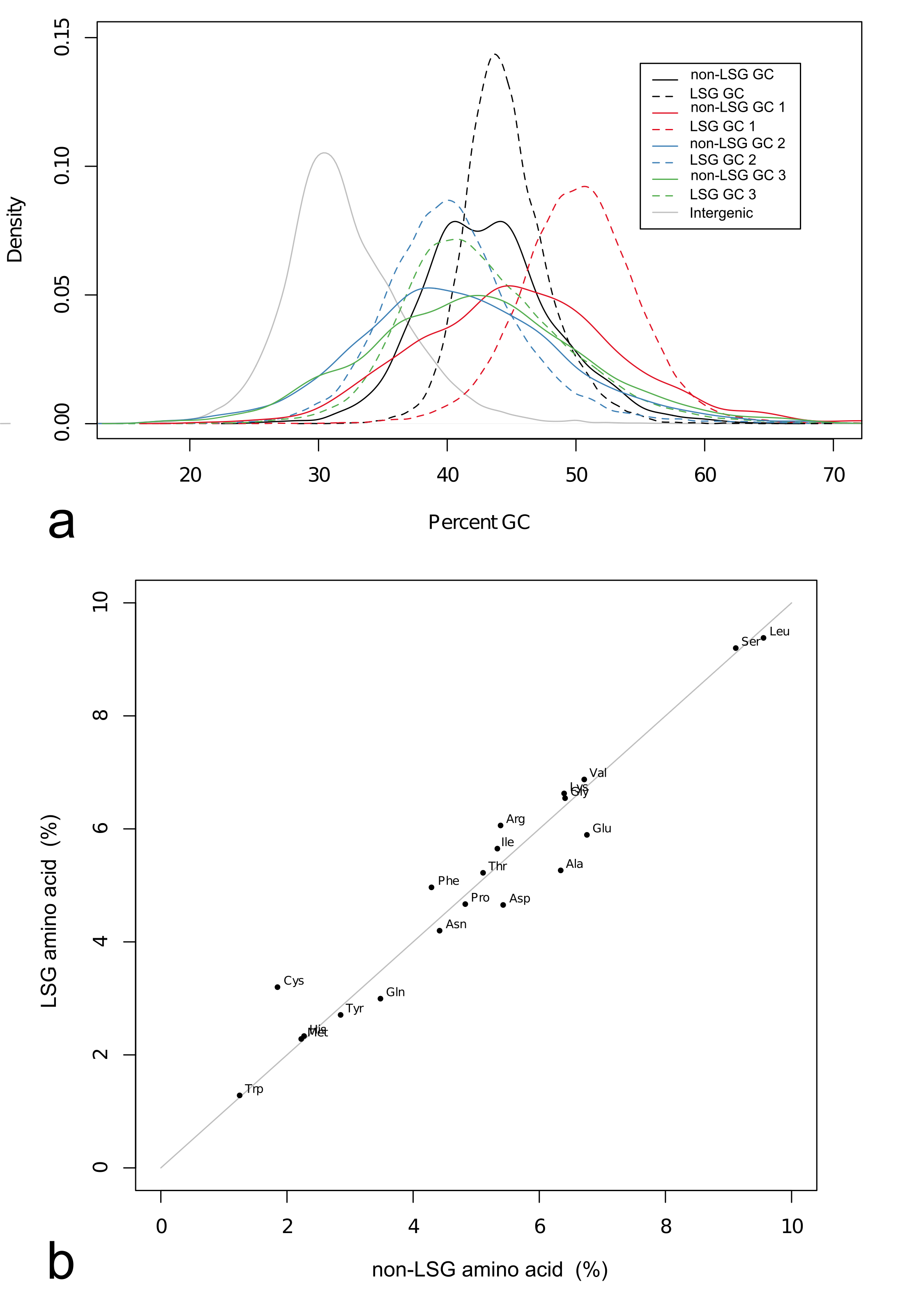
**

**Figure 1: GC and amino acid frequencies of LSGs and non-LSGs in *Arabidopsis thaliana*.**

**a) Percent GC content of LSGs and non-LSGs. Solid lines represent non-LSGs sequences. Dashed lines represent LSGs. Black lines represent overall GC, red lines represent GC1, blue lines represent GC2, green lines represent GC3 and the grey line represent intergenic GC content.**

**b) Amino acid percentage usage comparison between LSGs (y-axis) and non-LSGs (x-axis).**

## Motif enrichment in LS genes

To test whether any functionally conserved motifs, domains or gene classes were enriched in LSGs, protein "signatures" in all *Arabidopsis* protein coding genes were identified using InterProScan and associated databases [13]. Comparing the distribution of LSG and non-LSG protein signatures using hypergeometric tests identified enrichment for particular classes of protein signature, p-values were adjusted for multiple testing using FDR [14], **Table 2**. Five LSGs contain a β-tubulin auto-regulation binding site compared to 23 non-LSGs. Prokaryotic membrane lipoprotein lipid attachment site protein motifs are also enriched in LSGs (41 genes) but there are also a large number of non-LSGs containing lipoprotein attachment sites (167 genes) and conserved peptide upstream-ORF (CPuORF) genes previously identified [12, 15]

This study found that three domains of unknown function (DUF) are enriched in LSGs. These DUF LSG proteins are conserved within *Arabidopsis thaliana* but have no homologs in other species. DUF626 family has 14 non-LSG family members and eight LSG family members. DUF1163 family only has one non-LSG family member while all DUF1184 family members are LSGs.

Plant self-incompatibility response proteins are enriched in LSGs with 12 family members in comparison with six non-LSG family members. Both of the tapetum specific genes are LSGs. LSGs are also enriched for cysteine rich domains that contribute to the increased frequency cysteine amino acids found in LSGs. Similarly eukaryotic signal-peptides are enriched in LSGs (540 genes) whilst many non-LSG signal peptides are also reported (6127 genes). In addition to the InterPro results enrichment analysis was performed on the defensin-like protein family (DEFL) genes and (CPuORF) genes previously identified [12, 15]. Both DEFL and CPuORFs are enriched in LSGs compared to non-LSGs.

| **ID** | **Type** | **LSGs** | **Non-LSGs** | **P.value** | **Adj.P** | **Description** |
| --- | --- | --- | --- | --- | --- | --- |
| IPR013838 | Binding site | 5 | 23 | 0.03 | 0.07 | Beta tubulin, auto-regulation binding site |
| DEFL | NA | 115 | 37 | < 0.001 | NA | defensin-like DEFL family protein |
| CPuORF | NA | 30 | 34 | < 0.001 | NA | Conserved peptide upstream open reading frame |
| IPR006462 | Family | 8 | 14 | < 0.001 | < 0.001 | Protein of unknown function DUF626, Arabidopsis thaliana |
| IPR009544 | Family | 5 | 1 | < 0.001 | < 0.001 | Protein of unknown function DUF1163, Arabidopsis thaliana |
| IPR009568 | Family | 5 | 0.0 | < 0.001 | < 0.001 | Protein of unknown function DUF1184 |
| IPR009891 | Family | 2 | 0.0 | < 0.001 | < 0.001 | Tapetum specific TAP35TAP44 |
| IPR010682 | Family | 12 | 6 | < 0.001 | < 0.001 | Plant self-incompatibility response |
| PD032890 | PRODOM | 5 | 2 | < 0.001 | < 0.001 | Low molecular weight cysteine-rich |
| PD687080 | PRODOM | 3 | 1 | 0.001 | 0.003 | Low molecular weight precursor signal cysteine-rich |
| PD864278 | PRODOM | 5 | 1 | < 0.001 | < 0.001 | Low molecular weigh precursor signal cysteine-rich |
| PS51257 | PROFILE | 41 | 167 | < 0.001 | < 0.001 | Prokaryotic membrane lipoprotein lipid attachment site |
| SignalP-  HMM(euk) | SIGNALP | 540 | 6127 | < 0.001 | < 0.001 | Signal-peptide |
| No hits | No hits | 788 | 578 | < 0.001 | < 0.001 | No InterPro hits |

**Table 2: Protein signatures identified via InterProScan that are enriched for LSGs.**

## LSGs putatively involved in secretory pathways

Enrichment for secretory pathway motifs was also observed. Signal peptide motifs represent the largest group of LSGs that display enrichment of a functional motif. Previously work identifying unique genes in *Arabidopsis* found similar results; identifying that annotated unique genes with no rice homologs were enriched for peptide phytohormones when compared to unique conserved genes (conserved between *Arabidopsis* and rice) [16]. A number of those genes overlap or have divergent homologs with the LSG data set presented here indicating that these unique peptides not only lack a homolog in rice but also lack sequence similarity to any species outside of Brassicaceae, these include CLAVATA3 related peptides (two LSGs), POLARIS (one LSG), PROPEP peptides, RALF and RALF-like peptides (two LSGs) and hydroxyproline-rich glycoproteins (2 LSGs). Furthermore many cysteine rich anti-microbial like peptides have signal peptide motifs (in fact part of the requirement for the annotation of these genes was the presence of a signal peptide signature).

## LSGs are fast evolving

The ratio of the rate of non-synonymous substitutions (*d_N_*) to the rate of synonymous substitutions (*d_S_*) can be used as an indicator of selective pressure acting on a protein-coding gene. Higher rates of non-synonymous substitutions than synonymous substitutions (*d_N_*/*d_S_* > 1) provide evidence for adaptive evolution driven by Darwinian selection. An equal rate of non-synonymous substitutions and synonymous substitutions (*d_N_*/*d_S_* = 1) indicates neutral selection i.e. genetic drift. Lower rates of non-synonymous substitutions compared to synonymous substitutions (*d_N_*/*d_S_* < 1) provide evidence of purifying selection. Using a pair wise comparison of *Arabidopsis thaliana* vs. *Arabidopsis lyrata* orthologs a whole genome analysis of the *d_N_*/*d_S_* ratios were calculated using CodeML [17].

Using reciprocal BLASTP searches 21677 orthologous pairs were identified from the 27235 *Arabidopsis thaliana* gene models tested, including 511 LSGs. Those gene models not returning reciprocal BLAST results were not considered for *d_N_*/*d_S_* analysis. Those gene models tested were split into LSGs and non-LSGs and compared, **Figure 1**. The distribution of LSGs (dark gray) is shifted to the right of non-LSGs (light gray) indicating LSGs have a higher *d_N_*/*d_S_* than non-LSGs with medians of 0.5598 ± 0.2373 and 0.1772 ± 0.0968 respectively. Furthermore, a higher proportion of LSGs, 86 out of 511 (16.83%) have a *d_N_*/*d_S_* over one compared to 196 out of 19454 (1.01%) of non-LSGs. Note in both classes most genes have a *d_N_*/*d_S_* less than one possibly suggesting some level of purifying selection, however the *d_N_*/*d_S_* value, as calculated here, is an average across the whole ORF sequence. This average score is higher in LSGs than non-LSGs indicating weaker average purifying selection (across whole sequences) in LSGs than in non-LSGs even when that value is below one. The median *d_N_*/*d_S_* of stress responsive LSGs is 0.4870 ± 0.211.


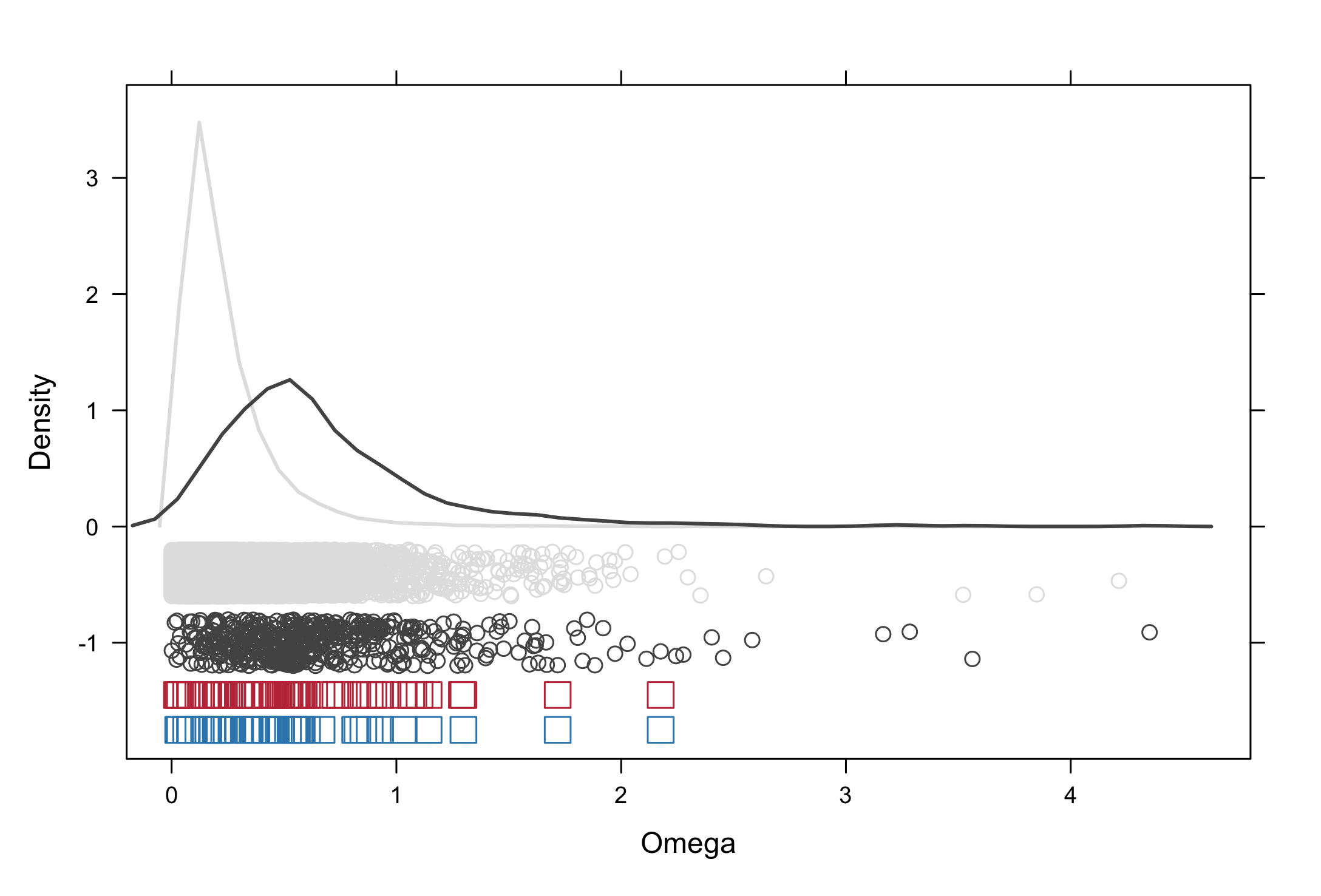


**Figure 2: *d_N_*/*d_S_* distributions of LSGs (dark gray) and non-LSGs (light gray). Stress responsive LSGs are highlighted in blue and red for up regulated and down regulated respectively.**

## Over 2% of LSGs display homology to intergenic or out-of-frame CDS in non-Brassicaceae species

Intergenic or out-of-frame CDS BLASTN hits of LSGs to non-Brassicaceae species can also identify the origins of LSGs. Two strategies were used to identify such significant hits in the fully sequenced genomes of rice, sorghum, grape, poplar and papaya. Firstly, syntelogs were identified; syntelogs represent a special case of gene homology where sets of genes are derived from the same ancestral genomic region. Syntelogs of LSGs to non-Brassicaceae CDS and genomic DNA were identified using SynMap (powered by DAGchainer [38]) part of the CoGe package [39]. This identified eleven putative LSG syntelogs, five with hits to non-Brassicaceae exons, five with hits to non-Brassicaceae non-coding genomic sequence and one with hits to non-coding genomic and exonic sequence in different species. There are no LSG syntelogs detected in rice or sorghum. LSGs are under represented for syntelogs in all species tested (hypergeometric test, p < 0.01). Secondly, non-syntelog reciprocal BLAST hits were also identified in both CDS and genomic sequence. Thirty LSGs have non-syntelog reciprocal hits to non-Brassicaceae species, ten exonic and 19 non-coding genomic and one with hits to non-coding genomic and exonic sequence in different species. The evolutionary origins of 2.29% of LSGs can be identified by this approach in the species tested.

# Supplementary Materials and Methods

## InterProScan and enrichment of signatures in LSGs

All representative gene models (peptide sequences) were analyzed using a stand-alone version of InterProScan using the v19 of the InterPro library. Results were parsed and stored in a postgresql table. An additional category of no hits was added for all gene models without any hits. The number of signature hits for LSGs and non-LSGs was calculated for each signature with a hit within an LSG. Enrichment analysis for all signatures with hits in LSGs was performed the confidence interval was set at an adjusted p-value < 0.05 using FDR [14].

## *d_N_*/*d_S_* analysis

**Data retrieval:** *Arabidopsis lyrata* peptide sequences v1 were download from the Joint Genome Initiative [18].

Formatdb was used to produce an *Arabidopsis lyrata* peptides database. Reciprocal BLASTP searches were performed between *Arabidopsis thaliana* vs. *Arabidopsis lyrata* (i.e. *Arabidopsis thaliana* peptides vs. *Arabidopsis lyrata* peptide database and *Arabidopsis lyrata* peptides vs. *Arabidopsis thaliana* peptide database). The reciprocal top hit sequences were then aligned at the peptide level using MUSCLE [19]. Using the peptide alignment as a template the reciprocal top hit CDS sequences were then aligned using the tranalign program from the EMBOSS package. Pair wise *d_N_*/*d_S_* analysis was then performed on the CDS alignments using both the CodeML program (using model 0 and runmode -2) and yn00 both from the PAML package [17]. The results were split into two groups LSGs and non-LSGs. Summary statistics and Wilcoxon rank sum test were calculated in R.

## Identifying syntelogs

Syntelogs were identified using SynMap, part of the CoGe comparative genomics platform [39]. Pair wise syntelog maps were produced aligning *Arabidopsis thaliana* CDS to papaya CDS, poplar CDS, grape CDS, rice CDS and sorghum CDS. In addition, syntelog maps were produced using *Arabidopsis thaliana* CDS sequence aligned to papaya, poplar, grape, and rice and sorghum genomic sequences. In each case the default (relatively relaxed) settings were used (i.e. average distance between pairs = 10, maximum distance between pairs = 20, minimum aligned pairs = 5). Syntelogs between *Arabidopsis thaliana* and *Arabidopsis lyrata* (CDS and intergenic) were identified using a more stringent setting (i.e. average distance between pairs = 2, maximum distance between pairs = 5, minimum aligned pairs = 3).

LSGs were parsed out from the SynMap results and were identified as LSG syntelogs. No alignment percentage coverage minimum was used for the syntelog results.

# References
