# Supplementary material for: Evolutionary origins of Brassicaceae specific genes in Arabidopsis thaliana
Source: BMC Evol Biol. 2011 Feb 18;11:47. doi: 10.1186/1471-2148-11-47 (PMC3049755; doi:10.1186/1471-2148-11-47)
Supplement: Additional file 6 — Distributions of E-values for LSG non-LSG paralog BLASTP and BLASTN hits. [file 1471-2148-11-47-S6.PDF]

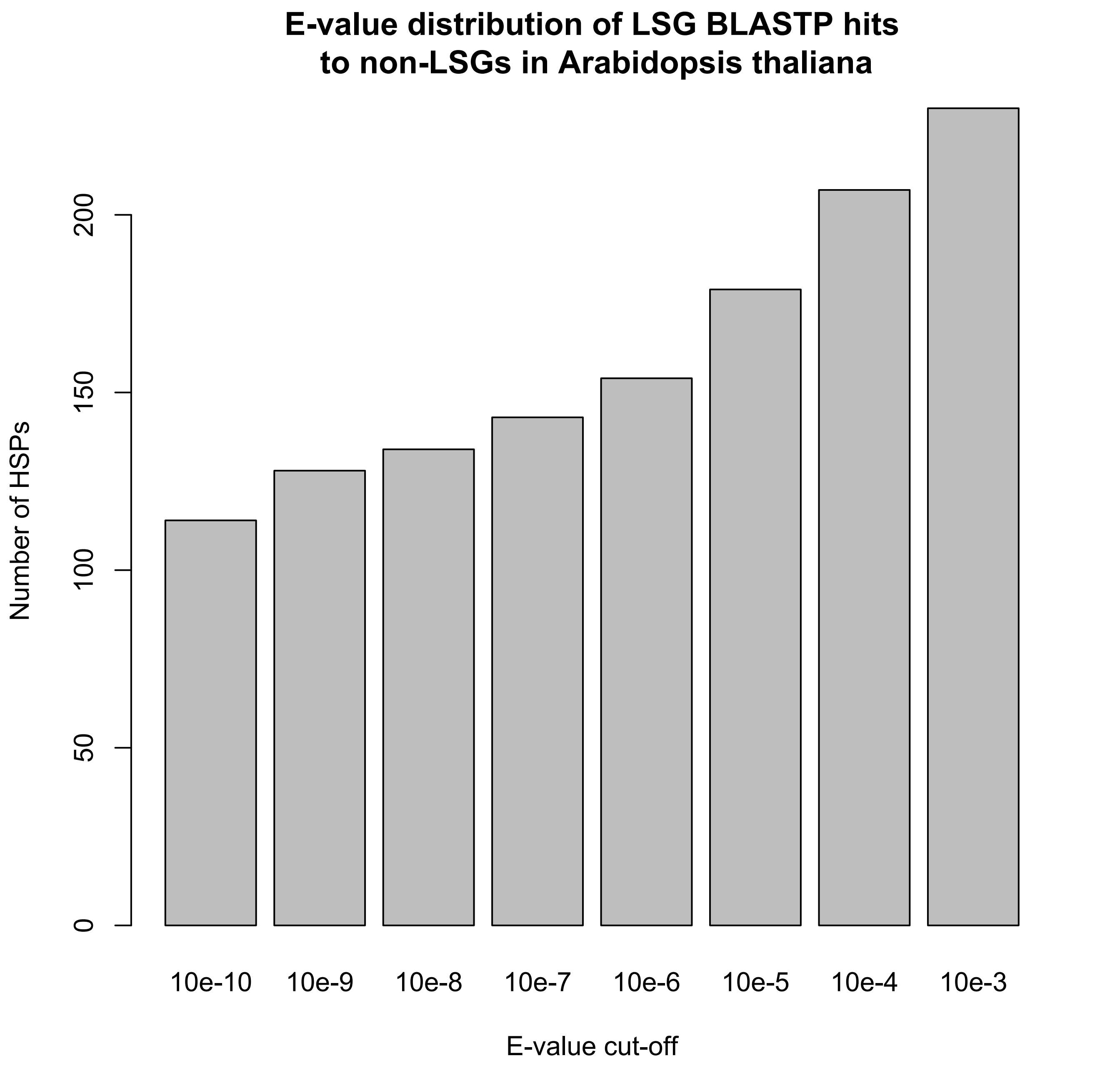

E-value distribution of LSG BLASTP hits
to non-LSGs in Arabidopsis thaliana
Number of HSPs
0
50
100
150
200
10e-10
10e-9
10e-8
10e-7
10e-6
10e-5
10e-4
10e-3
E-value cut-off

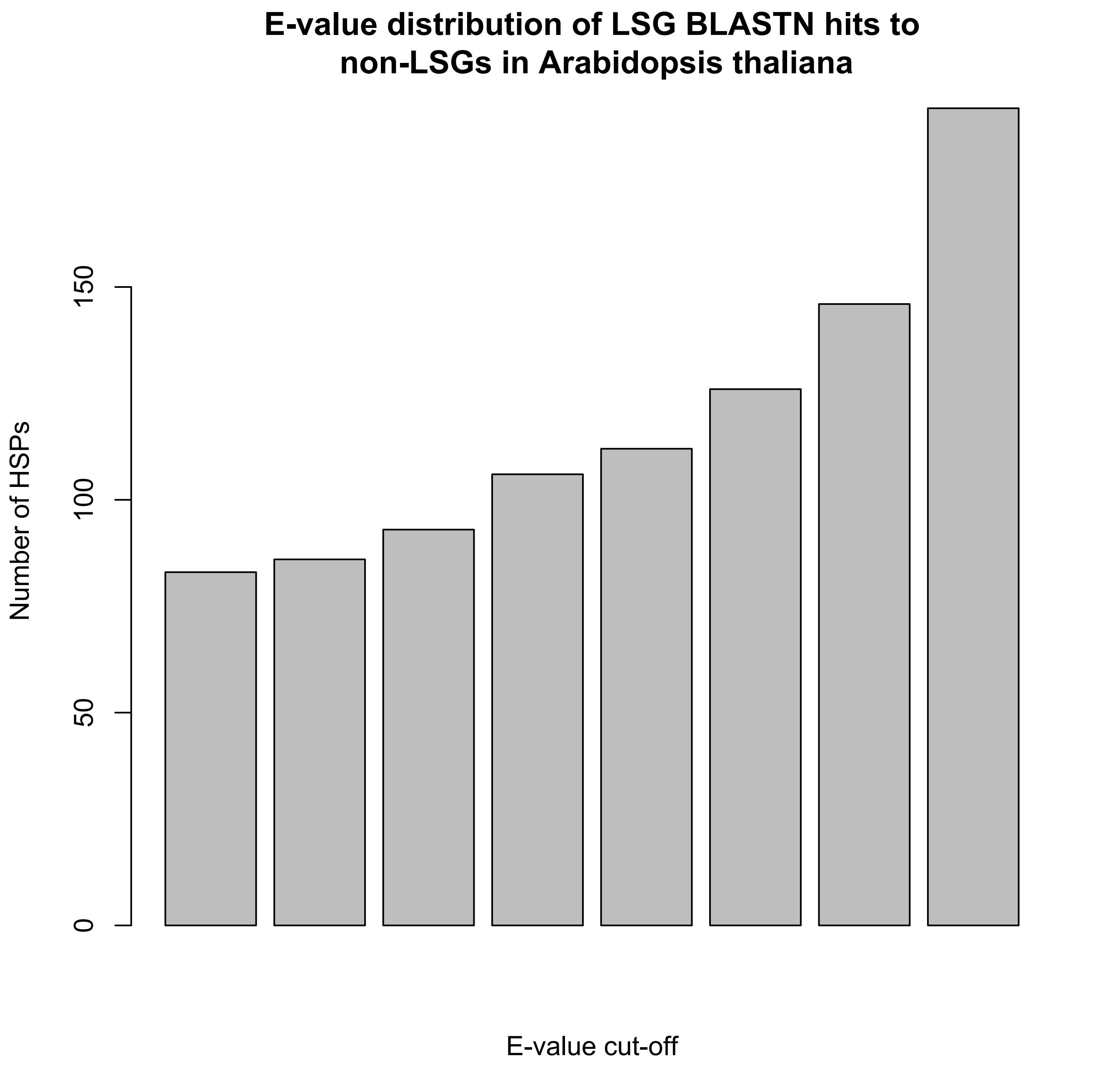
E-value distribution of LSG BLASTN hits to
non-LSGs in Arabidopsis thaliana
Number of HSPs
0
50
100
150
E-value cut-off
