# Supplementary material for: Evolutionary origins of Brassicaceae specific genes in Arabidopsis thaliana
Source: BMC Evol Biol. 2011 Feb 18;11:47. doi: 10.1186/1471-2148-11-47 (PMC3049755; doi:10.1186/1471-2148-11-47)
Supplement: Additional file 10 — Alignments and gene models of sequenced LSGs in various accessions and sister species. For the multiple sequence alignments: black = identical residues, blue = similar residues, red = other residues (i.e. non-matching). In addition, for the gene model alignments "!" Indicates indel and the number of nucleotides are displayed below. For ambiguous nucleotides; m = A or C, y = C or T and w = A or T and "x" = undetermined peptide. [file 1471-2148-11-47-S10.PDF]

AT4G02465.1

```
Col-0          MAIKWKRKAIRVVASSSGSVWIGRYLIDDDDVDEQSQEFQIQLNYQDMAT       50
Got-7          MAIKWKRKAIRVVASSSGSVWIGRYLIDDDDVDEQSQEFQIQLNYQDMAT       50

Col-0          EEEDKLLDALLSKDGASIAKRLSNCCFAKFGGVGIFMAEQVSSSKLFWRR       100
Got-7          EEEDKLLDALLSKDGASIAKRLSNCCFAKFGGVGIFMAEQVSSSKLFWRR       100

Col-0          DMQWRIGRLMRLPLTSLDFIRRRMLYPSFGTKHFLPLCNGK*     142
Got-7          DMQWRIGRLMRLPLTSLDFIRRRMLYPSFGTKHFLPLCNGK      141
```

Arabidopsis lyrata petraea plech 14-5 gene model produced using genewise

```
Col-0            26 LIDDDDVDEQSQEFQIQLNYQ                     DMAT-
                    LIDDDDVDE+SQEFQ QL+ Q                     DM +
                    LIDDDDVDEKSQEFQTQLDNQ                     DMTSC
plech 14-5      298 taggggggatcgtcaccgacGTCAGAT  Intron 1   CAGgaaat
                    ttaaaataaacaatacataaa<0-----[361  :  427]-0>atcgg
                    gttcttttgatagtgcattca                     tgcct


Col-0            51 EEEDKLLDALLSKDGASIAKRLSNCCFAKFGGVGIFMAEQVSSSK
                    EEEDK+LDAL SKDGASIAKRLS+          I+   Q    K
                    EEEDKILDALFSKDGASIAKRLSH---------AIYNFYQ!MKIK
plech 14-5      443 ggggaacggtttagggtagacctc         gatattc5aaaa
                    aaaaattacttcaagcctcagtca         ctaataa tata
                    gaacgatttgtcgtataatgtctt         tatccta ggag


Col-0            96                       LFWRRDMQWRIGRLMRLPLTSLDFIR
                                          LFWRRDMQWRIGRLMR  L S +FIR
                                          LFWRRDMQWRIGRLMRSLLISXEFIR
plech 14-5      553 GTGATGC  Intron 2   AAGtttaagactcagctactccattgtaa
                    <0-----[553  : 1535]-0>ttgggataggtggttgctttcgattg
                                          acgaatgagtcgggggagcttaacag


Col-0           122 RRMLYPSFGTKHFLPLCNGK
                    RR L PSFGTKHFLPLC GK
                    RRKLCPSFGTKHFLPLCYGK
plech 14-5     1614 acattcttgaacttctttga
                    ggatgcctgcaattctgaga
                    aagacgatccaccgtgcttg
```

Arabidopsis lyrata lyrata (JGI draft) gene model produced using genewise

```
Col-0                26 LIDDDDVDEQSQEFQIQLNYQ                     DMAT-
                        LIDDDDVDE+SQEFQ QL+ Q                     DM +
                        LIDDDDVDEKSQEFQTQLDNQ                     DMTSC
A.lyrata lyrata     298 taggggggatcgtcaccgacGTCAGAT  Intron 1   CAGgaaat
                        ttaaaataaacaatacataaa<0-----[361  :  427]-0>atcgg
                        gttcttttgatagtgcattca                     tgcct


Col-0                51 EEEDKLLDALLSKDGASIAKRLSNCCFAKFGGVGIFMAEQVSSSK
                        EEEDK+LDAL SKDGASIAKRLS+          I+   Q    K
                        EEEDKILDALFSKDGASIAKRLSH---------AIYNFYQ!MKIK
A.lyrata lyrata     443 ggggaacggtttagggtagacctc         gatattc5aaaa
                        aaaaattacttcaagcctcagtca         ctaataa tata
                        gaacgatttgtcgtataatgtctt         tatccta ggag


Col-0                96                       LFWRRDMQWRIGRLMRLPLTSLDFIR
                                              LFWRRDMQWRIGRLMR  L S +FIR
                                              LFWRRDMQWRIGRLMRSLLISXEFIR
A.lyrata lyrata     553 GTGATGC  Intron 2   AAGtttaagactcagctactccattgtaa
                        <0-----[553  : 1535]-0>ttgggataggtggttgctttcgattg
                                              acgaatgagtcgggggagcttaacag


Col-0               122 RRMLYPSFGTKHFLPLCNGK
                        RR L PSFGTKHFLPLC GK
                        RRKLCPSFGTKHFLPLCYGK
A.lyrata lyrata    1614 acattcttgaacttctttga
                        ggatgcctgcaattctgaga
                        aagacgatccaccgtgcttg
```

AT1G61165.1

```
Col-0            MLMLGDDIASFIAYPTPAPGDSNSRTSSPNSIQEC*    36
CS22491          MLMLGDDIASFIAYPTPAPGDSNSRTSSPNSIQEC*    36
Ag-0             MLMLGDDIASFIAYPTPAPGDSNSRTSSPNSIQEC*    36
Kas-1            MLILGDDIASFIAYPTPAPGDSNSRTSSPNSIQEY*    36
CVI-0            VLMLGDDIASFIAYPTPAPEDSSSRTSSPNSIQEC*    36
Bay-0            VLMPGDDIASFIAYPTPAPQDSSSRTSSPNSIQEC*    36
A.lyrata_lyrata  VLMLGEDIPTFIAYPASAPCVSNNRNSSPNSILECE    36
```

AT3G30160.1

```
Col-0            MIICIYLPLHTASSHVFFITITSLSHISIPAIIIIVVTPELTSRCGRKCS    50
CVI-0            MIICIYLPLHTASSHVFFITITSLSHISIPAIIIIVVTPELTSRCGRKCS    50
Kas-1            MIICIYLPLHTASSHVFFITITSLSHISIPAIIIIVVTPELTSRCGRKCS    50
CS22491          MIICIYLPLHTASSHVFFITITSLSHISIPAIIIIVVTPELTSRCGRKCS    50
TS-1             MIICIYLPLHTASSHAFFITITSLSHISIPAIIIIVVTPELTSRCGRKCS    50
OY-0             MIICIYLPLHTASSHVFFITITSLSHISVPAIIIVVVTPELTSRCGRKCS    50
NOK-3            MIICIYLPLHTASSHAFFITITSLSHISVPAIIIVVVTPELTSRCGRKCS    50
Bay-0            MIICIYLPLHTASSHAFFITITSLSHISVPAIIIVVVTPELTSRCGRKCS    50

Col-0            TWLVSSPFIAGLL*    64
CVI-0            TWLVSSPFIAGLL*    64
Kas-1            TWLVSSPFIAGLL*    64
CS22491          TWLVSSPFIAGLL*    64
TS-1             TWLVSSPFIAGLL*    64
OY-0             TWLVSSPFIAGLL*    64
NOK-3            TWLVSSPFIAGLL*    64
Bay-0            TWLVSSPFIAGLL*    64
```

AT4G31960.1

```
Ag-0             MLTGLVVDISMTSTAPTSPSRSESRQLQDPFGEVETKTQNDSVVSNKDKG    50
OY-0             MLTGLVVDISMTSTAPTSPSRSESRQLQDPFGEVETKTQNDSVVSNKDKG    50
NOK-3            MLTGLVVDISMTSTAPTSPSRSESRQLQDPFGEVETKTQNDSVVSNKDKG    50
TS-1             MLTGLVVDISMTSTAPTSPSRSESRQLQDPFGEVETKTQNDSVVSNKDKG    50
Col-0            MLTGLVVDISMTSTAPTSPSRSESRQLQDPFGEVETKTQNDSVVSNKDKG    50
CVI-0            MLTGLVVDISMTSTAPTSPSRSESRQLQDPFGEVETKTQNDSVVSNKDKG    50
Bay-0            MLAGLVVDISMTSTAPTSPSRSESRQLQDPFGEVETKTQNDSVVSNKDKG    50

Ag-0             LLGQKPNDNKTIKELKPKSAVSSYISQRSIPPVQANKTRVDTRKRSVMKG    100
OY-0             LLGQKPNDNKTIKELKPKSAVSSYISQRSIPPVQANKTRVDTRKRSVMKG    100
NOK-3            LLGQKPNDNKTIKELKPKSAVSSYISQRSIPPVQANKTRVDTRKRSVMKG    100
TS-1             LLGQKPNDNKTIKELKPKSAVSSYISQRSIPPVQANKTRVDTRKRSVMKG    100
Col-0            LLGQKPNDNKTIKELKPKSAVSSYISQRSIPPVQANKTRVDTRKRSVMKG    100
CVI-0            LLGQKPNDNKTIKELKPKSAVSSYISQRSIPPVQANKTRVDTRKRSVMKG    100
Bay-0            LLGQKPNDNKTIKELKPKSAVSSYISQRSIPPVQANKTRVDTRKRSVMKG    100

Ag-0             CTDPIITTNRFDCLATCET*    120
OY-0             CTDPIITTNRFDCLATCET*    120
NOK-3            CTDPIITTNRFDCLATCET*    120
TS-1             CTDPIITTNRFDCLATCET*    120
Col-0            CTDPIITTNRFDCLATCET*    120
CVI-0            CTDPIITTNRFDCLATCET*    120
Bay-0            CTDPIITTNRFDCLATCET*    120
```

AT5G53144.1

Col-0	MGLYHTFGVSVMAKFGFDFRVVEFVPFVCRSV*	33
CVI-0	MGLYHTFGVSVMAKFGFDFRVVEFVPFVCRSV*	33
Ag-0	MGLYHTFGVSVMAKFGFDFRVVEFVPFVCRSV*	33
Bay-0	MGLYHTFGVSVMAKFGFDFRVVEFVPFVCRSV*	33
Kas-1	MGLYHTFGVSVMAKFGFDFRVVEFVPFVCRSV*	33
CS22491	MGLYHTFGVSVMAKFGFDFRVVEFVPFVCRSV*	33
A.lyrata_lyrata	LGLYHTFGVSVMAKFGFDFRVVELVQFVCRSV*	33

AT5G57567.1

Ag-0	MLMELQAEKEAILAVEAAAIAKALSKLPAEEEAELGKAESKE*	42
CVI-0	MLMELQAEKEAILAVEAAAIAKALSKLPTEEEAELGKAESKE*	42
OY-0	MLMELQAEKEAILAVEAAAIAKALSKLPTEEEAELGKAESKE*	42
CS22491	MLMELQAEKEAILAVEAAAIAKALSKLPTEEEAELGKAESKE*	42
Bay-0	MLMELQAEKEAILAVEAAAIAKALSKLPTEEEAELGKAESKE*	42
Col-0	MLMELQAEKEAILAVEAAAIAKALSKLPTEEEAELGKAESKE*	42

AT4G38781.1

```
CVI-0_1                              MPSYIYVRSVNRL-SYQPHPTTVILGIFVFVKARTEVLGFCSSAILAGPLDLKS  53
Ag-0_1                               MPSYIYLRSVNRL-SYQPHPTTVILGIFVFVKARTEVLGFCSSAILLG-LWI-*  51
Bay-0_1                              MPSYIYLRSVNRL-SYQPHPTTVILGIFVFVKARTEVLGFCSSAILLG-LWI-*  51
CS22491_1                            MPSYIYLRSVNRL-SYQPHPTTVILGIFVFVKARTEVLGFCSSAILLG-LWI-*  51
Col-0_1                              MPSYIYVRSVNRL-SYQPHPTTVILGIFVFVKARTEVLGFCSSAILLG-LWI-*  51
Kas-1_1                              MPSYIYVRSVNRL-SYQPHPTTVILGIFVFVKARTEVLGFCSSAILLG-LWI-*  51
A.cebennensis                        LSIYMFVRLIVFL-TYRPHPTTVILGFFVFIKARTEVLGLFFSAI-----WIC*  48
A.lyrata_petraea_Tannenberg_T1a      IYIYMFVRLIVFLNSNRPHPTTVILGFFVFIKARTEVLGLFFSGIWLV-LWIC*  53
A.lyrata_lyrata                      IYIYMFVRLIVFLNSNRPHPTTVILGFFVFIKARTEVLGLFFSAIWLV-LWIC*  53
```

Arabidopsis_lyrata_petraea_NT_12b
gene model produced using genewise

```
Arabidopsis_thaliana_Col-0          MPSYIYVRSVNRLSY--QPHPTTVILGIFVFVKARTEVLGFCSSAILLGLWI-*
                                       YIYVRSVNRLS    PHPTTVILG+FVF+KARTEVLG+  SAI L LWI *
Arabidopsis_lyrata_petraea_NT12b    LYxYIYVRSVNRLS!SNRPHPTTVILGFFVFIKARTEVLGLFFSAIWLVLWIC*
                                    ctmtatgctgacct4taccccaagatgttgtaagcaggtgctttgatcgctatt
                                    tatatatgctagtc cagcaccctttgttttacgcattgtttcctgtttgtgg
                                    ctatattcgttttt tcatcggggtagtcgccgtcggtagtccagtggctgtta
```

AT5G50361.1

```
CVI-0      MCLPLIAPHLIATPRSPIGHHFPRTSSLNVGFKQNATSLTFVQSRRCVVHRLVSHVARFSPNVST*  66
Ag-0       MCLPLIAPHLIATPRSPIGHHFPRTSSLNVGFKQNATSLTFVQSRRCAVPRLVSHVARFSPNVST*  66
Kas-1      MCLPLIAPHLIATPRSPIGHHFPRTSSLNVGFKQNATSLTFVQSRRCAVHRLVSHVARFSPNVST*  66
Bay-0      MCLPLIAPHLIATPRSPIGHHFPRTSSLNVGFKQNATSLTFVQSRRCAVHRLVSHVARFSPNVST*  66
CS22491    MCLPLIAPHLIATPRSPIGHHFPRTSSLNVGFKQNATSLTFVQSRRCAVHRLVSHVARFSPNVST*  66
Col-0      MCLPLIAPHLIATPRSPIGHHFPRTSSLNVGFKQNATSLTFVQSRRCAVHRLVSHVARFSPNVST*  66
```

Arabidopsis_lyrata_petraea_Tannenberg_T1a
gene model produced using genewise

```
Arabidopsis_thaliana_Col-0                   MCLPLIAPHLIATPRSPIGHHFPRTSSLNVGFKQNATSLTFVQSRRCAVHRLVSHVARFSPNVST*
                                             M +PLIAPH IATPRS IGHHFPRTSSL VGFKQNATSL FVQSRRCAVHRLVSHVA+FSPNVST*
Arabidopsis_lyrata_petraea__Tannenberg_T1a   MWIPLIAPHxIATPRSxIGHHFPRTSSL!VGFKQNATSL!FVQSRRCAVHRLVSHVAKFSPNVST*
                                             atactagcccagacctcagcctccatac2ggtacagatc4tgctcctggcctgtcggattcagtat
                                             tgtcttccawtcccgcytgaatcgccgt tgtaaaccct ttacgggctagttcatcatccatcca
                                             ggwtacagcaacttctyttccctttgtc cacaaccgta ccaattcttttgcgcgagttacggga
```

Arabidopsis_lyrata_petraea_NT_12b
gene model produced using genewise

```
Arabidopsis_thaliana_Col-0          MCLPLIAPHLIATPRSPIGHHFPRTSSLNVGFKQNATSLTFVQSRRCAVHRLVSHVARFSPNVST*
                                    M +PLIAPHLIATPRSPIGHHFPRTSSL VGFKQNATSL FVQSRRCAVHRLVSHVA+FSPNVST*
Arabidopsis_lyrata_petraea_NT12b    MWIPLIAPHLIATPRSPIGHHFPRTSSL!VGFKQNATSL!FVQSRRCAVHRLVSHVAKFSPNVST*
                                    atactagcccagacctcagcctccatac2ggtacagatc4tgctcctggcctgtcggattcagtat
                                    tgtcttccattcccgcctgaatcgccgt tgtaaaccct ttacgggctagttcatcatccatcca
                                    ggatacagcaacttcttttccctttgtc cacaaccgta ccaattcttttgcgcgagttacggga
```

Arabidopsis_cebennensis
gene model produced using genewise

```
Arabidopsis_thaliana_Col-0          MCLPLIAPHLIATPRSPIGHHFPRTSSLNVGFKQNATSLTFVQSRRCAVHRLVSHVARFSPNVST*
                                    M LPLIAPHLIATPRSPIGHH PRTSSL VG KQNATSL FVQSRRCAVHRLVSHVA+FSPNVST*
Arabidopsis_cebennensis             MWLPLIAPHLIATPRSPIGHHSPRTSSL!VGPKQNATSL!FVQSRRCAVHRLVSHVAKFSPNVST*
                                    atcctagcccagacctcagcctccatac2ggcacagatc4tgctcctggcctgtcggattcagtat
                                    tgtcttccattcccgcctgaaccgccgt tgcaaaccct ttacgggctagttcatcatccatcca
                                    ggatacagcaacttcttttccctttgtc cacaaccgta ccaattcttttgcgcgagttacggga
```

Arabidopsis_lyrata_lyrata
gene model produced using genewise

```
Arabidopsis_thaliana_Col-0          MCLPLIAPHLIATPRSPIGHHFPRTSSLNVGFKQNATSLTFVQSRRCAVHRLVSHVARFSPNVST*
                                    M LPLIAPHLIATPRS IGHHFPRTSSL VGFKQNATSL FVQSRRCAVHRLVSHVA+FSPNVST*
Arabidopsis_lyrata_lyrata           MWLPLIAPHLIATPRSLIGHHFPRTSSL!VGFKQNATSL!FVQSRRCAVHRLVSHVAKFSPNVST*
                                    atcctagcccagacctcagcctccatac2ggtacagatc4tgctcctggcctgtcggattcagtat
                                    tgtcttccattcccgcttgaatcgccgt tgtaaaccct ttacgggctagttcatcatccatcca
                                    ggatacagcaacttctcttccctttgtc cacaaccgta ccaattcttttgcgcgagttacggga
```

AT2G36854.1

Arabidopsis lyrata lyrata (JGI draft) gene model produced using genewise

```
Col-0              2 SLKRVKHHGTKREDEE-----TRIARAGGFEPGCDGGVSDGIYGGFKKT
                     +L RVKHHGTKREDEE     TR ARAG FEPGCDGGVSDG YGGFKKT
                     ALNRVKHHGTKREDEEEEAV!TRKARAGRFEPGCDGGVSDG!YGGFKKT
A.lyrata lyrata   10 gcaagaccgaaagggggggg2acagcggctgcgtggggtgg2tggtaaa
                     ctagtaaagcagaaaaaact cgacgcggtacggaggtcag aggtaac
                     cacggactcagaatagaatg gaatataacacatcatggca tcatgag


Col-0             46 SRFDSFVKWVFSKK
                     SRFDSFVKWVFS++
                     SRFDSFVKWVFSEE
A.lyrata lyrata  155 actgttgatgttgg
                     ggtacttagttcaa
                     tattccaggtcaga



Col-0              2 SLKRVKHHGTKREDEE-----TRIARAGGFEPGCDGGVSDGIYGGFKKT
                     +L RVKHHGTKREDEE     TR ARAG FEPGCDGGVSDG YGGFKKT
                     ALNRVKHHGTKREDEEEEAV!TRKARAGRFEPGCDGGVSDG!YGGFKKT
plech 8-3         63 gcaagaccgaaagggggggg2acagcggctgcgtggggtgg2tggtaaa
                     ctagtaaagcagaaaaaact cgacgcggtacggaggtcag aggtaac
                     cacggactcagaatagaatg gaatataacacatcatggca tcatgag


Col-0             46 SRFDSFVKWVFSKKIK
                     SRFDSFVKWVFS+  K
                     SRFDSFVKWVFSExKK
plech 8-3        208 actgttgatgttgNaa
                     ggtacttagttcaaaa
                     tattccaggtcagaga
```

Arabidopsis lyrata petraea plech 9-7 gene model produced using genewise

```
Col-0              2 SLKRVKHHGTKREDEE-----TRIARAGGFEPGCDGGVSDGIYGGFKKT
                     +L RVKHHGTKREDEE     TR ARAG FEPGCDGGVSDG YGGFKKT
                     ALNRVKHHGTKREDEEEEAV!TRKARAGRFEPGCDGGVSDG!YGGFKKT
plech 9-7         63 gcaagaccgaaagggggggg2acagcggctgcgtggggtgg2tggtaaa
                     ctagtaaagcagaaaaaact cgacgcggtacggaggtcag aggtaac
                     cacggactcagaatagaatg gaatataacacatcatggca tcatgag


Col-0             46 SRFDSFVKWVFSKKIK
                     SRFDSFVKWVFS++ K
                     SRFDSFVKWVFSEEKK
plech 9-7        208 actgttgatgttggaa
                     ggtacttagttcaaaa
                     tattccaggtcagaga
```

Arabidopsis lyrata petraea plech 11-2 gene model produced using genewise

```
Col-0              2 SLKRVKHHGTKREDEE-----TRIARAGGFEPGCDGGVSDGIYGGFKKT
                     +L RVKHH TKREDEE     TR ARAG FEPGCDGGVSDG YGGFKKT
                     ALNRVKHHDTKREDEEEEAV!TRKARAGRFEPGCDGGVSDG!YGGFKKT
plech 11-2        48 gcaagaccgaaagggggggg2acagcggctgcgtggggtgg2tggtaaa
                     ctagtaaaacagaaaaaact cgacgcggtacggaggtcag aggtaac
                     cacggactcagaatagaatg gaatataacacatcatggca tcatgag


Col-0             46 SRFDSFVKWVFSKKIK
                     SRFDSFVKWVFS+K K
                     SRFDSFVKWVFSEKKK
plech 11-2       193 actgttgatgttgaaa
                     ggtacttagttcaaaa
                     tattccaggtcagaga
```

Arabidopsis lyrata petraea plech 13-4 gene model produced using genewise

```
Col-0              2 SLKRVKHHGTKREDEE-----TRIARAGGFEPGCDGGVSDGIYGGFKKT
                     +L RVKHHGTKREDEE     TR ARAG FEPGCDGGVSDG YGGFKKT
                     ALNRVKHHGTKREDEEEEAV!TRKARAGRFEPGCDGGVSDG!YGGFKKT
plech 13-4        97 gcaagaccgaaagggggggg2acagcggctgcgtggggtgg2tggtaaa
                     ctagtaaagcagaaaaaact cgacgcggtacggaggtcag aggtaac
                     cacggactcagaatagaatg gaatataacacatcatggca tcatgag


Col-0             46 SRFDSFVKWVFSKKIK
                     SRFDSFVKWVFS+  K
                     SRFDSFVKWVFSExKK
plech 13-4       242 actgttgatgttgNaa
                     ggtacttagttcaaaa
                     tattccaggtcagaga
```

AT5G08220.1

Arabidopsis lyrata lyrata (JGI draft) gene model produced using genewise

```
Col-0              1 MTDAFLSNCHSIEPVLQEYPYRYFAHCLSR
                     MT AFL+NC S+ PVLQEY YRY+AHCLS
                     MTHAFLNNCDSMVPVLQEYSYRYLAHCLSE
A.lyrata lyrata    4 aacgtcaatgtagcgtcgtttctcgctttg
                     tcacttaagacttcttaaacagatcagtca
                     gatatactcccgggtaaatgtattgccaaa


Col-0             31                        VYTFGLGLVEMSVLVGQSFGVIS
                                            VYTF  G+VEM+VLVG S+G IS
                                            VYTFRPGVVEMNVLVGPSLG!IS
A.lyrata lyrata   94 GTTTGTT  Intron 1   TAGgtatacggggaagcggcatg4at
                     <0-----[94   :   433]-0>tactgcgttatatttgcgtg tc
                                            tcgtacataggccgtaatac cg


Col-0             54                           VLGNFVEPEESSKVIGN
                                               VLGNFVEPE SSKVIGN
                                R:R[agg]       VLGNFVEPE-SSKVIGN
A.lyrata lyrata  504 AGGTAAAGA  Intron 2   CAGGgtgatggcg atagaga
                       <2-----[506   :   753]-2> ttgattaca gcattga
                                               agattgagg tcagtgc
```

Arabidopsis lyrata petraea plech 11-2 gene model produced using genewise

```
Col-0              6 LSNCHSIEPVLQEYPYRYFAHCLSR                   V
                     L+ C S+EPVLQEY YRY+AHCLS                    V
                     LNICDSMEPVLQEYSYRYLAHCLSE                   V
plech 11-2         3 taatgtagcgtcgtttctcgctttgGTTTGTT  Intron 1   TAGg
                     tatgactacttaaacagatcagtca<0-----[78   :   413]-0>t
                     actcccgggtaaatgtattgccaaa                   t


Col-0             32 YTFGLGLVEMSVLVGQSFGVIS                      V
                     YTF  G+VEM+VLVG S+G IS                      V
                     YTFRPGVVEMNVLVGPSLG!IS          R:R[agg]    V
plech 11-2       417 tatacggggaagcggcatg4atAGGTAAAGA  Intron 2   CAGGg
                     actgcgttatatttgcgtg tc  <2-----[486   :   733]-2> t
                     cgtacataggccgtaataa cg                      a


Col-0             56 LGNFVEPEESSKVIGN
                     LGNFVEPE SSKVIGN
                     LGNFVEPE-SSKVIGN
plech 11-2       738 tgatggcg atagaga
                     tgattaca gcattga
                     gattgagg tcagtgc
```

AT2G29654.1

```
Bur-0    ATGATTGAAATGGTGAATGATAATTGAAGAAGTGGGCCACGTGAGAGAAGCATGTACTTT   60
Col-0    ATGATTGAAATGGTGAATGATAATTGGAGAAGTGGGCCACGTGAGAGAAGCATGTACTTT   60
Got-7    ATGATTGAAATGGTGAATGATAATTGGAGAAGTGGGCCACGTGAGAGAAGCATGTACTTT   60
Cvi-0    ATGATTGAAATGGTGAATGATAATTGAAGAAGTGGGCCACGTGAGAGAAGCATGTACTTT   60
Ler-1    ATGATTGAAATGGTGAATGATAATTGAAGAAGTGGGCCACGTGAGAGAAGCATGTACTTT   60
Bay-0    ATGATTGAAATGGTGAATGATAATTGAAGAAGTGGGCCACGTGAGAGAAGCATGTACTTT   60

Bur-0    TGGAAAGTGGGTCATTTCGAAGCTCTTCCTGTCTCCATCCACATGAAGTTGGCATT----   116
Col-0    TGGAAAGTGGGTCATTTCGAAGCTCTTCCTGTCTCCATCCACATGAAGTTGGCATTCTTG   120
Got-7    TGGAAAGTGGGTCATTTCGAAGCTCTTCCTGTCTCCATCCACATGAAGTTGGCATTCTTG   120
Cvi-0    TGGAAAGTGGGTCATTTCGAAGCTCTTCCTGTCTCCATCCACATGAAGTTGGCATTCTTG   120
Ler-1    TGGAAAGTGGGTCATTTCGAAGCTCTTCCTGTCTCCATCCACATGAAGTTGGCATTCTTG   120
Bay-0    TGGAAAGTGGGTCATTTCGAAGCTCTTCCTGTCTCCATCCACATGAAGTTGGCATTCTTG   120

Bur-0    ------------------------------------------------------------   116
Col-0    TGTCTTTACGGCCTAAGTTTGCGTTTCTACCTCTTGAGAAATGAATCTTGCCTTCTTGTT   180
Got-7    TGTCTTTACGGCCTAAGTTTGCGTTTCTACCTCTTGAGAAATGAATC-TGCCTTCTTGTT   179
Cvi-0    TGTCTTTACGGCCTAAGTTTGCGTTTCTACCTCTTGAGAAATGAATCTTGCCTTCTTGTT   180
Ler-1    TGTCTTTACGGCCTAAGTTTGCGTTTCTACCTCTTGAGAAATGAATC-TGCCTTCTTGTT   179
Bay-0    TGTCTTTACGGCCTAAGTTTGCGTTTCTACCTCTTGAGAAATGAATCTTGCCTTCTTGTT   180

Bur-0    ---    116
Col-0    TAG    183
Got-7    TAG    182
Cvi-0    TAG    183
Ler-1    TAG    182
Bay-0    TAG    183
```

```
Bur-0    MIEMVNDN* RSGPRERSMYFWKVGHFEALPVSIHMKLAX
Col-0    MIEMVNDNWRSGPRERSMYFWKVGHFEALPVSIHMKLAFLCLYGLSLRFYLLRNESCLLV*
Got-7    MIEMVNDNWRSGPRERSMYFWKVGHFEALPVSIHMKLAFLCLYGLSLRFYLLRNESAFLFX
Cvi-0    MIEMVNDN* RSGPRERSMYFWKVGHFEALPVSIHMKLAFLCLYGLSLRFYLLRNESCLLV*
Ler-1    MIEMVNDN* RSGPRERSMYFWKVGHFEALPVSIHMKLAFLCLYGLSLRFYLLRNESAFLFX
Bay-0    MIEMVNDN* RSGPRERSMYFWKVGHFEALPVSIHMKLAFLCLYGLSLRFYLLRNESCLLV*
```

Arabidopsis lyrata lyrata (JGI draft) gene model produced using genewise

```
Col-0               1 MIEMVNDNWRSGPRERSMYFWKVGHFEALPVSIHMKLAFLCLYGLSLRF
                      MIEMVNDN RSGPRERSMYFWK      LPVSIHMK A+LC YGLSLRF
                      MIEMVNDNXRSGPRERSMYFWK!-----LPVSIHMKWALLC!YGLSLRF
A.lyrata lyrata    11 aagagagataagccgaaattta2     ccgtacaatgttt2tgcatct
                      ttattaaaggggcgaggtatga      tctctatagcttg agtgtgt
                      gtaggtttaaatgaggatgctga      ttacccgggaggt ccatgtt


Col-0              50 YLLRNESCLLV
                       LLRNESCLLV
                      !LLRNESCLLV
A.lyrata lyrata   141 2ctaagttccg
                       ttgaacgttt
                       cgatatcttt
```

Arabidopsis lyrata petraea plech 11-2 gene model produced using genewise

```
Col-0               1 MIEMVNDNWRSGPRERSMYFWKVGHFEALPVSIHMKLAFLCLYGLSLRF
                      MIEMVNDN RSGPRERSMYFW       LPVSIHMK A+LC YGLSLRF
                      MIEMVNDNXRSGPRERSMYFW!-----xLPVSIHMKWALLC!YGLSLRF
plech 11-2         66 aagagagataagccgaaattt2     Nccgtacaatgttt2tgcatct
                      ttattaaaggggcgaggtatg       ctctctatagcttg agtgtgt
                      gtaggtttaaatgaggatgctg      tttacccgggaggt ccatgtt


Col-0              50 YLLRNESCLLV
                       LLRNESCLLV
                      !LLRNESCLLV
plech 11-2        196 2ctaagttccg
                       ttgaacgttt
                       cgatatcttt
```

AT2G46567.1

```
Col-0         ATGATTTGGAGAGTTGGGAACAGAGACATTAGTGATTATAAGGTGTTGGGAAAGCGTGAC   60
Ler-1         ATGATTTGGAGAGTTGGGAACAGAGACATTAGTGATTATAAGGTGTTGGGAAAGCGTGAC   60
Bay-0         ATGATTTGGAGAGTTGGGAACTGAGACATTAGTGATTATAAGGTGTTGGGAAAGCGTGAC   60
Bur-0         ATGATTTGGAGAGTTGGGAACTGAGACATTAGTGATTATAAGGTGTTGGGAAAGCGTGAC   60
Got-7         ATGATTTGGAGAGTTGGGAACTGAGACATTAGTGATTATAAGGTGTTGGGAAAGCGTGAC   60
Cvi-0         ATGATTTGGAGAGTTGGGAACTGAGACATTAGTGATTATAAGGTGTTGGGAAAGCGTGAC   60
plech 9-7     ATGATTTGGAGAGTTGGTAACAGAGACATTAGTGATTATAAGGTGTTGGGAAGGCGTGAC   60
plech 17-7    ATGATTTGGAGAGTTGGTAACAGAGACATTAGTGATTATAAGGTGTTGGGAAGGCGTGAC   60
plech 13-4    ATGATTTGGAGAGTTGGTAACAGAGACATTAGTGATTATAAGGTGTTGGGAAGGCGTGAC   60
plech 8-3     ATGATTTGGAGAGTTGGTAACAGAGACATTAGTGATTATAAGGTGTTGGGAAGGCGTGAC   60
A.lyrata      ATGATTTGGAGAGTTGGTAACAGAGACATTAGTGATTATAAGGTGTTGGGAAGGCGTGAC   60
plech 14-5    ATGATTTGGAGAGTTGGTAACAGAGACATTAGTGATTATAAGGTGTTGGGAAGGCGTGAC   60
plech 11-2    ATGATTTGGAGAGTTGGTAACAGAGACATTAGTGATTATAAGGTGTTGGGAAGGCGTGAC   60

Col-0         TTGTTTCTTGACCCCAGCGTGAGATTGAAGGCAACACACGAGTTACTATAA   111
Ler-1         TTGTTTCTTGACCCCAGCGTGAGATTGAAGGCAACACACGAGTTACTATAA   111
Bay-0         TTGTTTCTTGACCCCAGCGTGAGATTGAAGGCAACACACGAGTTACTATAA   111
Bur-0         TTGTTTCTTGACCCCAGCGTGAGATTGAAGGCAACACACGAGTTACTATAA   111
Got-7         TTGTTTCTTGACCCCAGCGTGAGATTGAAGGCAACACACGAGTTACTATAA   111
Cvi-0         TTGTTTCTTGACCCCAGCGTGAGATTGAAGGCAACACACGAGTTACTATAA   111
plech 9-7     TTGTTTCTTGACCCCAGCGTGAGATTGAAGGCAACACACGAGTTACTATAA   111
plech 17-7    TTGTTTCTTGACCCCAGCGTGAGATTGAAGGCAACACACGAGTTACTATAA   111
plech 13-4    TTGTTTCTTGACCCCAGCGTGAGATTGAAGGCAACACACGAGTTACTATAA   111
plech 8-3     TTGTTTCTTGACCCCAGCGTGAGATTGAAGGCAACACACGAGTTACTATAA   111
A.lyrata      TTGTTTCTTGACCCCAGCGTGAGATTGAAGGCAACACACGAGTTACTATAA   111
plech 14-5    TTGTTTCTTGACCCCAGCGTGAGATTGAAGGCAACACACGAGTTACTATAA   111
plech 11-2    TTGTTTCTTGARCCCAGCGTGAGATTGAAGGCAACACACGAGTTACTATAA   111

Col-0         MIWRVGNRDISDYKVLGKRDLFLDPSVRLKATHELL*
Ler-1         MIWRVGNRDISDYKVLGKRDLFLDPSVRLKATHELL*
Bay-0         MIWRVGN*DISDYKVLGKRDLFLDPSVRLKATHELL*
Bur-0         MIWRVGN*DISDYKVLGKRDLFLDPSVRLKATHELL*
Got-7         MIWRVGN*DISDYKVLGKRDLFLDPSVRLKATHELL*
Cvi-0         MIWRVGN*DISDYKVLGKRDLFLDPSVRLKATHELL*
plech 9-7     MIWRVGNRDISDYKVLGRRDLFLDPSVRLKATHELL*
plech 17-7    MIWRVGNRDISDYKVLGRRDLFLDPSVRLKATHELL*
plech 13-4    MIWRVGNRDISDYKVLGRRDLFLDPSVRLKATHELL*
plech 8-3     MIWRVGNRDISDYKVLGRRDLFLDPSVRLKATHELL*
lyrata        MIWRVGNRDISDYKVLGRRDLFLDPSVRLKATHELL*
plech 14-5    MIWRVGNRDISDYKVLGRRDLFLDPSVRLKATHELL*
plech 11-2    MIWRVGNRDISDYKVLGRRDLFLEPSVRLKATHELL*
```

AT1G62181.1

```
Cvi-0        ATGCAAAAACTCAGCGCTTACGGTTACATCGCGTGGTTAGCATTAGTGTG   50
plech 8-3    CTGCAAAAACTCAGCGCTTACGGTTACATCGCGTGGTGAGCATTAGTGTG   50
plech 9-7    CTGCAAAAACTCAGCGCTTACGGTTACATCGCGTGGTGAGCATTAGTGTG   50
lyrata       CTGCAAAAACTCAGCGCTTACGGTTACATCGCGTGGTGAGCATTAGTGTG   50
Ler-1        ---CAAAAACTCAGCGCTTACGGTTACATCGCGTGGTGAGCATTAGTGTG   47
Got-7        ATGCAAAAACTCAGCGCTTACGGTTACATCGCGTGGTGAGCATTAGTGTG   50
Bur-0        ATGCAAAAACTCAGCGCTTACGGTTACATCGCGTGGTTAGCATTAGTGTG   50
Col-0        ATGCAAAAACTCAGCGCTTACGGTTACATCGCGTGGTTAGCATTAGTGTG   50
Bay-0        ATGCAAAAACTCAGCGCTTACGGTTACATCGCGTGGTTAGCATTAGTGTG   50

Cvi-0        TGAGATGTGGCGGCTAGGTAAGGAGGACACATGGCTGAGAACAAAACTTT   100
plech        TGAGATGTGGCGGCTAGGAAAGGAAGACACACGGCTGAGA----------   90
plech        TGAGATGTGGCGGCTAGGAAAGGAAGACACACGGCTGAGA----------   90
lyrata       TAAGATGTGGCGGCTAGGAAAGGAAGACACACGGCTGAGA----------   90
Ler-1        TGAGATGTGGCGGCTAGGA-AGGAGGGCACACGGCTGAGAACAAAACTTT   96
Got-7        TGAGATGTGGCGGCTAGGAGAGGAGGACACACGGCTGAGAAGAAAACTTT   100
Bur-0        TGAGATGTGGCGGCTAGGAGAGGAGGACACACGGCTGAGAACAAAACTTT   100
Col-0        TGAGATGTGGCGGCTAGGAGAGGAGGACACACGGCTGAGAAGAAAACTTT   100
Bay-0        TGAGATGTGGCGGCTAGGAGAGGAGGACACACGGCTGAGAAGAAAACTTT   100

Cvi-0        GA      102
plech 8-3    --      90
plech 9-7    --      90
lyrata       --      90
Ler-1        GA      98
Got-7        GA      102
Bur-0        GA      102
Col-0        GA      102
Bay-0        GA      102

Cvi-0        MQKLSAYGYIAWLALVCEMWRLGKEDTWLRTKL*
plech 8-3     LQKLSAYGYIAW*ALVCEMWRLGKEDTR LR
plech 9-7     LQKLSAYGYIAW*ALVCEMWRLGKEDTR LR
lyrata        LQKLSAYGYIAW*ALVCKMWRLGKEDTR LR
Ler-1         -QKLSAYGYIAW*ALVCEMWRLGRRAHG *EQNFX
Got-7        MQKLSAYGYIAW*ALVCEMWRLGEEDTR LRRKL*
Bur-0        MQKLSAYGYIAWLALVCEMWRLGEEDTR LRTKL*
Col-0        MQKLSAYGYIAWLALVCEMWRLGEEDTR LRRKL*
Bay-0        MQKLSAYGYIAWLALVCEMWRLGEEDTR LRRKL*
```
