# Supplementary material for: Evolutionary origins of Brassicaceae specific genes in Arabidopsis thaliana
Source: BMC Evol Biol. 2011 Feb 18;11:47. doi: 10.1186/1471-2148-11-47 (PMC3049755; doi:10.1186/1471-2148-11-47)
Supplement: Additional file 12 — Summary of all stress responsive LSGs. Red = up-regulated genes, blue = down-regulated genes. For the stress conditions listed across the bottom of each table; blue = abiotic, green = biotic, purple = growth conditions, yellow = hormone treatment and red = chemical treatment. [file 1471-2148-11-47-S12.PDF]

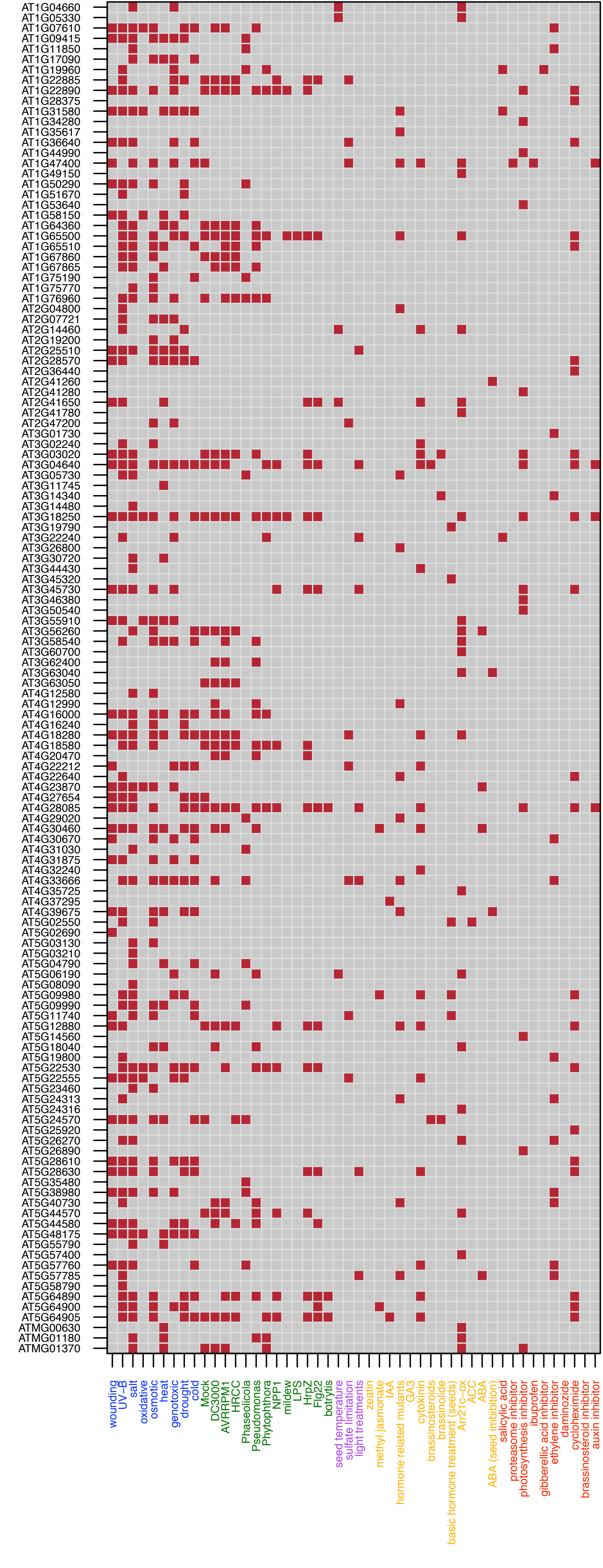
AT1G04660
AT1G05330
AT1G07610
AT1G09415
AT1G11850
AT1G17090
AT1G19960
AT1G22885
AT1G22890
AT1G28375
AT1G31580
AT1G34280
AT1G35617
AT1G36640
AT1G44990
AT1G47400
AT1G49150
AT1G50290
AT1G51670
AT1G53640
AT1G58150
AT1G64360
AT1G65500
AT1G65510
AT1G67860
AT1G67865
AT1G75190
AT1G75770
AT1G76960
AT2G04800
AT2G07721
AT2G14460
AT2G19200
AT2G25510
AT2G28570
AT2G36440
AT2G41260
AT2G41280
AT2G41650
AT2G41780
AT2G47200
AT3G01730
AT3G02240
AT3G03020
AT3G04640
AT3G05730
AT3G11745
AT3G14340
AT3G14480
AT3G18250
AT3G19790
AT3G22240
AT3G26800
AT3G30720
AT3G44430
AT3G45320
AT3G45730
AT3G46380
AT3G50540
AT3G55910
AT3G56260
AT3G58540
AT3G60700
AT3G62400
AT3G63040
AT3G63050
AT4G12580
AT4G12990
AT4G16000
AT4G16240
AT4G18280
AT4G18580
AT4G20470
AT4G22212
AT4G22640
AT4G23870
AT4G27654
AT4G28085
AT4G29020
AT4G30460
AT4G30670
AT4G31030
AT4G31875
AT4G32240
AT4G33666
AT4G35725
AT4G37295
AT4G39675
AT5G02550
AT5G02690
AT5G03130
AT5G03210
AT5G04790
AT5G06190
AT5G08090
AT5G09980
AT5G09990
AT5G11740
AT5G12880
AT5G14560
AT5G18040
AT5G19800
AT5G22530
AT5G22555
AT5G23460
AT5G24313
AT5G24316
AT5G24570
AT5G25920
AT5G26270
AT5G26890
AT5G28610
AT5G28630
AT5G35480
AT5G38980
AT5G40730
AT5G44570
AT5G44580
AT5G48175
AT5G55790
AT5G57400
AT5G57760
AT5G57785
AT5G58790
AT5G64890
AT5G64900
AT5G64905
ATMG00630
ATMG01180
ATMG01370
wounding
UV-B
salt
oxidative
osmotic
heat
genotoxic
drought
cold
Mock
DC3000
AVRRPM1
HRCC
Phaseolicola
Pseudomonas
Phytophthora
NPP1
mildew
LPS
HrpZ
Flg22
botrytis
seed temperature
sulfate limitation
light treatments
zeatin
methyl jasmonate
IAA
hormone related mutants
GA3
cytokinin
brassinosteroids
brassinolide
basic hormone treatment (seeds)
Arr21c-ox
ACC
ABA
ABA (seed imbibition)
salicylic acid
proteasome inhibitor
photosynthesis inhibitor
ibuprofen
gibberellic acid inhibitor
ethylene inhibitor
daminozide
cycloheximide
brassinosteroid inhibitor
auxin inhibitor

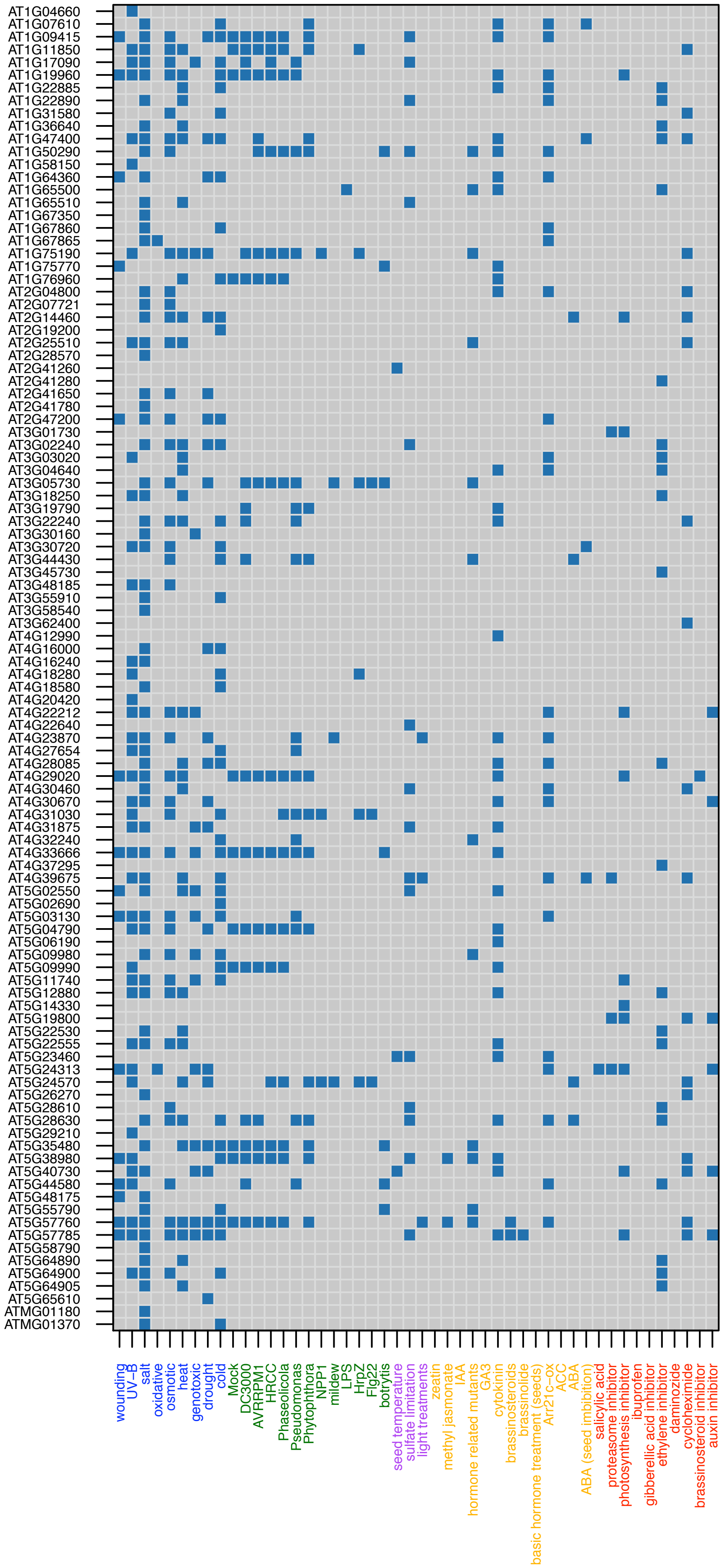
AT1G04660
AT1G07610
AT1G09415
AT1G11850
AT1G17090
AT1G19960
AT1G22885
AT1G22890
AT1G31580
AT1G36640
AT1G47400
AT1G50290
AT1G58150
AT1G64360
AT1G65500
AT1G65510
AT1G67350
AT1G67860
AT1G67865
AT1G75190
AT1G75770
AT1G76960
AT2G04800
AT2G07721
AT2G14460
AT2G19200
AT2G25510
AT2G28570
AT2G41260
AT2G41280
AT2G41650
AT2G41780
AT2G47200
AT3G01730
AT3G02240
AT3G03020
AT3G04640
AT3G05730
AT3G18250
AT3G19790
AT3G22240
AT3G30160
AT3G30720
AT3G44430
AT3G45730
AT3G48185
AT3G55910
AT3G58540
AT3G62400
AT4G12990
AT4G16000
AT4G16240
AT4G18280
AT4G18580
AT4G20420
AT4G22212
AT4G22640
AT4G23870
AT4G27654
AT4G28085
AT4G29020
AT4G30460
AT4G30670
AT4G31030
AT4G31875
AT4G32240
AT4G33666
AT4G37295
AT4G39675
AT5G02550
AT5G02690
AT5G03130
AT5G04790
AT5G06190
AT5G09980
AT5G09990
AT5G11740
AT5G12880
AT5G14330
AT5G19800
AT5G22530
AT5G22555
AT5G23460
AT5G24313
AT5G24570
AT5G26270
AT5G28610
AT5G28630
AT5G29210
AT5G35480
AT5G38980
AT5G40730
AT5G44580
AT5G48175
AT5G55790
AT5G57760
AT5G57785
AT5G58790
AT5G64890
AT5G64900
AT5G64905
AT5G65610
ATMG01180
ATMG01370
wounding
UV-B
salt
oxidative
osmotic
heat
genotoxic
drought
cold
Mock
DC3000
AVRRPM1
HRCC
Phaseolicola
Pseudomonas
Phytophthora
NPP1
mildew
LPS
HrpZ
Flg22
botrytis
seed temperature
sulfate limitation
light treatments
zeatin
methyl jasmonate
IAA
hormone related mutants
GA3
cytokinin
brassinosteroids
brassinolide
basic hormone treatment (seeds)
Arr21c-ox
ACC
ABA
ABA (seed imbibition)
salicylic acid
proteasome inhibitor
photosynthesis inhibitor
ibuprofen
gibberellic acid inhibitor
ethylene inhibitor
daminozide
cycloheximide
brassinosteroid inhibitor
auxin inhibitor
