# Supplementary material for: Evolutionary origins of Brassicaceae specific genes in Arabidopsis thaliana
Source: BMC Evol Biol. 2011 Feb 18;11:47. doi: 10.1186/1471-2148-11-47 (PMC3049755; doi:10.1186/1471-2148-11-47)
Supplement: Additional file 13 — Heatmaps indicating fold change of LSGs expressed under abiotic and biotic stress conditions. Genes highlighted with a p-value indicate significant differential expression. Colour bars at the top of columns indicate an enrichment of LSGs differentially expressed: red = up-regulated, blue = down-regulated, yellow = LSGs are enriched for both up and down-regulated genes. [file 1471-2148-11-47-S13.PDF]

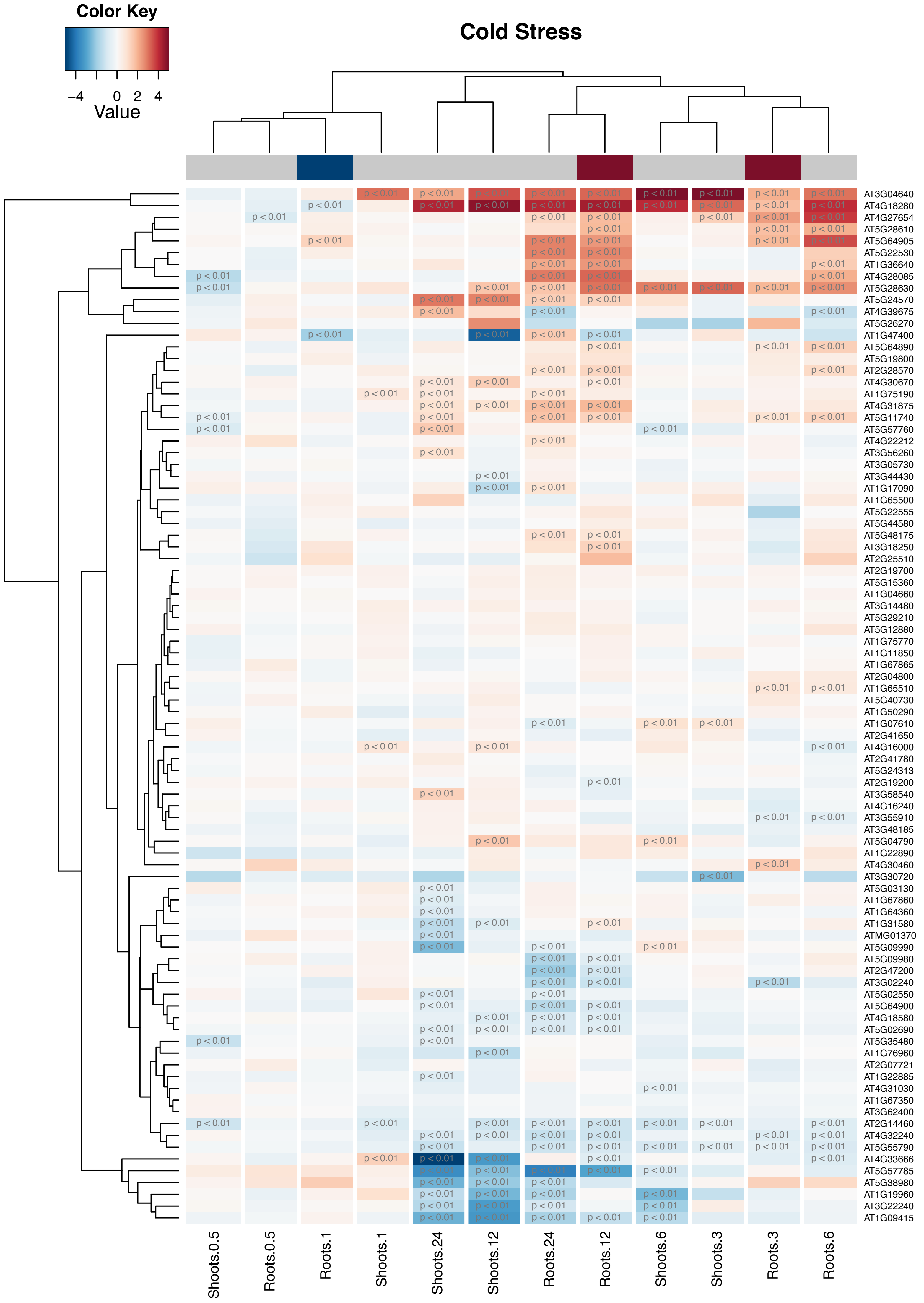
Color Key
−4
0
2
4
Value
Cold Stress
p < 0.01
AT3G04640
AT4G18280
AT4G27654
AT5G28610
AT5G64905
AT5G22530
AT1G36640
AT4G28085
AT5G28630
AT5G24570
AT4G39675
AT5G26270
AT1G47400
AT5G64890
AT5G19800
AT2G28570
AT4G30670
AT1G75190
AT4G31875
AT5G11740
AT5G57760
AT4G22212
AT3G56260
AT3G05730
AT3G44430
AT1G17090
AT1G65500
AT5G22555
AT5G44580
AT5G48175
AT3G18250
AT2G25510
AT2G19700
AT5G15360
AT1G04660
AT3G14480
AT5G29210
AT5G12880
AT1G75770
AT1G11850
AT1G67865
AT2G04800
AT1G65510
AT5G40730
AT1G50290
AT1G07610
AT2G41650
AT4G16000
AT2G41780
AT5G24313
AT2G19200
AT3G58540
AT4G16240
AT3G55910
AT3G48185
AT5G04790
AT1G22890
AT4G30460
AT3G30720
AT5G03130
AT1G67860
AT1G64360
AT1G31580
ATMG01370
AT5G09990
AT5G09980
AT2G47200
AT3G02240
AT5G02550
AT5G64900
AT4G18580
AT5G02690
AT5G35480
AT1G76960
AT2G07721
AT1G22885
AT4G31030
AT1G67350
AT3G62400
AT2G14460
AT4G32240
AT5G55790
AT4G33666
AT5G57785
AT5G38980
AT1G19960
AT3G22240
AT1G09415
Shoots.0.5
Roots.0.5
Roots.1
Shoots.1
Shoots.24
Shoots.12
Roots.24
Roots.12
Shoots.6
Shoots.3
Roots.3
Roots.6

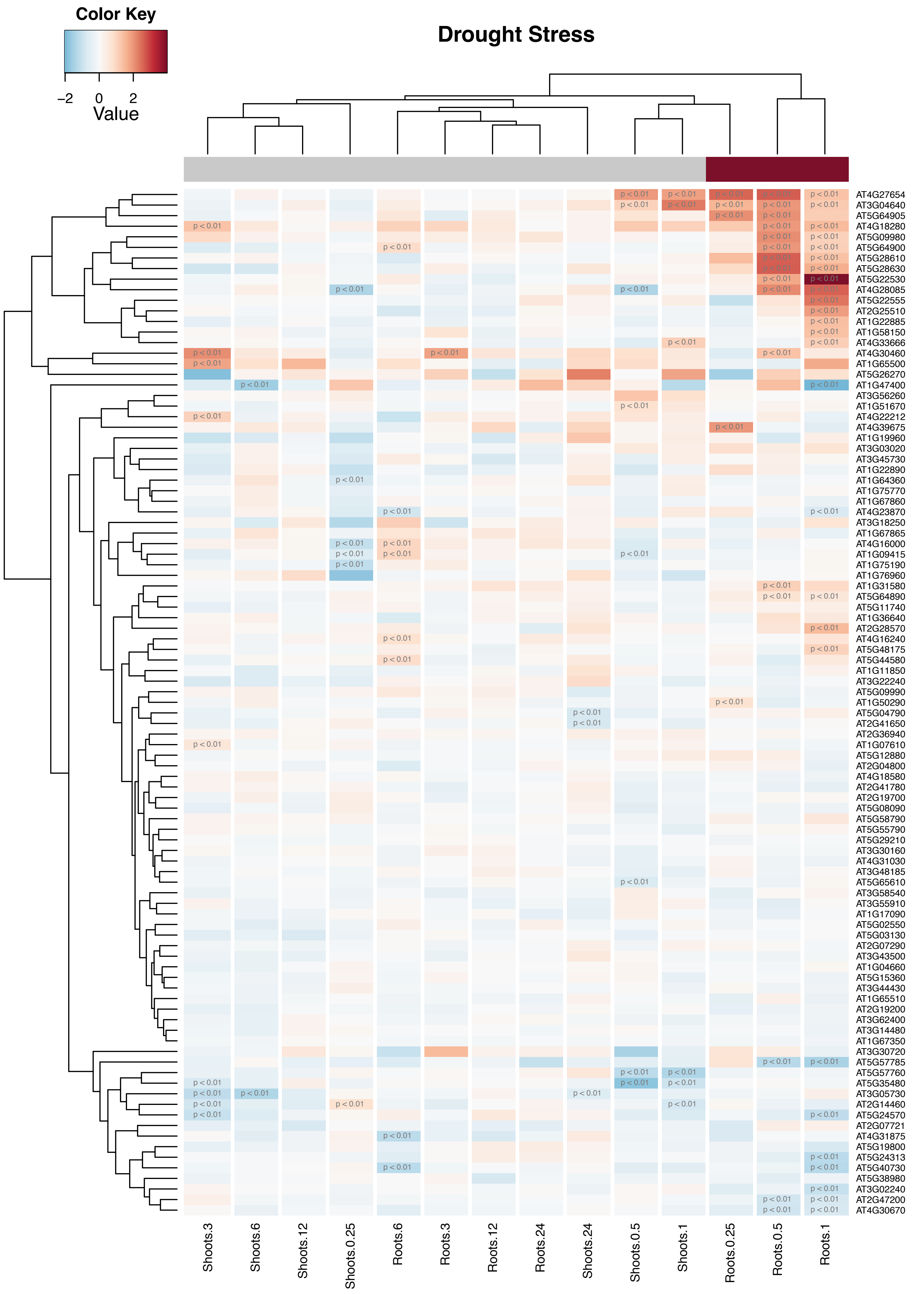
Color Key
−2
0
2
Value
Drought Stress
p < 0.01
AT4G27654
AT3G04640
AT5G64905
AT4G18280
AT5G09980
AT5G64900
AT5G28610
AT5G28630
AT5G22530
AT4G28085
AT5G22555
AT2G25510
AT1G22885
AT1G58150
AT4G33666
AT4G30460
AT1G65500
AT5G26270
AT1G47400
AT3G56260
AT1G51670
AT4G22212
AT4G39675
AT1G19960
AT3G03020
AT3G45730
AT1G22890
AT1G64360
AT1G75770
AT1G67860
AT4G23870
AT3G18250
AT1G67865
AT4G16000
AT1G09415
AT1G75190
AT1G76960
AT1G31580
AT5G64890
AT5G11740
AT1G36640
AT2G28570
AT4G16240
AT5G48175
AT5G44580
AT1G11850
AT3G22240
AT5G09990
AT1G50290
AT5G04790
AT2G41650
AT2G36940
AT1G07610
AT5G12880
AT2G04800
AT4G18580
AT2G41780
AT2G19700
AT5G08090
AT5G58790
AT5G55790
AT5G29210
AT3G30160
AT4G31030
AT3G48185
AT5G65610
AT3G58540
AT3G55910
AT1G17090
AT5G02550
AT5G03130
AT2G07290
AT3G43500
AT1G04660
AT5G15360
AT3G44430
AT1G65510
AT2G19200
AT3G62400
AT3G14480
AT1G67350
AT3G30720
AT5G57785
AT5G57760
AT5G35480
AT3G05730
AT2G14460
AT5G24570
AT2G07721
AT4G31875
AT5G19800
AT5G24313
AT5G40730
AT5G38980
AT3G02240
AT2G47200
AT4G30670
Shoots.3
Shoots.6
Shoots.12
Shoots.0.25
Roots.6
Roots.3
Roots.12
Roots.24
Shoots.24
Shoots.0.5
Shoots.1
Roots.0.25
Roots.0.5
Roots.1

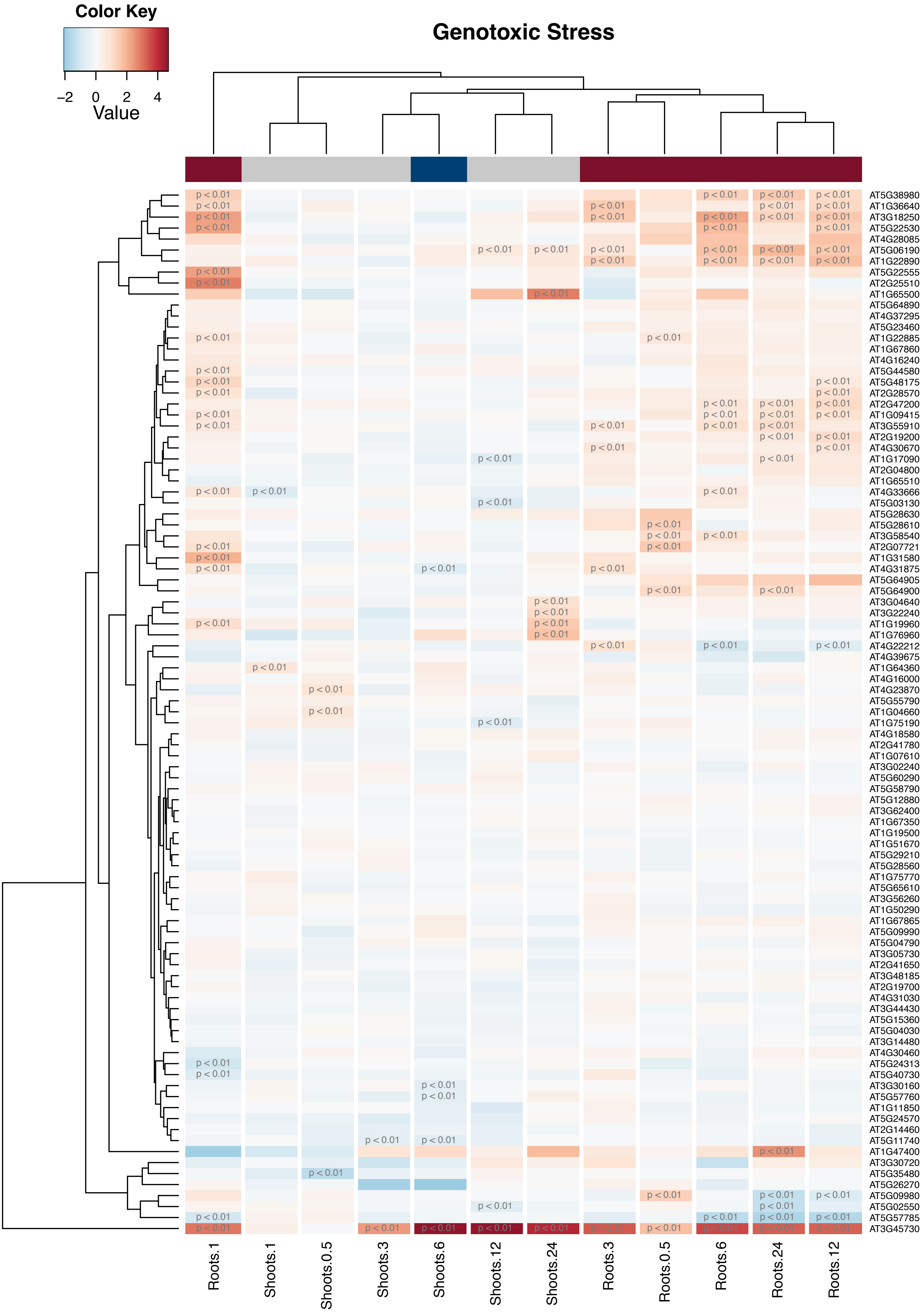

Color Key
−2 0 2 4
Value
Genotoxic Stress
p < 0.01
AT5G38980
AT1G36640
AT3G18250
AT5G22530
AT4G28085
AT5G06190
AT1G22890
AT5G22555
AT2G25510
AT1G65500
AT5G64890
AT4G37295
AT5G23460
AT1G22885
AT1G67860
AT4G16240
AT5G44580
AT5G48175
AT2G28570
AT2G47200
AT1G09415
AT3G55910
AT2G19200
AT4G30670
AT1G17090
AT2G04800
AT1G65510
AT4G33666
AT5G03130
AT5G28630
AT5G28610
AT3G58540
AT2G07721
AT1G31580
AT4G31875
AT5G64905
AT5G64900
AT3G04640
AT3G22240
AT1G19960
AT1G76960
AT4G22212
AT4G39675
AT1G64360
AT4G16000
AT4G23870
AT5G55790
AT1G04660
AT1G75190
AT4G18580
AT2G41780
AT1G07610
AT3G02240
AT5G60290
AT5G58790
AT5G12880
AT3G62400
AT1G67350
AT1G19500
AT1G51670
AT5G29210
AT5G28560
AT1G75770
AT5G65610
AT3G56260
AT1G50290
AT1G67865
AT5G09990
AT5G04790
AT3G05730
AT2G41650
AT3G48185
AT2G19700
AT4G31030
AT3G44430
AT5G15360
AT5G04030
AT3G14480
AT4G30460
AT5G24313
AT5G40730
AT3G30160
AT5G57760
AT1G11850
AT5G24570
AT2G14460
AT5G11740
AT1G47400
AT3G30720
AT5G35480
AT5G26270
AT5G09980
AT5G02550
AT5G57785
AT3G45730
Roots.1
Shoots.1
Shoots.0.5
Shoots.3
Shoots.6
Shoots.12
Shoots.24
Roots.3
Roots.0.5
Roots.6
Roots.24
Roots.12

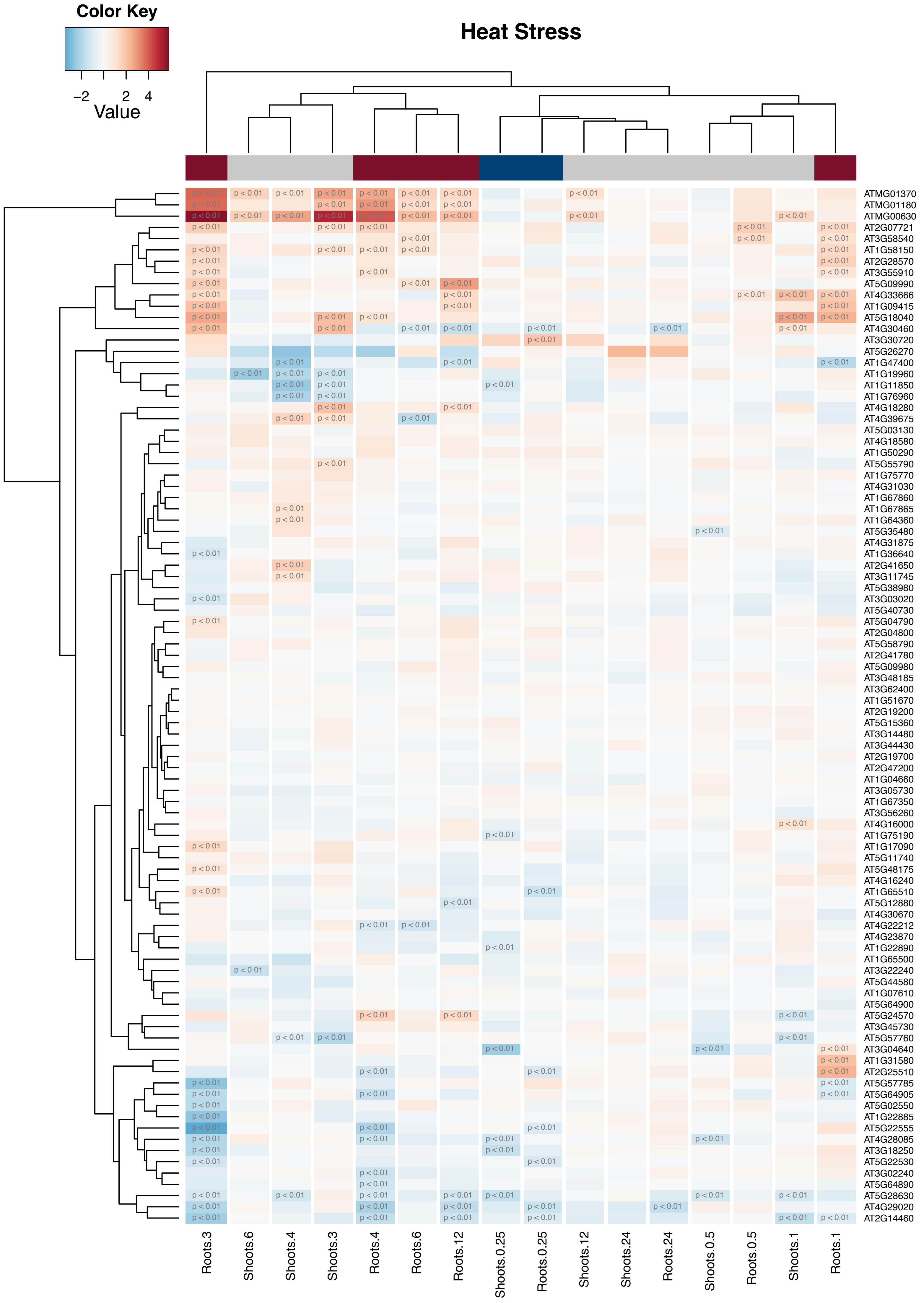

Color Key
−2
2
4
Value
Heat Stress
ATMG01370
ATMG01180
ATMG00630
AT2G07721
AT3G58540
AT1G58150
AT2G28570
AT3G55910
AT5G09990
AT4G33666
AT1G09415
AT5G18040
AT4G30460
AT3G30720
AT5G26270
AT1G47400
AT1G19960
AT1G11850
AT1G76960
AT4G18280
AT4G39675
AT5G03130
AT4G18580
AT1G50290
AT5G55790
AT1G75770
AT4G31030
AT1G67860
AT1G67865
AT1G64360
AT5G35480
AT4G31875
AT1G36640
AT2G41650
AT3G11745
AT5G38980
AT3G03020
AT5G40730
AT5G04790
AT2G04800
AT5G58790
AT2G41780
AT5G09980
AT3G48185
AT3G62400
AT1G51670
AT2G19200
AT5G15360
AT3G14480
AT3G44430
AT2G19700
AT2G47200
AT1G04660
AT3G05730
AT1G67350
AT3G56260
AT4G16000
AT1G75190
AT1G17090
AT5G11740
AT5G48175
AT4G16240
AT1G65510
AT5G12880
AT4G30670
AT4G22212
AT4G23870
AT1G22890
AT1G65500
AT3G22240
AT5G44580
AT1G07610
AT5G64900
AT5G24570
AT3G45730
AT5G57760
AT3G04640
AT1G31580
AT2G25510
AT5G57785
AT5G64905
AT5G02550
AT1G22885
AT5G22555
AT4G28085
AT3G18250
AT5G22530
AT3G02240
AT5G64890
AT5G28630
AT4G29020
AT2G14460
Roots.3
Shoots.6
Shoots.4
Shoots.3
Roots.4
Roots.6
Roots.12
Shoots.0.25
Roots.0.25
Shoots.12
Shoots.24
Roots.24
Shoots.0.5
Roots.0.5
Shoots.1
Roots.1

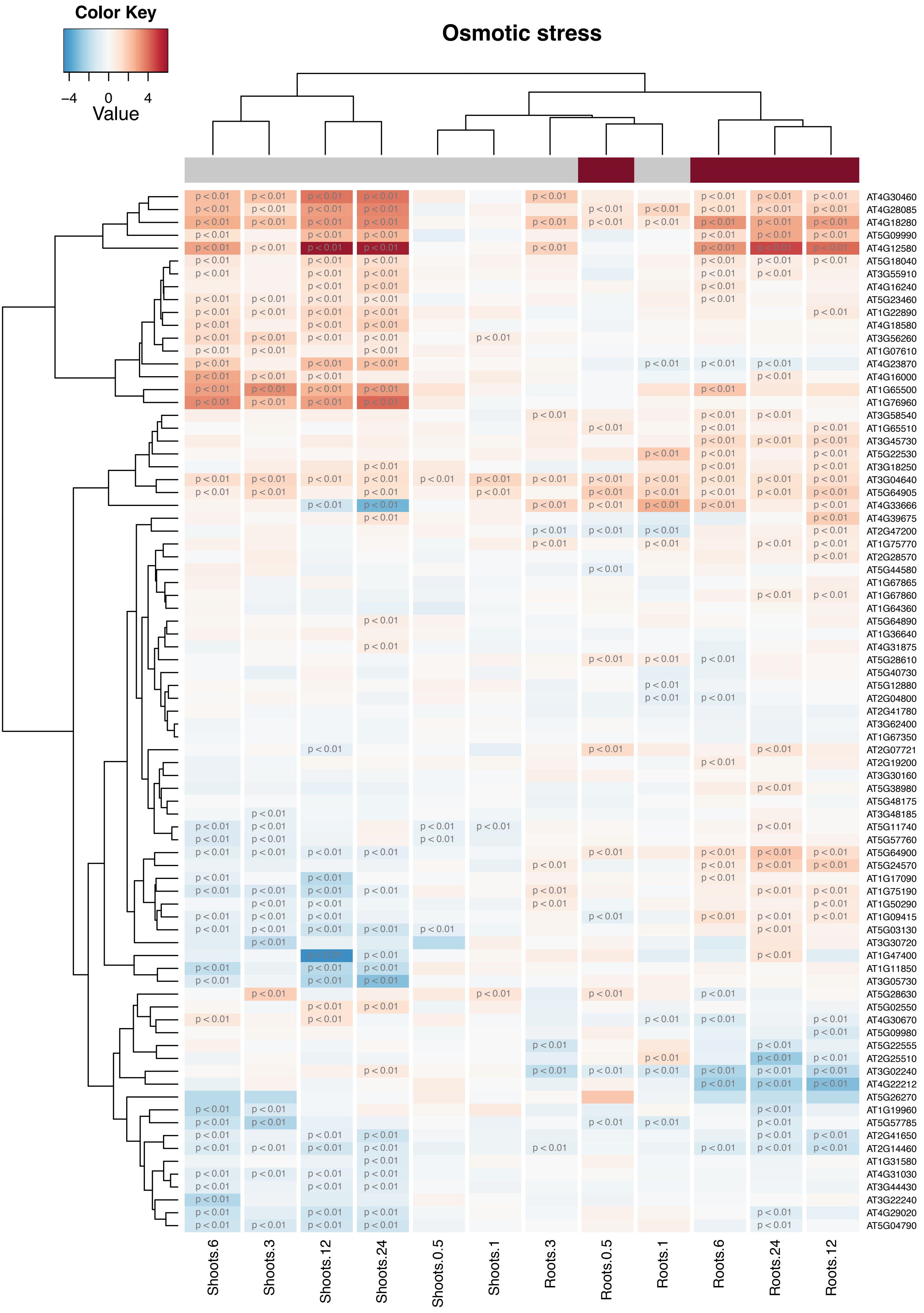

Osmotic stress
Color Key
−4 0 4
Value
AT4G30460
AT4G28085
AT4G18280
AT5G09990
AT4G12580
AT5G18040
AT3G55910
AT4G16240
AT5G23460
AT1G22890
AT4G18580
AT3G56260
AT1G07610
AT4G23870
AT4G16000
AT1G65500
AT1G76960
AT3G58540
AT1G65510
AT3G45730
AT5G22530
AT3G18250
AT3G04640
AT5G64905
AT4G33666
AT4G39675
AT2G47200
AT1G75770
AT2G28570
AT5G44580
AT1G67865
AT1G67860
AT1G64360
AT5G64890
AT1G36640
AT4G31875
AT5G28610
AT5G40730
AT5G12880
AT2G04800
AT2G41780
AT3G62400
AT1G67350
AT2G07721
AT2G19200
AT3G30160
AT5G38980
AT5G48175
AT3G48185
AT5G11740
AT5G57760
AT5G64900
AT5G24570
AT1G17090
AT1G75190
AT1G50290
AT1G09415
AT5G03130
AT3G30720
AT1G47400
AT1G11850
AT3G05730
AT5G28630
AT5G02550
AT4G30670
AT5G09980
AT5G22555
AT2G25510
AT3G02240
AT4G22212
AT5G26270
AT1G19960
AT5G57785
AT2G41650
AT2G14460
AT1G31580
AT4G31030
AT3G44430
AT3G22240
AT4G29020
AT5G04790
Shoots.6
Shoots.3
Shoots.12
Shoots.24
Shoots.0.5
Shoots.1
Roots.3
Roots.0.5
Roots.1
Roots.6
Roots.24
Roots.12

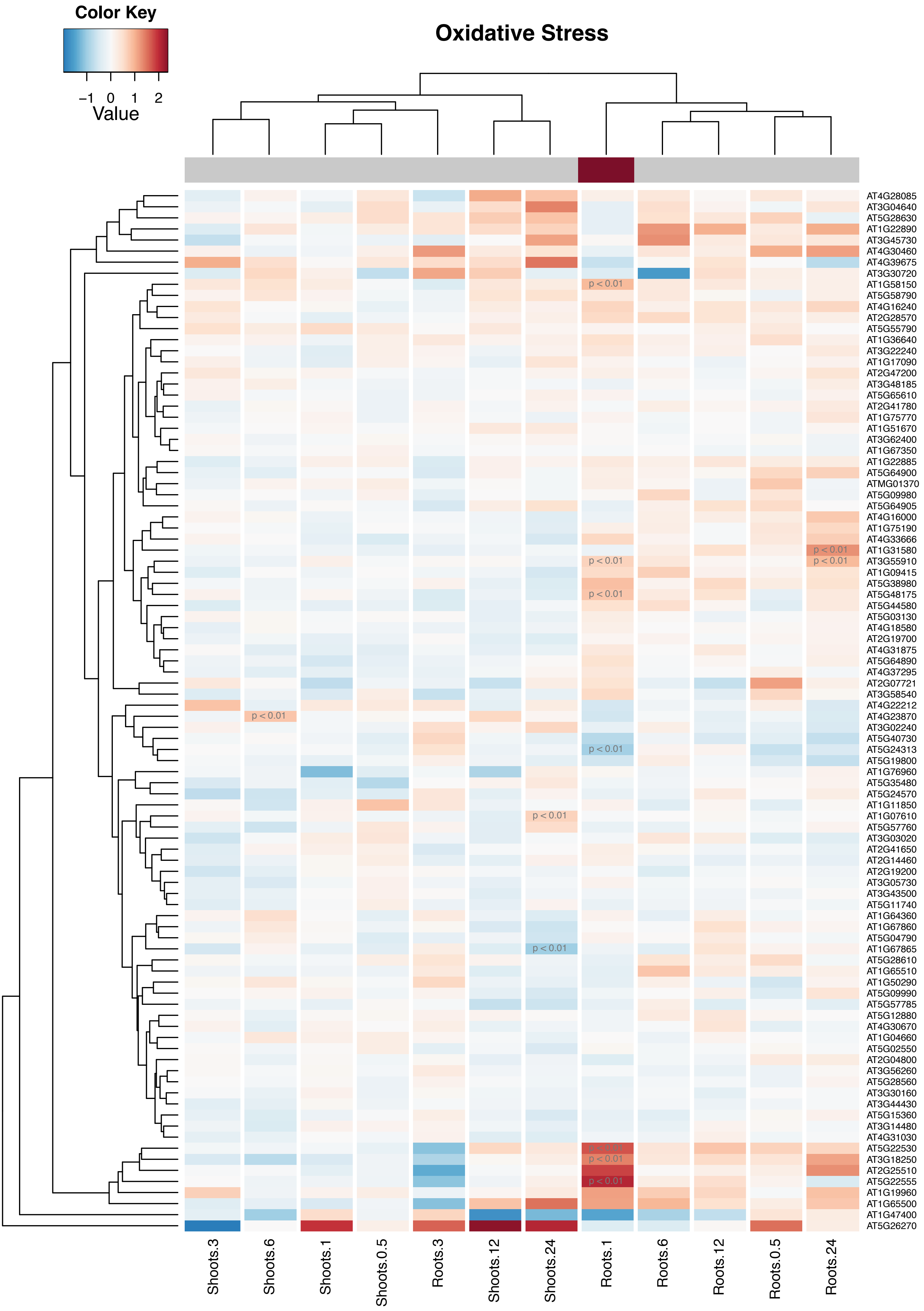
Color Key
−1 0 1 2
Value
Oxidative Stress
AT4G28085
AT3G04640
AT5G28630
AT1G22890
AT3G45730
AT4G30460
AT4G39675
AT3G30720
AT1G58150
AT5G58790
AT4G16240
AT2G28570
AT5G55790
AT1G36640
AT3G22240
AT1G17090
AT2G47200
AT3G48185
AT5G65610
AT2G41780
AT1G75770
AT1G51670
AT3G62400
AT1G67350
AT1G22885
AT5G64900
ATMG01370
AT5G09980
AT5G64905
AT4G16000
AT1G75190
AT4G33666
AT1G31580
AT3G55910
AT1G09415
AT5G38980
AT5G48175
AT5G44580
AT5G03130
AT4G18580
AT2G19700
AT4G31875
AT5G64890
AT4G37295
AT2G07721
AT3G58540
AT4G22212
AT4G23870
AT3G02240
AT5G40730
AT5G24313
AT5G19800
AT1G76960
AT5G35480
AT5G24570
AT1G11850
AT1G07610
AT5G57760
AT3G03020
AT2G41650
AT2G14460
AT2G19200
AT3G05730
AT3G43500
AT5G11740
AT1G64360
AT1G67860
AT5G04790
AT1G67865
AT5G28610
AT1G65510
AT1G50290
AT5G09990
AT5G57785
AT5G12880
AT4G30670
AT1G04660
AT5G02550
AT2G04800
AT3G56260
AT5G28560
AT3G30160
AT3G44430
AT5G15360
AT3G14480
AT4G31030
AT5G22530
AT3G18250
AT2G25510
AT5G22555
AT1G19960
AT1G65500
AT1G47400
AT5G26270
p < 0.01
Shoots.3
Shoots.6
Shoots.1
Shoots.0.5
Roots.3
Shoots.12
Shoots.24
Roots.1
Roots.6
Roots.12
Roots.0.5
Roots.24

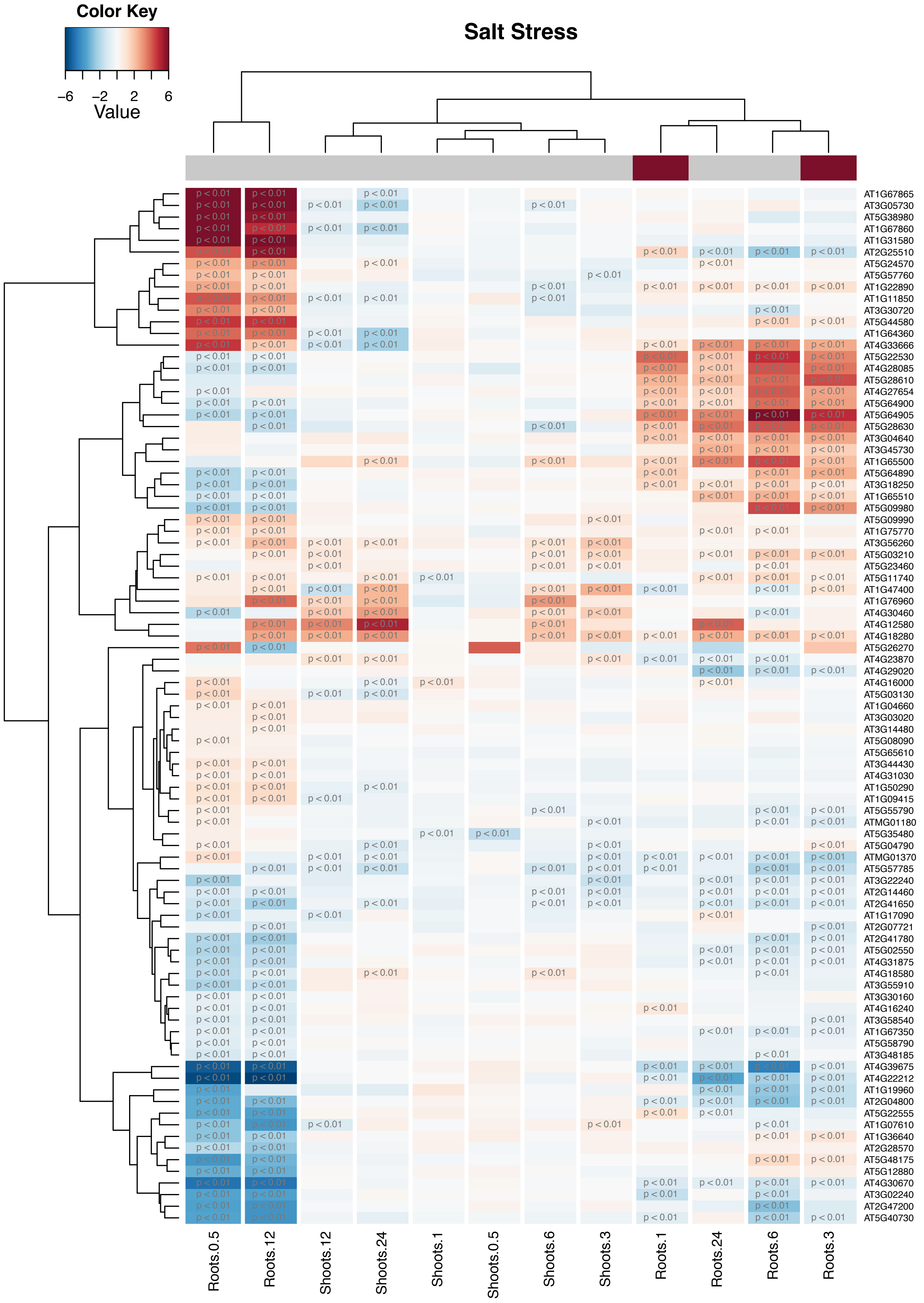

Color Key
−6 −2 2 6
Value
Salt Stress
p < 0.01
AT1G67865
AT3G05730
AT5G38980
AT1G67860
AT1G31580
AT2G25510
AT5G24570
AT5G57760
AT1G22890
AT1G11850
AT3G30720
AT5G44580
AT1G64360
AT4G33666
AT5G22530
AT4G28085
AT5G28610
AT4G27654
AT5G64900
AT5G64905
AT5G28630
AT3G04640
AT3G45730
AT1G65500
AT5G64890
AT3G18250
AT1G65510
AT5G09980
AT5G09990
AT1G75770
AT3G56260
AT5G03210
AT5G23460
AT5G11740
AT1G47400
AT1G76960
AT4G30460
AT4G12580
AT4G18280
AT5G26270
AT4G23870
AT4G29020
AT4G16000
AT5G03130
AT1G04660
AT3G03020
AT3G14480
AT5G08090
AT5G65610
AT3G44430
AT4G31030
AT1G50290
AT1G09415
AT5G55790
ATMG01180
AT5G35480
AT5G04790
ATMG01370
AT5G57785
AT3G22240
AT2G14460
AT2G41650
AT1G17090
AT2G07721
AT2G41780
AT5G02550
AT4G31875
AT4G18580
AT3G55910
AT3G30160
AT4G16240
AT3G58540
AT1G67350
AT5G58790
AT3G48185
AT4G39675
AT4G22212
AT1G19960
AT2G04800
AT5G22555
AT1G07610
AT1G36640
AT2G28570
AT5G48175
AT5G12880
AT4G30670
AT3G02240
AT2G47200
AT5G40730
Roots.0.5
Roots.12
Shoots.12
Shoots.24
Shoots.1
Shoots.0.5
Shoots.6
Shoots.3
Roots.1
Roots.24
Roots.6
Roots.3

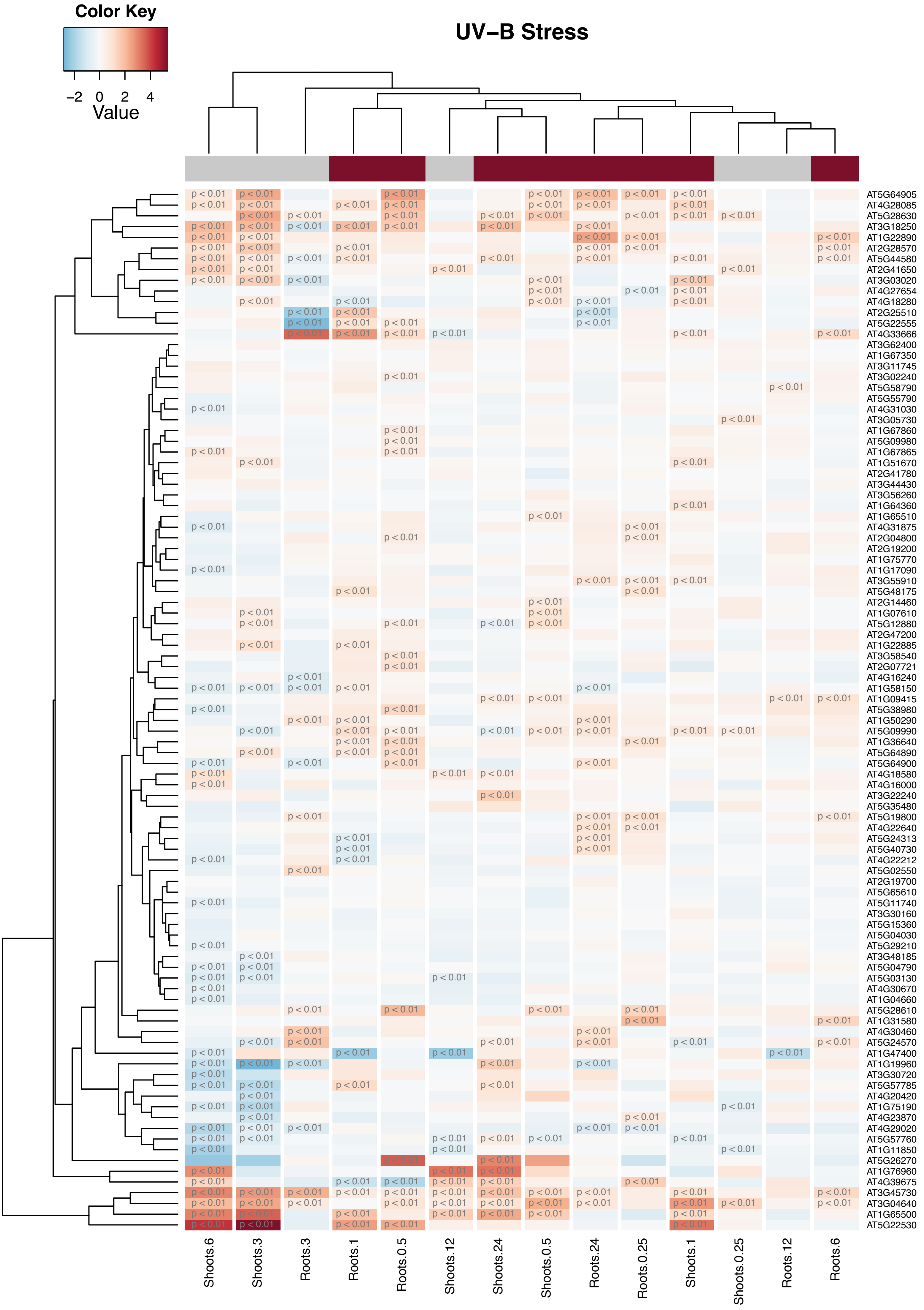

UV−B Stress
Color Key
−2 0 2 4
Value
AT5G64905
AT4G28085
AT5G28630
AT3G18250
AT1G22890
AT2G28570
AT5G44580
AT2G41650
AT3G03020
AT4G27654
AT4G18280
AT2G25510
AT5G22555
AT4G33666
AT3G62400
AT1G67350
AT3G11745
AT3G02240
AT5G58790
AT5G55790
AT4G31030
AT3G05730
AT1G67860
AT5G09980
AT1G67865
AT1G51670
AT2G41780
AT3G44430
AT3G56260
AT1G64360
AT1G65510
AT4G31875
AT2G04800
AT2G19200
AT1G75770
AT1G17090
AT3G55910
AT5G48175
AT2G14460
AT1G07610
AT5G12880
AT2G47200
AT1G22885
AT3G58540
AT2G07721
AT4G16240
AT1G58150
AT1G09415
AT5G38980
AT1G50290
AT5G09990
AT1G36640
AT5G64890
AT5G64900
AT4G18580
AT4G16000
AT3G22240
AT5G35480
AT5G19800
AT4G22640
AT5G24313
AT5G40730
AT4G22212
AT5G02550
AT2G19700
AT5G65610
AT5G11740
AT3G30160
AT5G15360
AT5G04030
AT5G29210
AT3G48185
AT5G04790
AT5G03130
AT4G30670
AT1G04660
AT5G28610
AT1G31580
AT4G30460
AT5G24570
AT1G47400
AT1G19960
AT3G30720
AT5G57785
AT4G20420
AT1G75190
AT4G23870
AT4G29020
AT5G57760
AT1G11850
AT5G26270
AT1G76960
AT4G39675
AT3G45730
AT3G04640
AT1G65500
AT5G22530
Shoots.6
Shoots.3
Roots.3
Roots.1
Roots.0.5
Shoots.12
Shoots.24
Shoots.0.5
Roots.24
Roots.0.25
Shoots.1
Shoots.0.25
Roots.12
Roots.6

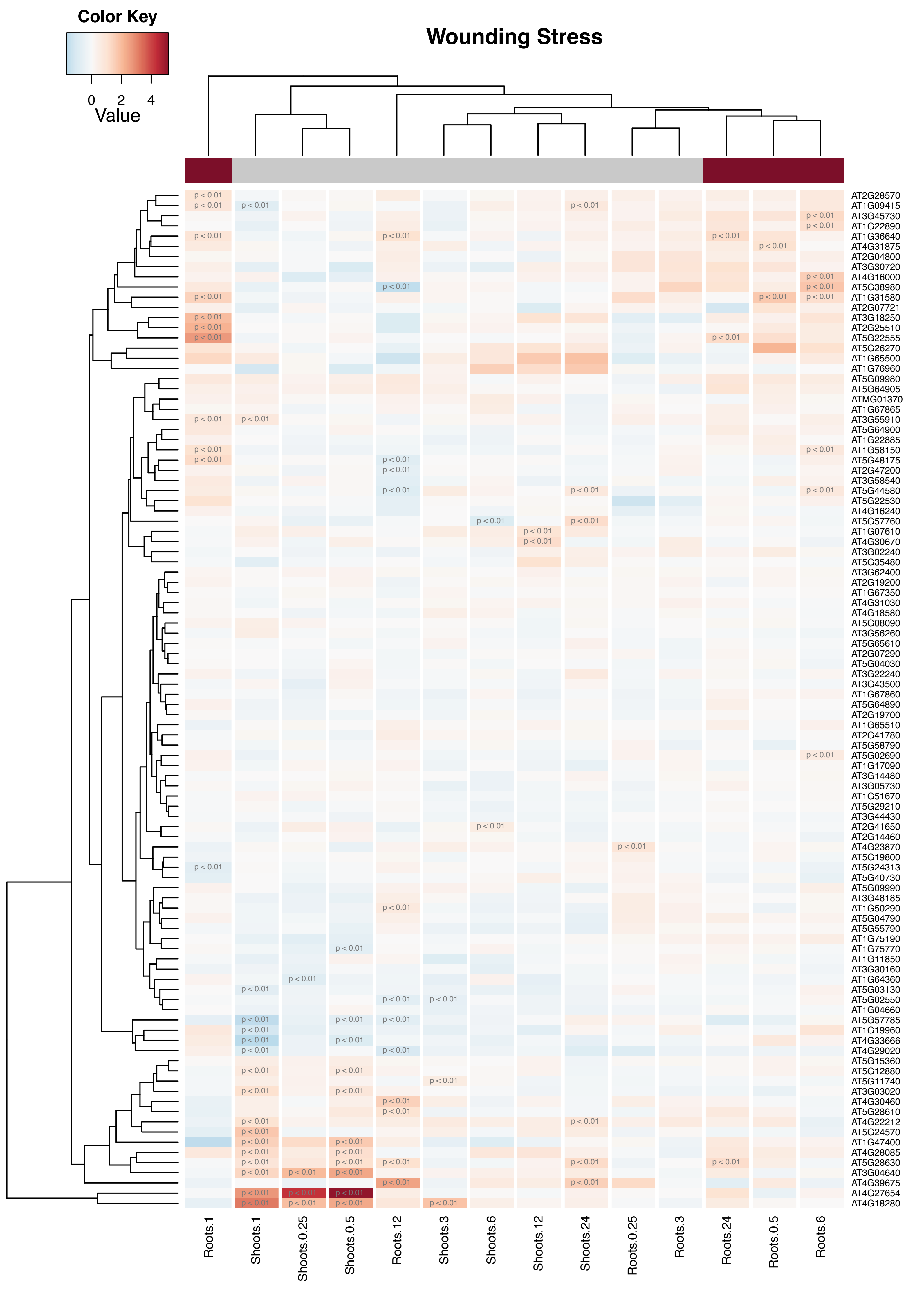
Color Key
0 2 4
Value
Wounding Stress
Roots.1
Shoots.1
Shoots.0.25
Shoots.0.5
Roots.12
Shoots.3
Shoots.6
Shoots.12
Shoots.24
Roots.0.25
Roots.3
Roots.24
Roots.0.5
Roots.6

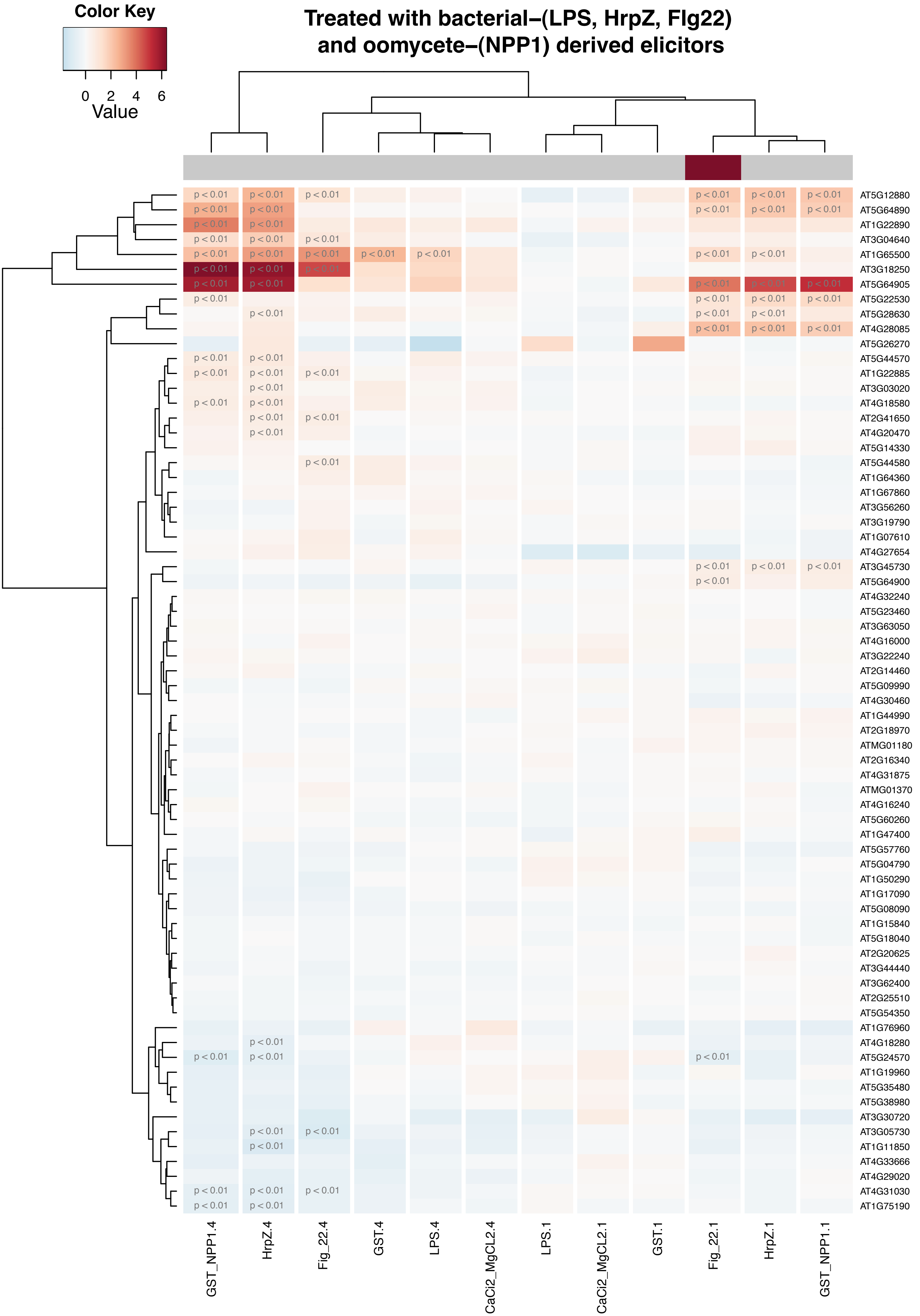
Treated with bacterial–(LPS, HrpZ, Flg22)
and oomycete–(NPP1) derived elicitors
Color Key
0 2 4 6
Value
p < 0.01
AT5G12880
AT5G64890
AT1G22890
AT3G04640
AT1G65500
AT3G18250
AT5G64905
AT5G22530
AT5G28630
AT4G28085
AT5G26270
AT5G44570
AT1G22885
AT3G03020
AT4G18580
AT2G41650
AT4G20470
AT5G14330
AT5G44580
AT1G64360
AT1G67860
AT3G56260
AT3G19790
AT1G07610
AT4G27654
AT3G45730
AT5G64900
AT4G32240
AT5G23460
AT3G63050
AT4G16000
AT3G22240
AT2G14460
AT5G09990
AT4G30460
AT1G44990
AT2G18970
ATMG01180
AT2G16340
AT4G31875
ATMG01370
AT4G16240
AT5G60260
AT1G47400
AT5G57760
AT5G04790
AT1G50290
AT1G17090
AT5G08090
AT1G15840
AT5G18040
AT2G20625
AT3G44440
AT3G62400
AT2G25510
AT5G54350
AT1G76960
AT4G18280
AT5G24570
AT1G19960
AT5G35480
AT5G38980
AT3G30720
AT3G05730
AT1G11850
AT4G33666
AT4G29020
AT4G31030
AT1G75190
GST_NPP1.4
HrpZ.4
Flg_22.4
GST.4
LPS.4
CaCl2_MgCL2.4
LPS.1
CaCl2_MgCL2.1
GST.1
Flg_22.1
HrpZ.1
GST_NPP1.1

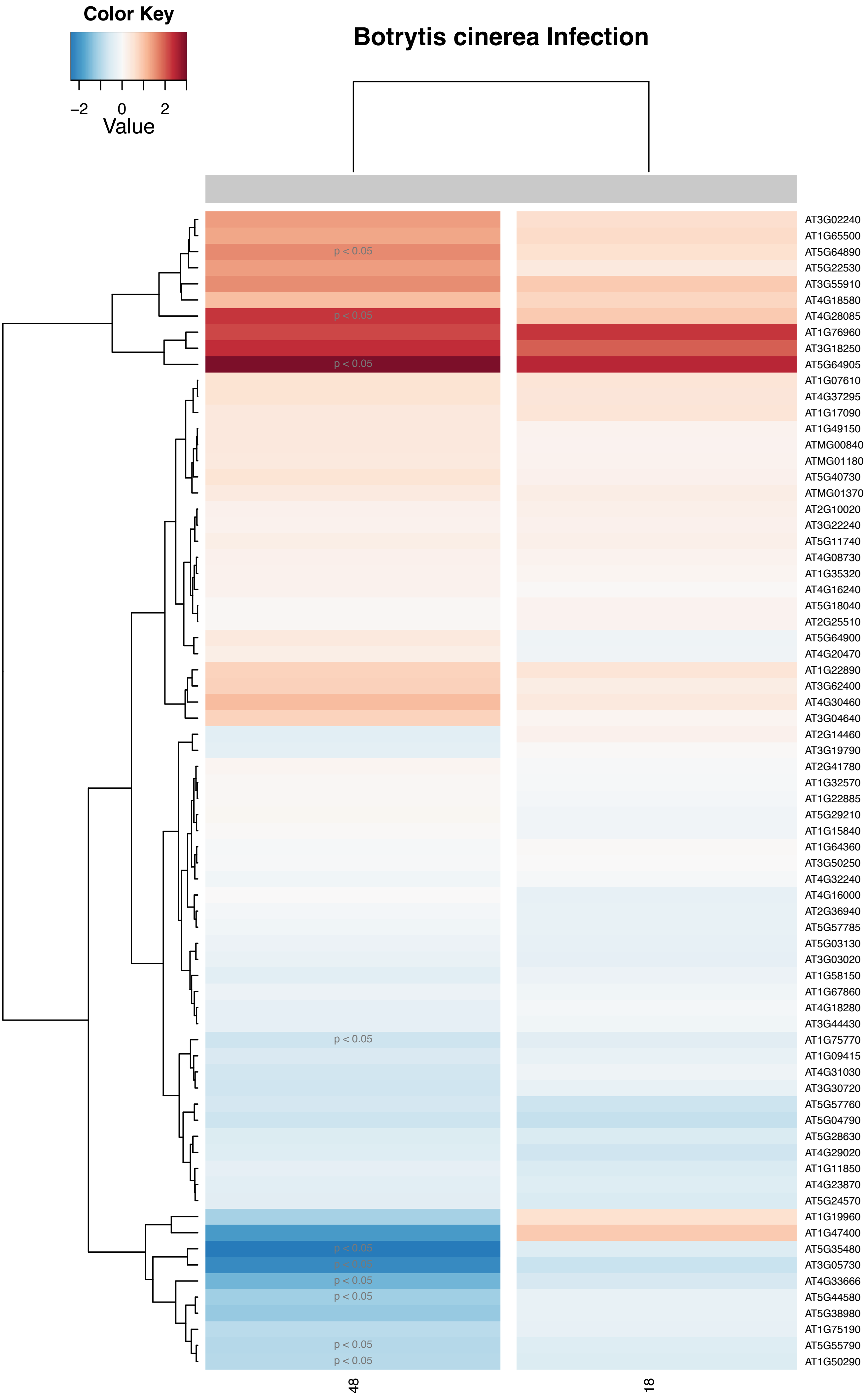
Color Key
−2 0 2
Value
Botrytis cinerea Infection
p < 0.05
AT3G02240
AT1G65500
AT5G64890
AT5G22530
AT3G55910
AT4G18580
AT4G28085
AT1G76960
AT3G18250
AT5G64905
AT1G07610
AT4G37295
AT1G17090
AT1G49150
ATMG00840
ATMG01180
AT5G40730
ATMG01370
AT2G10020
AT3G22240
AT5G11740
AT4G08730
AT1G35320
AT4G16240
AT5G18040
AT2G25510
AT5G64900
AT4G20470
AT1G22890
AT3G62400
AT4G30460
AT3G04640
AT2G14460
AT3G19790
AT2G41780
AT1G32570
AT1G22885
AT5G29210
AT1G15840
AT1G64360
AT3G50250
AT4G32240
AT4G16000
AT2G36940
AT5G57785
AT5G03130
AT3G03020
AT1G58150
AT1G67860
AT4G18280
AT3G44430
AT1G75770
AT1G09415
AT4G31030
AT3G30720
AT5G57760
AT5G04790
AT5G28630
AT4G29020
AT1G11850
AT4G23870
AT5G24570
AT1G19960
AT1G47400
AT5G35480
AT3G05730
AT4G33666
AT5G44580
AT5G38980
AT1G75190
AT5G55790
AT1G50290
48
18

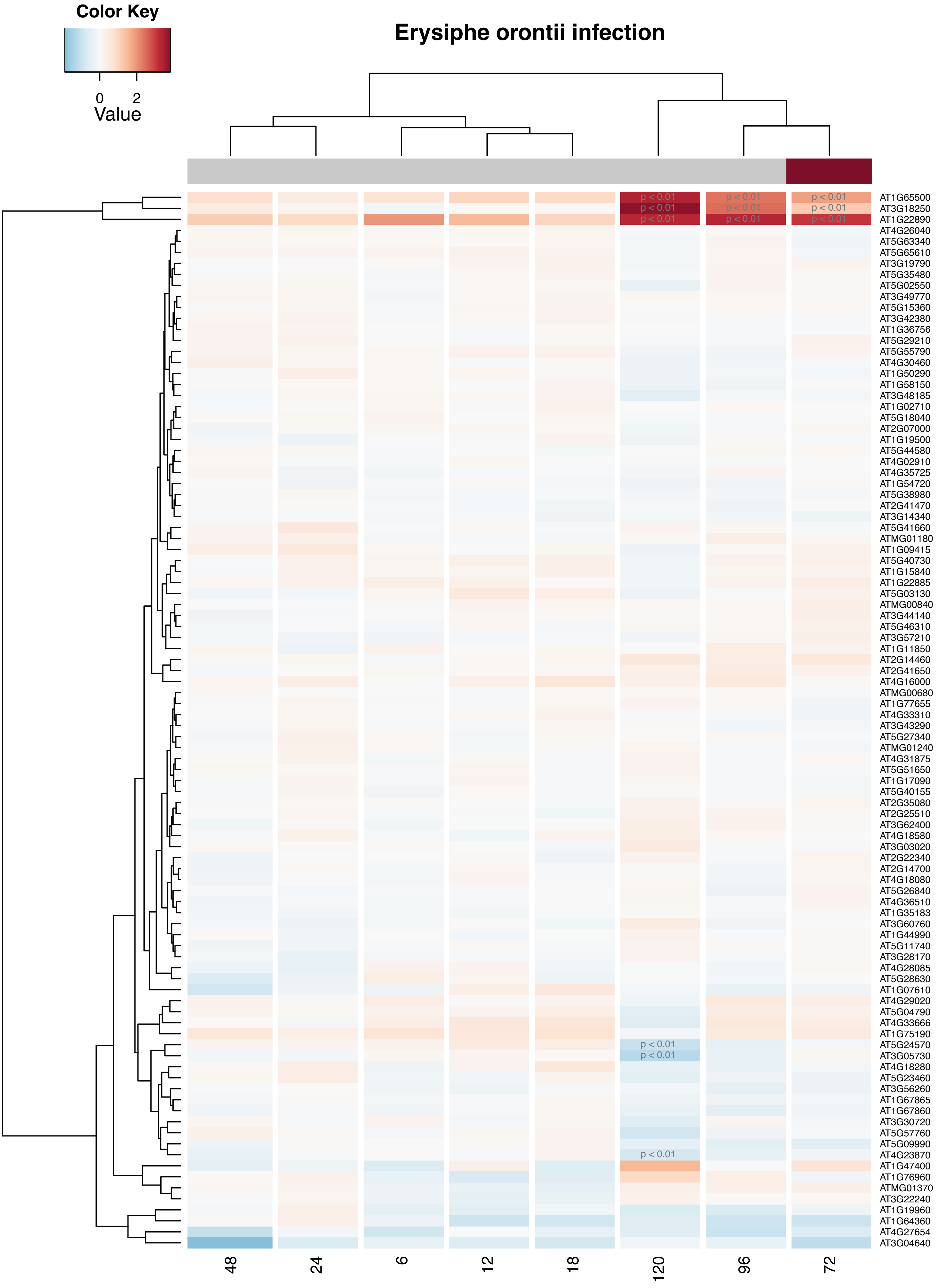

Color Key
0
2
Value
Erysiphe orontii infection
p < 0.01
AT1G65500
AT3G18250
AT1G22890
AT4G26040
AT5G63340
AT5G65610
AT3G19790
AT5G35480
AT5G02550
AT3G49770
AT5G15360
AT3G42380
AT1G36756
AT5G29210
AT5G55790
AT4G30460
AT1G50290
AT1G58150
AT3G48185
AT1G02710
AT5G18040
AT2G07000
AT1G19500
AT5G44580
AT4G02910
AT4G35725
AT1G54720
AT5G38980
AT2G41470
AT3G14340
AT5G41660
ATMG01180
AT1G09415
AT5G40730
AT1G15840
AT1G22885
AT5G03130
ATMG00840
AT3G44140
AT5G46310
AT3G57210
AT1G11850
AT2G14460
AT2G41650
AT4G16000
ATMG00680
AT1G77655
AT4G33310
AT3G43290
AT5G27340
ATMG01240
AT4G31875
AT5G51650
AT1G17090
AT5G40155
AT2G35080
AT2G25510
AT3G62400
AT4G18580
AT3G03020
AT2G22340
AT2G14700
AT4G18080
AT5G26840
AT4G36510
AT1G35183
AT3G60760
AT1G44990
AT5G11740
AT3G28170
AT4G28085
AT5G28630
AT1G07610
AT4G29020
AT5G04790
AT4G33666
AT1G75190
AT5G24570
AT3G05730
AT4G18280
AT5G23460
AT3G56260
AT1G67865
AT1G67860
AT3G30720
AT5G57760
AT5G09990
AT4G23870
AT1G47400
AT1G76960
ATMG01370
AT3G22240
AT1G19960
AT1G64360
AT4G27654
AT3G04640
48
24
6
12
18
120
96
72

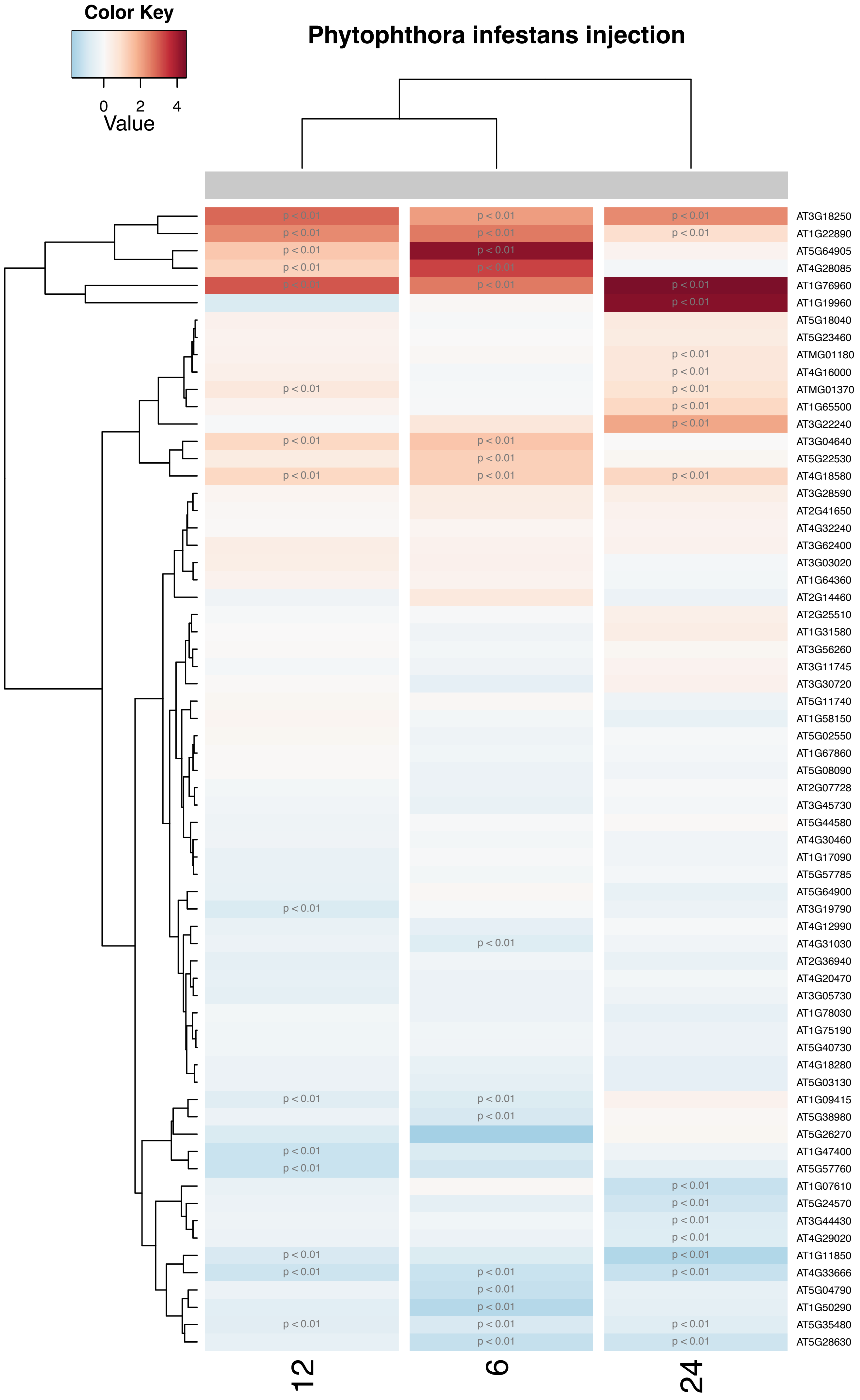
Color Key
0
2
4
Value
Phytophthora infestans injection
AT3G18250
AT1G22890
AT5G64905
AT4G28085
AT1G76960
AT1G19960
AT5G18040
AT5G23460
ATMG01180
AT4G16000
ATMG01370
AT1G65500
AT3G22240
AT3G04640
AT5G22530
AT4G18580
AT3G28590
AT2G41650
AT4G32240
AT3G62400
AT3G03020
AT1G64360
AT2G14460
AT2G25510
AT1G31580
AT3G56260
AT3G11745
AT3G30720
AT5G11740
AT1G58150
AT5G02550
AT1G67860
AT5G08090
AT2G07728
AT3G45730
AT5G44580
AT4G30460
AT1G17090
AT5G57785
AT5G64900
AT3G19790
AT4G12990
AT4G31030
AT2G36940
AT4G20470
AT3G05730
AT1G78030
AT1G75190
AT5G40730
AT4G18280
AT5G03130
AT1G09415
AT5G38980
AT5G26270
AT1G47400
AT5G57760
AT1G07610
AT5G24570
AT3G44430
AT4G29020
AT1G11850
AT4G33666
AT5G04790
AT1G50290
AT5G35480
AT5G28630
p < 0.01
12
6
24

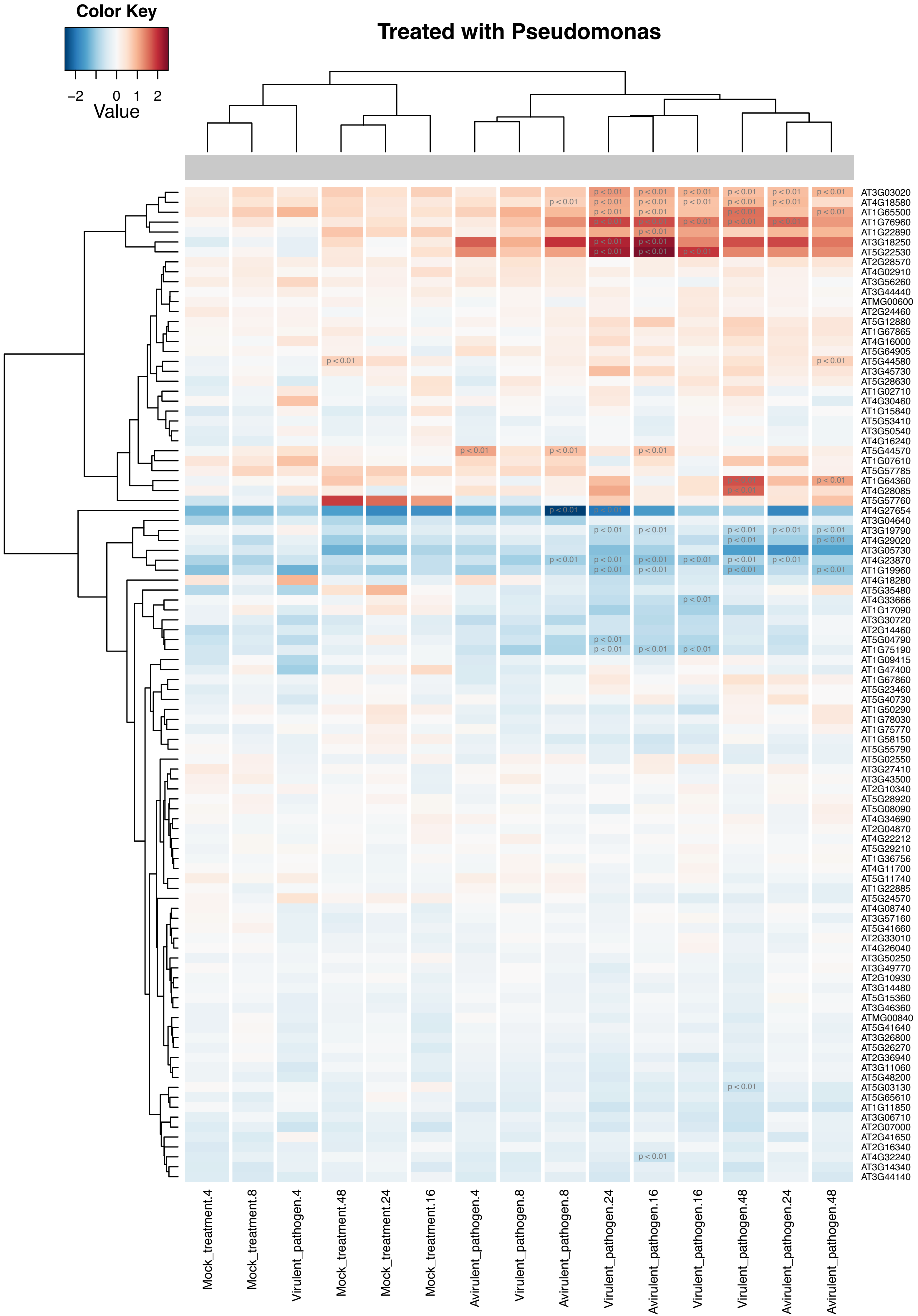
Color Key
−2 0 1 2
Value
Treated with Pseudomonas
p < 0.01
AT3G03020
AT4G18580
AT1G65500
AT1G76960
AT1G22890
AT3G18250
AT5G22530
AT2G28570
AT4G02910
AT3G56260
AT3G44440
ATMG00600
AT2G24460
AT5G12880
AT1G67865
AT4G16000
AT5G64905
AT5G44580
AT3G45730
AT5G28630
AT1G02710
AT4G30460
AT1G15840
AT5G53410
AT3G50540
AT4G16240
AT5G44570
AT1G07610
AT5G57785
AT1G64360
AT4G28085
AT5G57760
AT4G27654
AT3G04640
AT3G19790
AT4G29020
AT3G05730
AT4G23870
AT1G19960
AT4G18280
AT5G35480
AT4G33666
AT1G17090
AT3G30720
AT2G14460
AT5G04790
AT1G75190
AT1G09415
AT1G47400
AT1G67860
AT5G23460
AT5G40730
AT1G50290
AT1G78030
AT1G75770
AT1G58150
AT5G55790
AT5G02550
AT3G27410
AT3G43500
AT2G10340
AT5G28920
AT5G08090
AT4G34690
AT2G04870
AT4G22212
AT5G29210
AT1G36756
AT4G11700
AT5G11740
AT1G22885
AT5G24570
AT4G08740
AT3G57160
AT5G41660
AT2G33010
AT4G26040
AT3G50250
AT3G49770
AT2G10930
AT3G14480
AT5G15360
AT3G46360
ATMG00840
AT5G41640
AT3G26800
AT5G26270
AT2G36940
AT3G11060
AT5G48200
AT5G03130
AT5G65610
AT1G11850
AT3G06710
AT2G07000
AT2G41650
AT2G16340
AT4G32240
AT3G14340
AT3G44140
Mock_treatment.4
Mock_treatment.8
Virulent_pathogen.4
Mock_treatment.48
Mock_treatment.24
Mock_treatment.16
Avirulent_pathogen.4
Virulent_pathogen.8
Avirulent_pathogen.8
Virulent_pathogen.24
Avirulent_pathogen.16
Virulent_pathogen.16
Virulent_pathogen.48
Avirulent_pathogen.24
Avirulent_pathogen.48

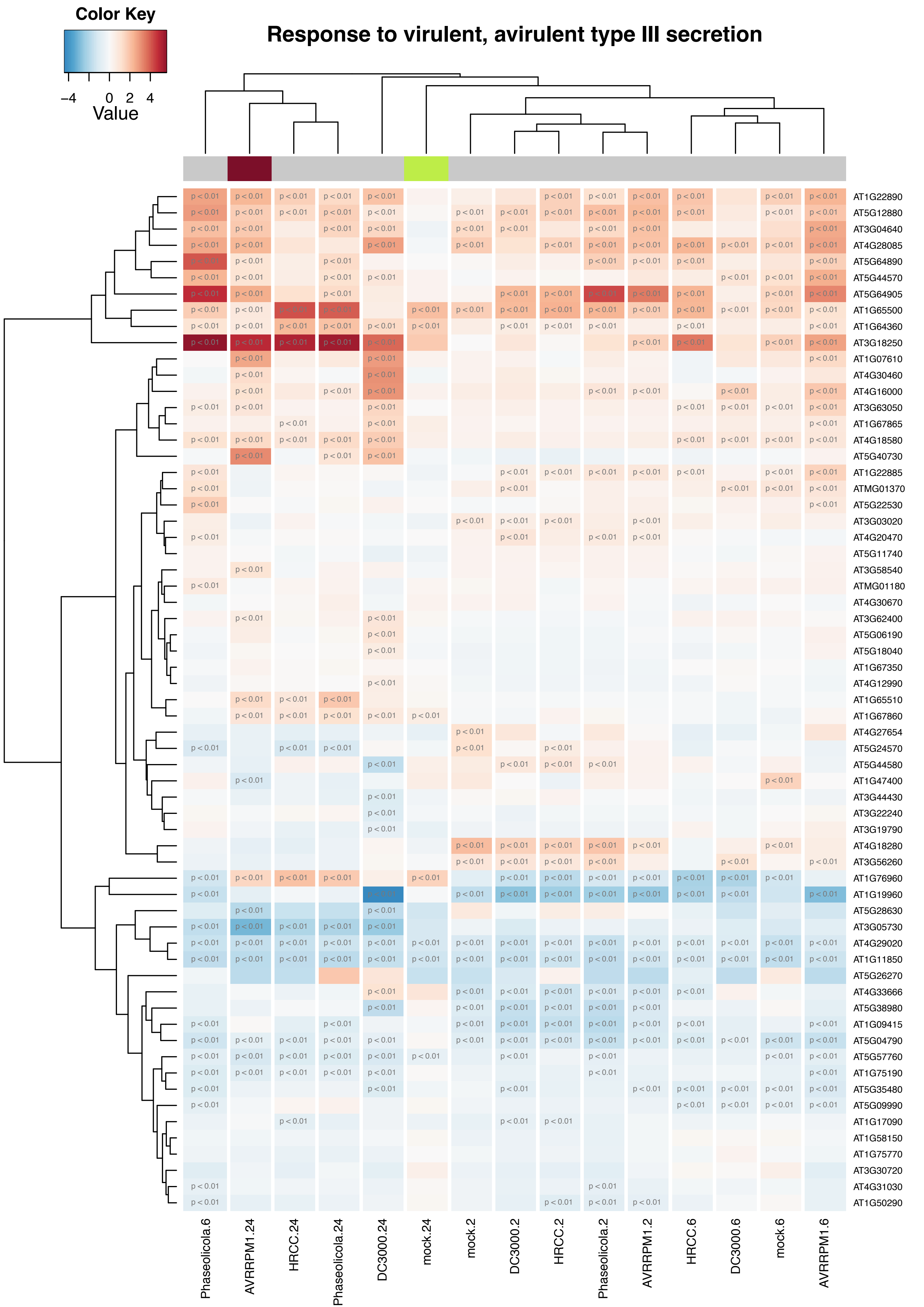

Response to virulent, avirulent type III secretion
Color Key
−4
0
2
4
Value
AT1G22890
AT5G12880
AT3G04640
AT4G28085
AT5G64890
AT5G44570
AT5G64905
AT1G65500
AT1G64360
AT3G18250
AT1G07610
AT4G30460
AT4G16000
AT3G63050
AT1G67865
AT4G18580
AT5G40730
AT1G22885
ATMG01370
AT5G22530
AT3G03020
AT4G20470
AT5G11740
AT3G58540
ATMG01180
AT4G30670
AT3G62400
AT5G06190
AT5G18040
AT1G67350
AT4G12990
AT1G65510
AT1G67860
AT4G27654
AT5G24570
AT5G44580
AT1G47400
AT3G44430
AT3G22240
AT3G19790
AT4G18280
AT3G56260
AT1G76960
AT1G19960
AT5G28630
AT3G05730
AT4G29020
AT1G11850
AT5G26270
AT4G33666
AT5G38980
AT1G09415
AT5G04790
AT5G57760
AT1G75190
AT5G35480
AT5G09990
AT1G17090
AT1G58150
AT1G75770
AT3G30720
AT4G31030
AT1G50290
Phaseolicola.6
AVRRPM1.24
HRCC.24
Phaseolicola.24
DC3000.24
mock.24
mock.2
DC3000.2
HRCC.2
Phaseolicola.2
AVRRPM1.2
HRCC.6
DC3000.6
mock.6
AVRRPM1.6
